# Doubly Metathetic NiCl_2_-Catalyzed Coupling Between Bis(2-oxazolines) and Aldehydes: A Novel Access to Bis(ester-imine) Derivatives

**DOI:** 10.3390/molecules29235756

**Published:** 2024-12-05

**Authors:** Sara Colombo, Julie Oble, Giovanni Poli, Leonardo Lo Presti, Giovanni Macetti, Alessandro Contini, Gianluigi Broggini, Marta Papis, Camilla Loro

**Affiliations:** 1 Dipartimento di Scienza e Alta Tecnologia, Università degli Studi dell’Insubria, Via Valleggio 9, 22100 Como, Italy; scolombo25@uninsubria.it (S.C.); gianluigi.broggini@uninsubria.it (G.B.); 2 IPCM, Institut Parisien de Chimie Moléculaire, CNRS, Faculté des Sciences et Ingénierie, Sorbonne Université, 4 Place Jussieu, 75005 Paris, France; julie.oble@sorbonne-universite.fr (J.O.); giovanni.poli@sorbonne-universite.fr (G.P.); 3 Dipartimento di Chimica, Università degli Studi di Milano, via Golgi 19, 20133 Milano, Italy; leonardo.lopresti@unimi.it (L.L.P.); giovanni.macetti@unimi.it (G.M.); 4 Dipartimento di Scienze Farmaceutiche, DISFARM, Università degli Studi di Milano, Via Venezian 21, 20133 Milano, Italy; alessandro.contini@unimi.it

**Keywords:** nickel catalysis, bis(2-oxazolines), aldehydes, Lewis acids, malonic esters, imines

## Abstract

The coupling between bis(2-oxazolines) and two equivalents of aromatic aldehydes in the presence of catalytic amounts of NiCl_2_ affords an ester-imine product in synthetically useful yields. This virtually unknown, 100% atom-economic transformation involves the formal metathesis between the C=N double bond of the bis(2-oxazoline) moiety, which undergoes ring-opening, and the C=O double bond of the aldehyde. The scope of this transformation is studied, and a mechanism is proposed based on DFT calculations.

## 1. Introduction

The oxazolinyl scaffold is present in several compounds that find important applications in both pharmaceuticals and innovative materials [1,2,3]. 2-Oxazolines are also efficient protecting groups and versatile ligands in transition metal asymmetric catalysis, providing a valuable alternative to the easily oxidizable phosphine ligands [4,5,6], especially as bis(2-oxazolines) [7]. Additionally, the 2-oxazolyl moiety is the precursor of several functional groups, such as carboxamides, *N*-(2-aminoethyl)acylamides, 2-aminoethyl acetates, β-aminoaldehydes and isoquinolone derivatives [8,9,10,11,12,13,14,15].

**Scheme 1:**
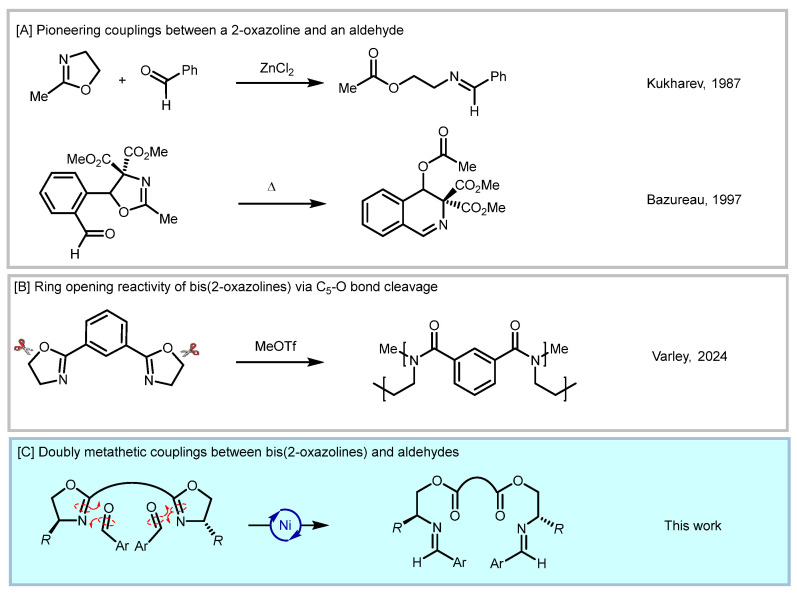
Overview of the reactivity of bis- and mono-oxazolines [16,17,23].

In particular, the coupling between a bis(2-oxazoline) and an aldehyde has been briefly reported only in two articles dating back about 30 years (Scheme 1A) [16,17,18,19]. In these papers, the reaction conditions were only superficially detailed, with no systematic investigation. This 100% atom-economic transformation involves the formal metathesis between the C=N double bond of the oxazoline moiety (which undergoes ring-opening) and the C=O double bond of the aldehyde, generating an ester-imine product. Given the interest of our group in the chemistry of nitrogen-containing five-membered heterocycles [20,21,22], we decided to further investigate this intriguing coupling, focusing in particular on bis(2-oxazolines), whose ring cleavage chemistry has been mainly addressed at O–C_5_ bond cleavage (Scheme 1B) [23,24,25]. Accordingly, we present our results on the metathetic coupling between bis(2-oxazolines) and aldehydes in this paper (Scheme 1C).

## 2. Results and Discussion

We started our study by selecting the coupling between bis(2-oxazoline) **1a** and 2-thiophenecarboxaldehyde as the model reaction (Table 1). Reacting bis(2-oxazoline) **1a** (1.0 mmol) with the aldehyde (2.0 mmol) in the presence of NiCl_2_ (10 mol%) in acetonitrile gave the bis-imino malonate ester **2a** in 53% isolated yield after 48 h at room temperature (r.t.) (Entry 1). The use of THF instead of acetonitrile resulted in a significant improvement in the isolated yield of **2a** (Entry 2). Not too unexpectedly, when the reaction was performed using equimolar amounts of **1a** and aldehyde, a low yield of **2a** was obtained, along with the mono-metathesis product **3** (Entry 3). Returning to the original 1:2 stoichiometric ratio, the use of CH_2_Cl_2_ or toluene as solvents gave again a mixture of **2a** and **3** (Entries 4 and 5). Heating the reaction at the reflux of THF resulted in a reduced yield of product **2a** (Entry 6). The reaction failed when NiCl_2_ was replaced by Ni(acac)_2_ and was less efficient when Ni(OTf)_2_ was used (Entries 7 and 8). After examining the behavior of nickel salts, other Lewis acids were evaluated as catalysts. Using PdCl_2_ or ZnCl_2_ instead of NiCl_2_ gave no reaction at room temperature and only intractable products when the reaction was heated (Entries 9–12). Cu(OTf)_2_ was also tested as a possible catalyst, given its ability to smoothly release triflic acid into the reaction medium [26,27,28,29,30]. However, its use only included a mixture of tarry products (Entry 13). Conversely, the reaction proceeded in the presence of TMSOTf, providing the ester-imine **2a** in 45% yield (Entry 14). Finally, a control experiment was carried out without any catalyst and only the unreacted substrates were restituted, thus confirming the need for a catalyst (Entry 15).

**Scheme 2:**
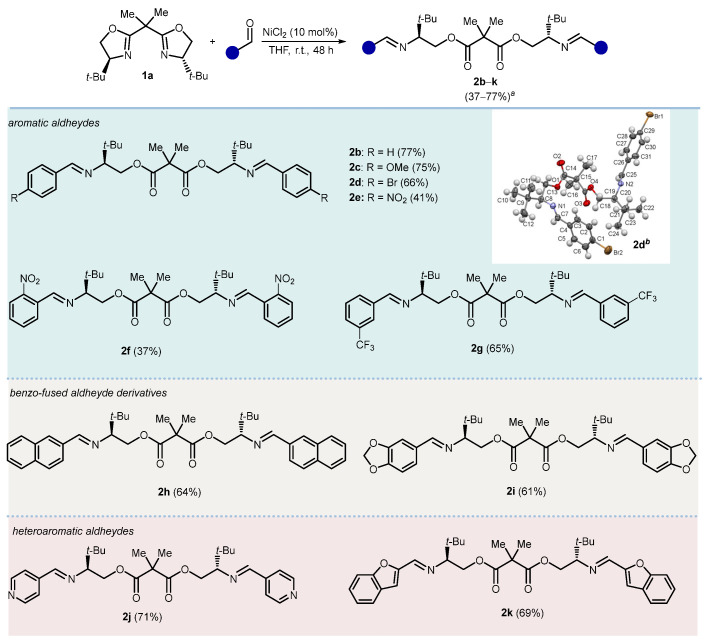
Synthesis of ester-imines from bis(2-oxazoline) **1a**. Reaction conditions: **1a** (1.0 mmol), aldehyde (2.0 mmol), NiCl_2_ (10 mol%), THF (0.1 M), r.t., 48 h. *^a^* Isolated yieldsIsolated yields. *^b^* CCDC 2385941 contains the supplementary crystallographic data for this paper. These data can be obtained free of charge from the Cambridge Crystallographic Data Centre via www.ccdc.cam.ac.uk/structures.

With the optimal conditions in hand, the substrate scope was investigated in couplings between bis(2-oxazoline) **1a** and various aldehydes (Scheme 2). Under the reaction conditions from Table 1, Entry 2 (i.e., NiCl_2_ 10 mol% in THF at r.t.), benzaldehydes bearing electron-withdrawing and electron-donating groups, as well as 2-naphthyl, piperonal, 4-pyridyl, and 2-benzofuryl-aldehydes, successfully triggered the opening of both oxazolinyl rings, leading to the formation of the corresponding ester-imines **2b**–**k**, which were isolated in moderate to satisfactory yields. The structure of the products was further confirmed by single-crystal X-ray diffraction analysis of the brominated product **2d**. The reactions carried out with cinnamaldehyde and 2-pyridinecarboxaldehyde also resulted in the complete conversion of the substrate to the ester-imines, as evidenced by the NMR spectra of the crude reaction mixtures. Unfortunately, in these two cases, it was not possible to isolate and fully characterize the products due to their instability.

**Scheme 3:**
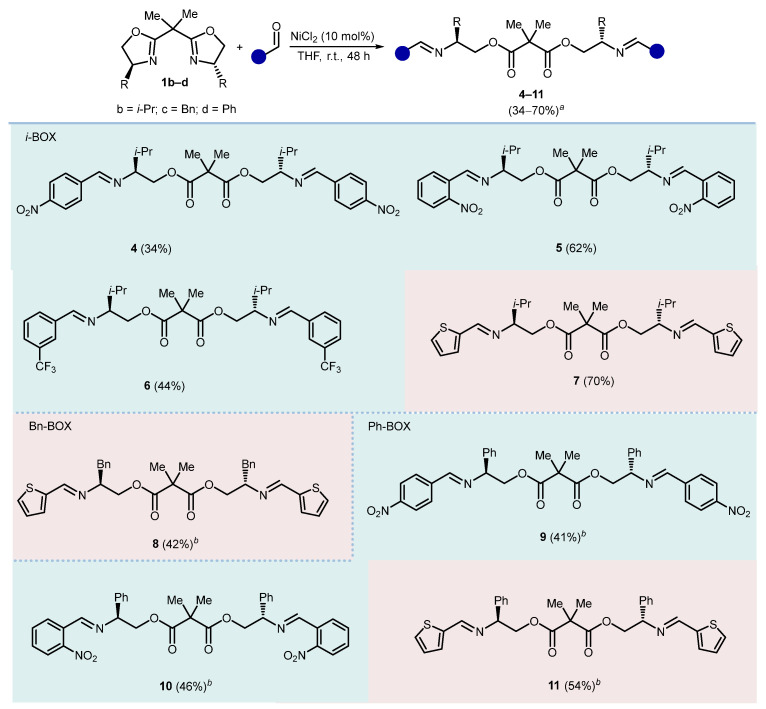
Reactivity of different bis(2-oxazolines) with different aldehydes. Reaction conditions: **1b** or **1c** (1.0 mmol), aldehyde (2.0 mmol), NiCl_2_ (10 mol%), THF (0.1 M), and r.t., 48 h. *^a^* Isolated yields. *^b^* Reaction time 72 h.

Differently substituted bis(2-oxazolines) were then examined in reactions with representative electron-poor or electron-rich aromatic aldehydes (Scheme 3). Accordingly, treatment of isopropyl, benzyl, and phenyl bis(2-oxazoline) (**1b**–**d**) with 3-nitro-, 4-nitro-, 3-trifluoromethyl benzaldehydes or 2-thiophenecarboxaldehyde under the standard conditions gave the corresponding bis(ester-imines) **4**–**11** with comparable efficiency. The bis(ester-imines) derived from benzyl bis(2-oxazoline) **1c** were found quite difficult to purify. In fact, compound **8** was isolated in 42% yield, whereas the products arising from reactions with 2-nitro and 3-trifluoromethyl benzaldehydes could not be isolated in a pure state for characterization (probably for the hydrolysis of the imine).

**Scheme 4:**
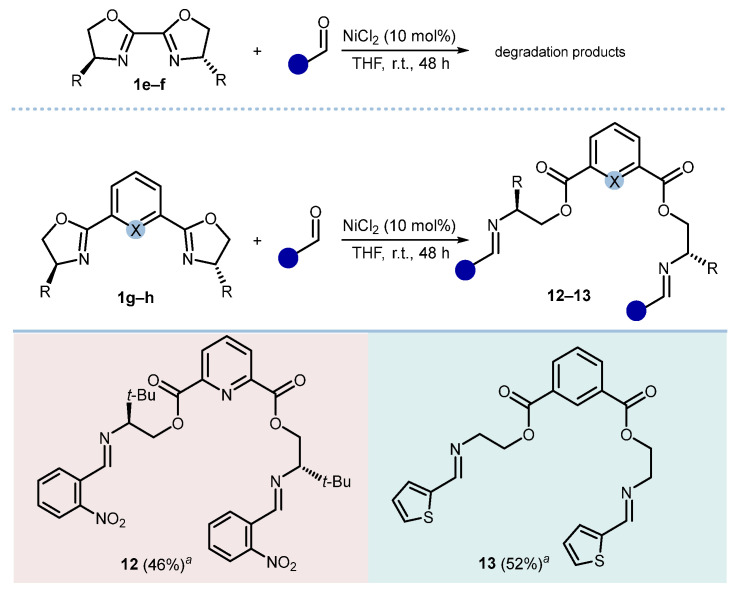
Reactivity of different bis(2-oxazolines) with different aldehydes. Reaction conditions: **1g** or **1h** (1.0 mmol), aldehyde (2.0 mmol), NiCl_2_ (10 mol%), THF (0.1 M), and r.t., 48 h. X = C, N. *^a^* Isolated yields.

After studying bis(2-oxazolines) joined through a propane-2,2-diyl moiety, we evaluated the behavior of 2,2′-bis(2-oxazolines) and of those bearing the two 2-oxazoline moieties either 1,3-linked to a phenyl ring or 2,6-linked to a pyridine ring (Scheme 4). While the former substrates (**1e,f**) were prone to degradation, **1g** and **1h** underwent the expected metathetic ring-opening to give the corresponding ester-imines **12** and **13** in acceptable yields.

**Scheme 5:**
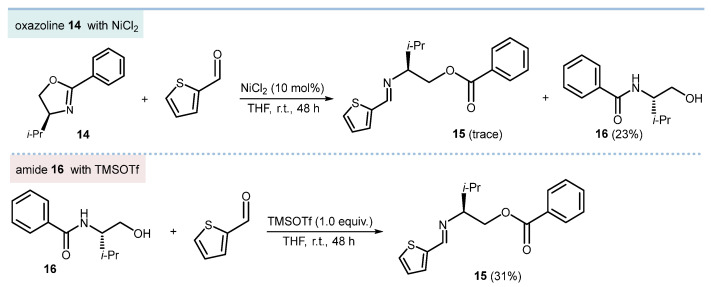
Insight into the behavior of mono-oxazolines.

The investigation continued with the evaluation of some simple 2-oxazolines under the standard conditions. These compounds exhibited different behavior compared to bis(2-oxazolines). In fact, treatment of (*S*)-4-isopropyl-2-phenyl-oxazoline **14** with 2-thiophenecarboxaldehyde under the standard reaction conditions produced a complex mixture of products after 48 h. From the mixture, amide **15** was isolated in 22% yield along with traces of the expected ester-imine **16** (Scheme 5). Since amide **15** is most likely the result of a C_5_-O cleavage of the 2-oxazolinyl moiety by the nucleophile water, it appears that passing from 2-oxazolines to bis(2-oxazolines) is associated with a reactivity shift from substitution at C_5_ (C_5_-O cleavage) to addition at C_2_ (metathesis). The unsuitability of mono-oxazolines to undergo the metathetic ring-opening was also observed with 2-phenyl or thienyl, 4-benzyl or isobutyl substituted substrates. Interestingly, treatment of **15** with 2-thiophenecarboxaldehyde in the presence of TMSOTf (1.0 equiv.) gave the ester-imine **16**, plausibly via an N→O benzoyl transfer followed by imine formation (Scheme 5).

To gain insights into the mechanism of this transformation, the model reaction between bis(2-oxazoline) **1a** and benzaldehyde was studied via DFT calculations. The geometries of reactants, products, and intermediates were optimized at the WB97XD/6-31+G(d,p) level, followed by single-point energy calculations using the WB97XD functional with the Def2TZVP basis set and CPCM(THF) solvation model (see Figures S1 and S2 and Tables S1–S3, Supplementary Materials, for details). The focus was the geometries of the intermediates—particularly the Ni chelation sites—as well as the energies. As this preliminary study was not focused on the kinetics, the transition structures were not modeled. To start the modeling with reliable assumptions, the following points were considered: (a) the presence of NiCl_2_ is crucial for the success of the reaction; (b) although water is not involved in the global transformation, its presence is essential for the success of the reaction; (c) in contrast to bis(2-oxazolines), simple 2-oxazolines do not undergo equally well this transformation. Therefore, a chelating effect of the metal by the bis(2-oxazoline) is expected; (d) NiCl_2_·(4H_2_O) **I** is assumed to be the most likely starting catalyst.

**Figure 1:**
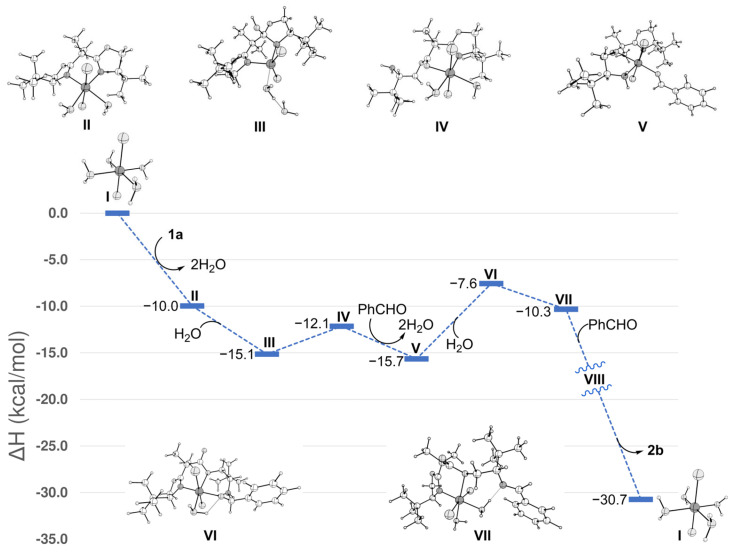
Enthalpy profile and optimized geometries of reactants, intermediates, and products along the reaction coordinate, modeled using DFT calculations at the WB97XD/Def2TZVP/CPCM(THF)//WB97XD/6-31+G(d,p) level. The reference energy corresponds to the sum of the energies of bis(2-oxazoline) **1a**, NiCl_2_·4H_2_O **I**, and two equivalents of benzaldehyde. The energies of intermediates and products were obtained by summing the energy of the main structure with that of water or benzaldehyde, ensuring the correct stoichiometry at each step.

Both singlet and triplet configurations were considered for optimizing each Ni-containing complex. However, singlet configurations consistently resulted in high-energy geometries or convergence failures. Starting from the reactants, put at Δ*H* = 0 kcal/mol (Figure 1), the exothermic (*δ*Δ*H* = −10 kcal/mol) interaction between NiCl_2_·(4H_2_O) **I** and bis(2-oxazoline) **1a** generates complex **II** with the expulsion of two molecules of water. The metal in **II** maintains hexacoordination, adopting a square bipyramidal configuration. The two chlorine atoms are apically coordinated, while the equatorial sites host the four dative ligands: two oxazolyl nitrogen atoms and two water molecules. At this point, an external water molecule adds to the C_2_ of one oxazolyl unit, generating the 2-hydroxy oxazolidine complex **III**. Unexpectedly, this complex adopts a distorted trigonal bipyramidal geometry. This switch from hexa- to penta-coordination is likely caused by steric hindrance of the tert-butyl groups, which prevent the coordination of a second molecule of water (a hexacoordinated geometry of **III** was found but was 7.5 kcal/mol less stable; see Figure S2A,B and Table S2, Supplementary Materials). Then, in a slightly endothermic step (+3 kcal/mol), the 2-hydroxy oxazolidine **III** opens up, generating the ester-primary amine **IV**. In this step, the externally appended molecule of water can reenter the nickel coordination sphere, thereby restoring the hexavalent coordination at the metal. At this point, the primary amine and benzaldehyde each coordinate with a Ni site, with the expulsion of two water molecules. This step is exothermic (*δ*Δ*H* = −3.6 kcal/mol), and the resulting primary amine **V** is the most stable intermediate along the pathway. After a likely equilibration with the decoordinated free amine (*δ*Δ*H* = +2.5 kcal/mol; see Figure S2C, Supplementary Materials), intramolecular attack of the amine to the coordinated benzaldehyde leads to the hemiaminal **VI**, with an enthalpy increase of 8.1 kcal/mol respect to **V**. This step liberates a coordination site at the metal, which can be occupied by an incoming water molecule. Interestingly, one H atom of the coordinated water is involved in an intramolecular H–bond with the hemiaminal oxygen atom (O···HO distance = 1.98 Å; O···H-O angle = 122.9 deg). Subsequent exothermal dehydration of **VI** generates the imine **VII** (*δ*Δ*H* = −2.7 kcal/mol). In this intermediate, the coordinated hemiaminal hydroxyl oxygen atom becomes part of the newly formed coordinated water molecule, which is also involved in an intramolecular H–bond with the imino nitrogen (N···HO distance = 1.74 Å; N···H-O angle = 161.2 deg). The same sequence of steps as before is then expected to transform the second oxazolinyl unit, generating Ni-coordinated product **VIII** (not computed). Finally, the entrance of two molecules of water generates the bis(imine-ester) product **2b**, closing the catalytic cycle. The enthalpy change from the starting materials to the product is significant (Δ*H* = −30.7 kcal/mol) and well supports the product formation.

**Scheme 6:**
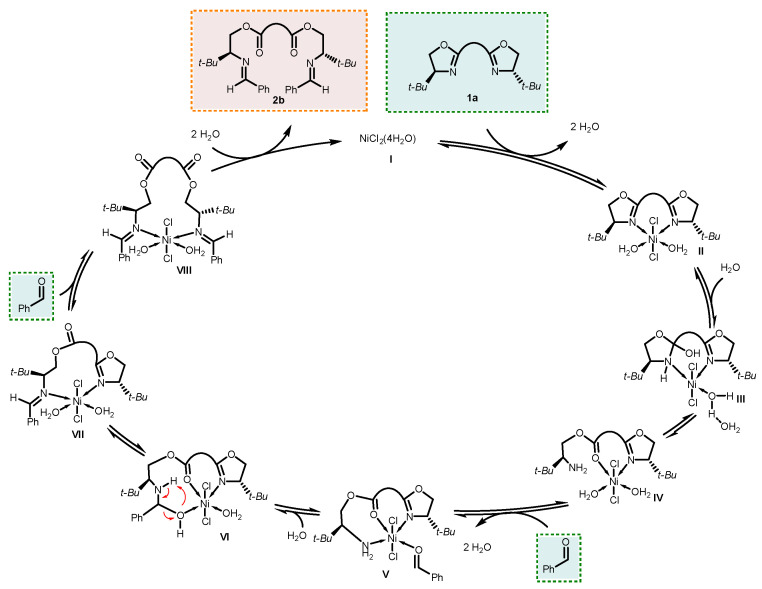
Proposed mechanism for the doubly metathetic NiCl_2_-catalyzed coupling between bis(2-oxazoline) **1a** and benzaldehyde.

Scheme 6 summarizes the overall mechanism of this doubly metathetic NiCl_2_-catalyzed coupling for the model reaction, as deduced from the above computations.

## 3. Materials and Methods

### 3.1. General Information

All available chemicals and solvents were purchased from commercial sources and were used without further purification. Thin-layer chromatography (TLC) was performed using 0.25 mm silica gel precoated plates Si 60-F254 from Merck (Darmstadt, Germany), visualized by UV-254 light and CAM staining. Purification by flash column chromatography (FCC) was conducted by using silica gel Si 60, 230–400 mesh, 0.040–0.063 mm from Merck (Darmstadt, Germany). Melting points were determined on a Stuart Scientific SMP3 from Barloworld Scientific Ltd. (Stone, UK) and are corrected. ^1^H and ^13^C NMR spectra were recorded on a Bruker Avance 400 (Billerica, MA, USA, 400 and 101 MHz, respectively); chemical shifts are given in parts per million downfield from SiMe_4_, using the residual proton (CHCl_3_ = 7.26 ppm) and carbon (CDCl_3_ = 77.0 ppm) solvent resonances as an internal reference. Coupling constant values *J* are given in Hz. High-resolution mass spectra (HRMS) were recorded using a mass spectrometer MicroTOF from Bruker (Billerica, MA, USA) with an electron spray ion source (ESI) and a TOF detector or using a mass spectrometer from Thermo Fisher Scientific (Waltham, MA, USA) with an electron spray ion source (ESI) and a LTQ Orbitrap as detector at Università degli Studi dell’Insubria (Como, Italy). FT-IR spectra were recorded on a Tensor 27 (ATR Diamond) from Bruker (Billerica, MA, USA) infrared spectrophotometer and are reported in the frequency of absorption (cm^–1^). Optical rotations were measured on a JASCO P-2000 polarimeter (Hachioji, Japan) using a 100 mm path-length cell at 589 nm and reported as follows: ${\left[\alpha \right]}_{D}^{T\;{}^{\circ}C}\;$(*c* mg/10 mL, solvent). Elemental analyses were executed on a Perkin-Elmer CHN Analyzer Series II 2400 (Waltham, MA, USA).

### 3.2. General Experimental Procedures

Starting material **14** was prepared according to the literature procedure [31]. The experimental procedure for **14** is detailed in Supplementary Materials.

#### 3.2.1. Experimental Procedure for Compounds **2a**–**k**, **4**–**13**, and **16**

To a solution of the appropriate bis(2-oxazoline) (1.0 mmol, 1 equiv.) in THF (0.1 M, 10 mL), NiCl_2_ (10 mol%, 0.1 mmol) and the aldehyde (2.0 mmol, 2 equiv.) were added and the mixture was stirred at room temperature for 24–48 h.

The mixture was concentrated, and the residue was dissolved in ethyl acetate (10 mL) and washed with water (10 mL). The organic phase was dried over Na_2_SO_4_ and the solvent was evaporated. Starting from bis(2-oxazoline) and aldehydes, the yields and physical, spectroscopic and analytical data of compounds **2a–k**, **4**–**13**, and **16** are as follows.

Details of the X-ray structural determination of **2d** are reported in Supplementary Materials.

#### 3.2.2. Experimental Procedure for Compound **15**

To a solution of the amide **16** (190 mg, 1.0 mmol, 1 equiv.) in THF (0.1 M, 10 mL), TMSOTf (180 μL, 1 equiv., 1 mmol) and the aldehyde (100 μL, 1.0 mmol, 1 equiv.) were added and the mixture was stirred at room temperature for 48 h.

The mixture was concentrated, and the residue was dissolved in ethyl acetate (10 mL) and washed with water (10 mL). The organic phase was dried over Na_2_SO_4,_ and the solvent was evaporated to make compound **15** a yellow oil.

### 3.3. Characterization Data of Synthesized Compounds

The ^1^H NMR spectra of known compound 14 is reported in Supplementary Materials.

#### Characterization Data of Final Compounds **2a**–**k**, **3**–**13** and **15**–**16**

*Bis((S)-3,3-dimethyl-2-((thiophen-2-ylmethylene)amino)tert-butyl) 2,2-dimethylmalonate* (**2a**). Starting from **1a** (294 mg) and 2-thiophenecarboxaldehyde (224 mg); FCC-AcOEt/hexane (2:3). **2a** (543 mg, 81%); white wax. ^1^H NMR (CDCl_3_, 400 MHz) δ 8.09 (s, 2H), 7.29 (d, 2H, *J* = 5.1 Hz), 7.20 (d, 2H, *J* = 4.6 Hz), 6.97 (t, 2H, *J* = 5.0 Hz), 4.29 (dd, 2H, *J* = 2.7, 10.9 Hz), 3.90 (t, 2H, *J* = 9.9 Hz), 2.85 (dd, 2H, *J* = 2.8, 9.8 Hz), 1.8 (s, 6H), 0.85 (s, 18 H); ^13^C NMR (CDCl_3_, 101 MHz) δ 172.5, 154.3, 142.7, 130.1, 128.8, 127.3, 77.8, 65.3, 49.9, 33.7, 269, 22.8. HRMS (ESI) calcd. for C_27_H_38_N_2_O_4_S_2_ [M + H^+^]: 519.2346, found: 519.2330. ${\left[\alpha \right]}_{D}^{25}$ = +38.0 (*c* 0.55 CH_2_Cl_2_).

*Bis((S)-3,3-dimethyl-2-((benzylidene)amino)tert-butyl) 2,2-dimethylmalonate* (**2b**)**.** Starting from **1a** (294 mg) and benzaldehyde (212 mg); FCC-AcOEt/hexane (2:3). **2b** (390 mg, 77%); white wax. ^1^H NMR (CDCl_3_, 400 MHz) δ 8.08 (s, 2H), 7.71–7.60 (m, 4H), 7.41–7.35 (m, 6H), 4.37 (dd, 2H, *J* = 2.7, 10.8 Hz), 3.97 (t, 2H, *J* = 9.8 Hz), 2.95 (dd, 2H, *J* = 2.8, 9.8 Hz), 1.23 (s, 6H), 0.92 (s, 18H); ^13^C NMR (CDCl_3_, 101 MHz) δ 172.5, 161.1, 136.3, 130.5, 128.5, 128.2, 78.2, 65.4, 49.9, 33.6, 26.9, 22.8. HRMS (ESI) calcd. for C_31_H_42_N_2_O_4_ [M + H^+^]: 507.3218, found: 507.3194. ${\left[\alpha \right]}_{D}^{25}$ = −23.5 (*c* 0.55 CH_2_Cl_2_).

*Bis((S)-3,3-dimethyl-2-((4-methoxybenzylidene)amino)tert-butyl) 2,2-dimethylmalonate* (**2c**). Starting from **1a** (294 mg) and 4-methoxybenzaldehyde (272 mg); FCC-AcOEt/hexane (2:3). **2a** (425 mg, 75%); ^1^H NMR (CDCl_3_, 400 MHz) δ 8.10 (s, 2H), 7.67 (d, 2H, *J* = 8.8 Hz), 6.90 (d, 2H, *J* = 8.8 Hz), 4.33 (dd, 2H, *J* = 2.7, 10.8 Hz), 3.94 (t, 2H, *J* = 9.9 Hz), 3.83 (s, 6H), 2.90 (dd, 2H, *J* = 2.8, 9.8 Hz), 1.22 (s, 6H), 0.91 (s, 18 H); ^13^C NMR (CDCl_3_, 101 MHz) δ 172.5, 161.5, 1603, 129.9, 129.3, 113.9, 78.1, 65.5, 55.3, 49.9, 33.6, 27.0, 22.8. HRMS (ESI) calcd. for C_33_H_46_N_2_O_6_ [M + H^+^]: 567.3429, found: 567.3413. ${\left[\alpha \right]}_{D}^{25}$ = −43.3 (*c* 0.5 CH_2_Cl_2_).

*Bis((S)-3,3-dimethyl-2-((4-bromobenzylidene)amino)tert-butyl) 2,2-dimethylmalonate* (**2d**). Starting from **1a** (294 mg) and 4-bromobenzaldehyde (370 mg); FCC-AcOEt/hexane (2:3). **2d** (438 mg, 66%); white wax. ^1^H NMR (CDCl_3_, 400 MHz) δ 7.76 (s, 2H), 7.33 (d, 4H, *J* = 8.5 Hz), 7.27 (d, 2H, *J* = 8.4 Hz), 4.05 (dd, 2H, *J* = 2.7, 10.9 Hz), 3.62 (t, 2H, *J* = 9.9 Hz), 2.67 (dd, 2H, *J* = 2.7, 9.8 Hz), 0.96 (s, 6H), 0.65 (s, 18H); ^13^C NMR (CDCl_3_, 101 MHz) δ 172.5, 161.5, 160.3, 129.9, 129.3, 113.9, 78.1, 65.5, 55.3, 33.6, 27.0, 22.8. Anal. calcd. for C_31_H_40_Br_2_N_2_O_4_: C, 56.04; H, 6.07; Br, 24.05; N, 4.22; O, 9.63. Found: C, 55.98; H, 6.15; Br, 24.32; N, 4.20. ${\left[\alpha \right]}_{D}^{25}$ = −77.5 (*c* 0.5 CH_2_Cl_2_).

*Bis((S)-3,3-dimethyl-2-((4-nitrobenzylidene)amino)tert-butyl) 2,2-dimethylmalonate*** (2e).** Starting from **1a** (294 mg) and 4-nitrobenzaldehyde (302 mg); FCC-AcOEt/hexane (2:3). **2e** (244 mg, 41%); white wax. ^1^H NMR (CDCl_3_, 400 MHz) δ 8.27 (d, 4H, *J* = 8.7 Hz), 8.16 (s, 2H), 7.89 (d, 4H, *J* = 8.7 Hz), 4.32 (dd, 2H, *J* = 2.7, 10.9 Hz), 3.84 (t, 2H, *J* = 10 Hz), 3.00 (dd, 2H, *J* = 2.7, 9.8 Hz), 1.22 (s, 6H), 0.90 (s, 18H); ^13^C NMR (CDCl_3_, 101 MHz) δ 172.2, 159.1, 149.1, 141.4, 128.9, 123.9, 78.3, 65.0, 49.8, 33.6, 26.8, 22.7. Anal. calcd. for C_31_H_40_N_4_O_8_: C, 62.40; H, 6.76; N, 9.39. Found: C, 62.42; H, 6.77; N, 9.39; O, 21.44. ${\left[\alpha \right]}_{D}^{25}$ = +20.7 (*c* 0.5 CH_2_Cl_2_).

*Bis((S)-3,3-dimethyl-2-((2-nitrobenzylidene)amino)tert-butyl) 2,2-dimethylmalonate* (**2f**). Starting from **1a** (294 mg) and 2-nitrobenzaldehyde (302 mg); FCC-AcOEt/hexane (2:3). **2f** (220 mg, 37%); white wax. ^1^H NMR (CDCl_3_, 400 MHz) δ 8.54 (s, 2H), 8.04 (d, 2H, *J* = 9.4 Hz), 7.97 (d, 2H, *J* = 9.4 Hz), 7.67–7.63 (m, 2H), 7.57–7.50 (m, 2H), 4.41 (dd, 2H, *J* = 2.6, 10.9 Hz), 4.11 (t, 2H, *J* = 10.9 Hz), 3.10 (dd, 2H, *J* = 2.6, 9.6 Hz), 1.27 (s, 6H), 0.96 (s, 18H); ^13^C NMR (CDCl_3_, 101 MHz) δ 172.5, 157.1, 148.9, 133.3, 130.9, 130.6, 130.1, 124.2, 78.5, 65.3, 50.1, 33.8, 26.9, 22.7. Anal. calcd. for C_31_H_40_N_4_O_8_: C, 62.40; H, 6.76; N, 9.39; O, 21.45. Found: C, 62.44; H, 6.78; N, 9.42; O, 21.48. ${\left[\alpha \right]}_{D}^{25}$ = −37.2 (*c* 0.5 CH_2_Cl_2_).

*Bis((S)-3,3-dimethyl-2-((3-trifluoromethylbenzylidene)amino)tert-butyl) 2,2-dimethylmalonate (***2g***).* Starting from **1a** (294 mg) and 3-trifluoromethylbenzaldehyde (348 mg); FCC-AcOEt/hexane (2:3). **2g** (417 mg, 65%); white wax. ^1^H NMR (CDCl_3_, 400 MHz) δ 8.12 (s, 2H), 7.98 (s, 2H), 7.90 (d, 2H, *J* = 7.7 Hz), 7.66 (d, 2H, *J* = 7.9 Hz), 7.52 (t, 2H, *J* = 7.7 Hz), 4.35 (dd, 2H, J = 2.7, 10.9 Hz), 3.89 (t, 2H, *J* = 9.6 Hz), 2.97 (dd, 2H, *J* = 2.7, 9.7 Hz), 1.23 (s, 6H), 0.90 (s, 18 H); ^13^C NMR (CDCl_3_, 101 MHz) δ 172.4, 159.6, 136.9, 131.4 (q, *J* = 1.54 Hz), 131.1 (q, *J* = 32 Hz), 129.1, 127.0 (q, *J* = 3.8 Hz), 124.8 (q, *J* = 4.2 Hz), 124.0 (q, *J* = 273 Hz), 78.1, 65.2, 49.9, 33.6, 26.8, 22.7. Anal. calcd. for C_33_H_40_F_6_N_2_O_4_: C, 61.67; H, 6.27; N, 4.36; O, 9.96. Found: C, 61.70; H, 6.30; N, 4.40; O, 9.98. ${\left[\alpha \right]}_{D}^{25}$ = −12.3 (*c* 0.5 CH_2_Cl_2_).

*Bis((S)-3,3-dimethyl-2-((naphthalen-2-ylmethylene)amino)tert-butyl) 2,2-dimethylmalonate* (**2h**). Starting from **1a** (294 mg) and 2-naphtaldehyde (312 mg); FCC-AcOEt/hexane (2:3). **2h** (388 mg, 64%); white wax. ^1^H NMR (CDCl_3_, 400 MHz) δ 9.02 (d, 2H, *J* = 8.4 Hz), 8.66 (s, 2H), 7.88 (t, 4H, *J* = 4.0 Hz), 7.80 (d, 2H, *J* = 7.2 Hz), 7.60–7.48 (m, 6H), 4.41 (dd, 2H, *J* = 2.7, 10.7 Hz), 4.07 (t, 2H, *J* = 9.8 Hz), 3.00 (dd, 2H, *J* = 2.7, 9.7 Hz), 1.23 (s, 6H), 0.94 (s, 18 H); ^13^C NMR (CDCl_3_, 101 MHz) δ 172.6, 161.3, 133.9, 131.5, 131.3, 131.0, 129.6, 128.5, 127.2, 126.1, 125.2, 124.9, 79.5, 65.6, 50.0, 33.6, 27.0, 22.8. Anal. calcd. for C_39_H_46_N_2_O_4_: C, 77.20; H, 7.64; N, 4.62; O, 10.55. Found: C, 77.31; H, 7.69; N, 4.71; O, 10.60. ${\left[\alpha \right]}_{D}^{25}$ = −37.1 (*c* 0.5 CH_2_Cl_2_).

*Bis((S)-3,3-dimethyl-2-((benzo[d]*[1,3]*dioxol-5-ylmethylene)amino)tert-butyl) 2,2-dimethylmalonate* (**2i**). Starting from **1a** (294 mg) and piperonal (300 mg); FCC-AcOEt/hexane (2:3). **2i** (362 mg, 61%); white wax. ^1^H NMR (CDCl_3_, 400 MHz) δ 7.95 (s, 2H), 7.45–7.35 (m, 2H), 7.09–7.07 (d, 2H, *J* = 8.0 Hz), 6.80 (d, 2H, *J* = 7.9 Hz), 5.98 (s, 4H), 4.34 (dd, 2H, *J* = 2.7, 10.8 Hz), 3.92 (t, 2H, *J* = 10.3 Hz), 2.90 (dd, 2H, *J* = 2.6, 9.8 Hz), 1.24 (s, 6H), 0.91 (s, 18H); ^13^C NMR (CDCl_3_, 101 MHz) δ 172.5, 160.1, 149.7, 148.2, 131.2, 124.3, 107.9, 106.8, 101.4, 77.8, 65.4, 49.9, 33.6, 26.9, 22.8. Anal. calcd. for C_33_H_42_N_2_O_8_: C, 66.65; H, 7.12; N, 4.71; O, 21.52. Found: C, 66.70; H, 7.25; N, 4.76; O, 21.61. ${\left[\alpha \right]}_{D}^{25}$ = −20.3 (*c* 0.5 CH_2_Cl_2_).

*Bis((S)-3,3-dimethyl-2-((pyridin-4-ylmethylene)amino)tert-butyl) 2,2-dimethylmalonate* (**2j**). Starting from **1a** (294 mg) and 4-pyridinecarboxaldehyde (214 mg); FCC-AcOEt/hexane (2:3). **2j** (361 mg, 71%); white wax. ^1^H NMR (CDCl_3_, 400 MHz) δ 8.70 (s, 4H), 8.06 (br s, 2H), 7.58 (br s, 4H), 4.34 (dd, 2H, *J* = 2.8, 10.9 Hz), 3.89 (t, 2H, *J* = 10.0 Hz), 3.00 (dd, 2H, *J* = 2.7, 9.8 Hz), 1.22 (s, 6H), 0.91 (s, 18H); ^13^C NMR (CDCl_3_, 101 MHz) δ 172.3, 159.4, 150.4, 142.8, 122.1, 78.3, 65.0, 49.9, 33.6, 26.9, 22.7. Anal. calcd. for C_29_H_40_N_4_O_4_: C, 68.48; H, 7.93; N, 11.01; O, 12.58. Found: C, 68.52; H, 7.98; N, 11.07; O, 12.70. ${\left[\alpha \right]}_{D}^{25}$ = −29.5 (*c* 0.5 CH_2_Cl_2_).

*Bis((S)-3,3-dimethyl-2-((benzofuran-2-ylmethylene)amino)tert-butyl) 2,2-dimethylmalonate* (**2k**). Starting from **1a** (294 mg) and 2-benzofurancarboxaldehyde (292 mg); FCC-AcOEt/hexane (2:3). **2k** (404 mg, 69%); white wax. ^1^H NMR (CDCl_3_, 400 MHz) δ 8.06 (s, 2H), 7.59 (d, 2H, *J* = 7.7 Hz), 7.56 (d, 2H, *J* = 8.1 Hz), 7.39–7.33 (m, 2H), 7.28–7.23 (m, 2H), 7.06 (s, 2H), 4.48 (dd, 2H, *J* = 2.8, 11.0 Hz), 4.11 (t, 2H, *J* = 10.2 Hz), 2.99 (dd, 2H, *J* = 2.8, 9.8 Hz), 1.24 (s, 6H), 0.92 (s, 18H); ^13^C NMR (CDCl_3_, 101 MHz) δ 172.4, 155.6, 152.5, 151.0, 127.7, 126.4, 123.3, 122.0, 112.2, 110.9, 79.3, 65.3, 50.0, 33.7, 26.9, 22.8. Anal. calcd. for C_35_H_42_N_2_O_6_: C, 71.65; H, 7.22; N, 4.77; O, 16.36. Found: C, 71.73; H, 7.36; N, 4.79; O, 16.42. ${\left[\alpha \right]}_{D}^{25}$ = +12.2 (*c* 0.5 CH_2_Cl_2_).

*Bis((S)-3,3-dimethyl-2-((4-nitrobenzylidene)amino)iso-propyl) 2,2-dimethylmalonate* (**3**). **3**, white wax. ^1^H NMR (CDCl_3_, 400 MHz) δ 8.25 (s, 1H), 7.37 (d, 1H, *J* = 5.0 Hz), 7.29 (d, 1H, *J* = 4.9 Hz), 7.05 (dd, 1H, *J* = 3.6, 5.1 Hz), 4.45 (dd, 1H, *J* = 2.6, 10.8 Hz), 4.14 (t, 1H, *J* = 9.9 Hz), 3.91–3.83 (m, 2H), 3.60 (dd, 1H, *J* = 7.6, 9.5 Hz), 2.99 (dd, 1H, *J* = 2.7, 9.8 Hz), 1.42 (s, 3H), 1.36 (s, 3H), 0.96 (s, 9H), 0.80 (s, 9H); ^13^C NMR (CDCl_3_, 101 MHz) δ 1736, 167.9, 154.2, 142.9, 129.9, 128.7, 127.2, 77.9, 75.3, 68.9, 65.7, 44.2, 33.7, 26.9, 25.7, 23.7. HRMS (ESI) calcd. for C_22_H_34_N_2_O_3_S [M + H^+^]: 407.2363, found: 407.2350. ${\left[\alpha \right]}_{D}^{25}$ = −236.6 (c 0.55 CH_2_Cl_2_).

*Bis((S)-3,3-dimethyl-2-((4-nitrobenzylidene)amino)iso-propyl) 2,2-dimethylmalonate* (**4**). Starting from **1b** (266 mg) and 4-nitrobenzaldehyde (302 mg); FCC-AcOEt/hexane (2:3). **4** (193 mg, 34%); white wax. ^1^H NMR (CDCl_3_, 400 MHz) δ 7.26 (d, 4H, *J* = 8.8 Hz), 8.20 (s, 2H), 7.89 (d, 4H, *J* = 8.8 Hz), 4.27 (dd, 2H, *J* = 3.6, 10.9 Hz), 3.98 (dd, 2H, *J* = 8.9, 11.0 Hz), 3.18–3.13 (m, 2H), 1.93–1.83 (m, 2H), 1.25 (s, 6H), 0.91 (d, 6 H, *J* = 6.8 Hz), 0.89 (d, 6H, *J* = 6.8 Hz); ^13^C NMR (CDCl_3_, 101 MHz) δ 172.2, 159.2, 149.1, 141.4, 128.9, 123.9, 75.0, 66.3, 49.9, 30.5, 22.7, 19.5, 18.5. Anal. calcd. for C_29_H_36_N_4_O_8_: C, 61.26; H, 6.38; N, 9.85; O, 22.51. Found: C, 61.43; H, 6.61; N, 9.94; O, 22.72. ${\left[\alpha \right]}_{D}^{25}$ = +17.5 (*c* 0.5 CH_2_Cl_2_).

*Bis((S)-3,3-dimethyl-2-((2-nitrobenzylidene)amino)iso-propyl) 2,2-dimethylmalonate* (**5**). Starting from **1b** (266 mg) and 2-nitrobenzaldehyde (302 mg); FCC-AcOEt/hexane (2:3). **5** (352 mg, 62%); white wax. ^1^H NMR (CDCl_3_, 400 MHz) δ 8.58 (s, 2H), 8.01 (dd, 4H, *J* = 4.6, 9.2 Hz), 7.65 (t, 2H, *J* = 7.6 Hz), 7.56 (t, 2H, *J* = 6.5 Hz), 4.35 (dd, 2H, *J* = 3.5, 11.0 Hz), 4.16 (dd, 2H, *J* = 8.8, 11.1 Hz), 3.25–3.20 (m, 2H), 1.97–1.89 (m, 2H), 1.31 (s, 6H), 0.95 (d, 6H, *J* = 7.9 Hz), 0.93 (d, 6H, *J* = 7.0 Hz); ^13^C NMR (CDCl_3_, 101 MHz) δ 172.4, 157.5, 133.5, 131.1, 130.7, 130.1, 124.3, 75.2, 66.6, 50.1, 30.5, 22.8, 19.6, 18.6. Anal. calcd. for C_29_H_36_N_4_O_8_: C, 61.26; H, 6.38; N, 9.85; O, 22.51. Found: C, 61.35; H, 6.51; N, 9.92; O, 22.63. ${\left[\alpha \right]}_{D}^{25}$ = −6.4 (*c* 0.5 CH_2_Cl_2_).

*Bis((S)-3,3-dimethyl-2-((trifluoromethyl)benzylidene)amino)iso-propyl) 2,2-dimethylmalonate* (**6**). Starting from **1b** (266 mg) and 3-trifluoromethylbenzaldehyde (348 mg); FCC-AcOEt/hexane (2:3). **6** (270 mg, 44%); white wax. ^1^H NMR (CDCl_3_, 400 MHz) δ 8.16 (s, 2H), 7.99 (s, 2H), 7.89 (d, 2H, *J* = 7.8 Hz), 7.65 (d, 2H, *J* = 7.7 Hz), 7.52 (t, 2H, *J* = 7.7 Hz), 4.28 (dd, 2H, *J* = 3.6, 10.9 Hz), 3.98 (dd, 2H, *J* = 8.9, 10.9 Hz), 3.13–3.08 (m, 2H), 1.91–1.83 (m, 2H), 1.27 (s, 6H), 0.90 (d, 6H, *J* = 6.1 Hz), 0.89 (d, 6H, *J* = 6.1 Hz); ^13^C NMR (CDCl_3_, 101 MHz) δ 172.3, 159.8, 136.8, 131.4, 131.2 (q, *J* = 32.7 Hz), 129.1, 127.1 (q, *J* = 3.8 Hz), 124.8 (q, *J* = 3.9 Hz), 123.9 (q, *J* = 273.6 Hz) 74.9, 66.5, 49.9, 30.4, 22.7, 19.6, 18.5. Anal. calcd. for C_31_H_36_F_6_N_2_O_4_: C, 60.58; H, 5.90; F, 18.55; N, 4.56; O, 10.41. Found: C, 60.71; H, 5.96; F, 18.76; N, 4.64; O, 10.49. ${\left[\alpha \right]}_{D}^{25}$ = +44.8 (*c* 0.5 CH_2_Cl_2_).

*Bis((S)-3,3-dimethyl-2-(thiophen-2-ylmethylene)amino)iso-propyl) 2,2-dimethylmalonate* (**7**). Starting from **1b** (266 mg) and 2-thiophenecarboxaldehyde (224 mg); FCC-AcOEt/hexane (2:3). **7** (343 mg, 70%); white wax. ^1^H NMR (CDCl_3_, 400 MHz) δ 8.21 (s, 2H), 7.36 (d, 2H, *J* = 5.0 Hz), 7.28 (d, 2H, *J* = 4.8 Hz), 7.04 (t, 2H, *J* = 5.0 Hz), 4.30 (dd, 2H, *J* = 3.8, 10.9 Hz), 4.03 (dd, 2H, *J* = 8.8, 10.9 Hz), 3.07–3.02 (m, 2H), 1.92–1.83 (m, 2H), 1.29 (s, 6H), 0.92 (d, 6H, *J* = 6.7 Hz), 0.89 (d, 6H, *J* = 6.7 Hz); ^13^C NMR (CDCl_3_, 101 MHz) δ 172.5, 154.6, 142.4, 130.3, 128.8, 127.3, 74.7, 66.6, 49.9, 30.4, 22.8, 19.7, 18.9. HRMS (ESI) calcd. for C_25_H_34_N_2_O_4_S_2_ [M + H^+^]: 491.2033, found: 491.2013. ${\left[\alpha \right]}_{D}^{25}$ = +8.7 (*c* 0.5 CH_2_Cl_2_).

*Bis((S)-3-phenyl-2-((thiophen-2-ylmethylene)amino)propyl) 2,2-dimethylmalonate* (**8**). Starting from **1c** (362 mg) and 2-thiophenecarboxaldehyde (224 mg); FCC-AcOEt/hexane (2:3). **8** (246 mg, 42%); white wax. ^1^H NMR (CDCl_3_, 400 MHz) δ 7.95 (s, 2H), 7.35 (d, 2H, *J* = 5.2 Hz), 7.22–7.20 (m, 4H), 7.18–7.10 (m, 8H), 7.01 (t, 2H, *J* = 4.8 Hz), 4.23 (dd, 2H, *J* = 4.6, 10.8 Hz), 4.13 (t, 2H, *J* = 8.1 Hz), 3.63–3.52 (m, 2H), 2.92–2.81 (m, 4H), 1.33 (s, 6H); ^13^C NMR (CDCl_3_, 101 MHz) δ 172.3, 155.1, 142.1, 137.9, 130.7, 129.7, 129.0, 128.3, 127.3, 126.4, 70.4, 67.4, 49.9, 39.0, 22.8. Anal. calcd. for C_33_H_34_N_2_O_4_S_2_: C, 67.55; H, 5.84; N, 4.77; O, 10.91; S, 10.93. Found: C, 67.59; H, 5.92; N, 4.83; O, 10.98; S, 10.94. ${\left[\alpha \right]}_{D}^{25}$ = −37.1 (*c* 0.5 CH_2_Cl_2_).

*Bis((S)-2-((4-nitrobenzylidene)amino)-2-phenylethyl) 2,2-dimethylmalonate* (**9**). Starting from **1d** (334 mg) and 4-nitrobenzaldehyde (302 mg); FCC-AcOEt/hexane (2:3). **9** (261 mg, 41%); white wax. ^1^H NMR (CDCl_3_, 400 MHz) δ 8.33 (s, 2H), 8.23 (d, 4H, *J* = 8.8 Hz), 7.93 (d, 4H, *J* = 8.8 Hz), 7.39–7.29 (m, 10H), 4.56 (dd, 2H, *J* = 4.2, 9.3 Hz), 4.26 (dd, 2H, *J* = 4.2, 11.0 Hz), 4.11 (dd, 2H, *J* = 9.3, 11.0 Hz), 1.28 (s, 6H); ^13^C NMR (CDCl_3_, 101 MHz) δ 172.0, 159.8, 149.2, 138.7, 130.5, 129.1, 128.8, 128.2, 127.2, 124.3, 123.9, 73.1, 68.5, 49.8, 22.7. Anal. calcd. for C_35_H_32_N_4_O_8_: C, 66.03; H, 5.07; N, 8.80; O, 20.10. Found: C, 66.05; H, 5.21; N, 8.96; O, 20.27. ${\left[\alpha \right]}_{D}^{25}$ = −46.8 (*c* 0.5 CH_2_Cl_2_).

*Bis((S)-2-((2-nitrobenzylidene)amino)-2-phenylethyl) 2,2-dimethylmalonate* (**10**). Starting from **1d** (334 mg) and 2-nitrobenzaldehyde (302 mg); FCC-AcOEt/hexane (2:3). **10** (292 mg, 46%); white wax. ^1^H NMR (CDCl_3_, 400 MHz) δ 8.74 (s, 2H), 8.12 (d, 2H, *J* = 7.8 Hz), 7.95 (d, 2H, *J* = 8.3 Hz), 7.64 (t, 2H, *J* = 7.6 Hz), 7.56–7.50 (m, 2H), 7.43–7.34 (m, 10 H), 4.64 (dd, 2H, *J* = 4.6, 8.7 Hz), 4.35 (dd, 2H, *J* = 4.6, 11.0 Hz), 3.74 (t, 2H, *J* = 6.6 Hz), 1.31 (s, 6H); ^13^C NMR (CDCl_3_, 101 MHz) δ 172.2, 157.9, 139.0, 133.4, 130.9, 130.0, 128.7, 127.9, 127.3, 124.3, 73.1, 68.4, 53.4, 22.7. Anal. calcd. for C_35_H_32_N_4_O_8_: C, 66.03; H, 5.07; N, 8.80; O, 20.10. Found: C, 66.25; H, 5.18; N, 8.99; O, 20.33. ${\left[\alpha \right]}_{D}^{25}$ = +15.1 (*c* 0.5 CH_2_Cl_2_).

*Bis((S)-3,3-dimethyl-2-(thiophen-2-ylmethylene)amino)iso-propyl) 2,2-dimethylmalonate* (**11**). Starting from **1d** (334 mg) and 2-thiophenecarboxaldehyde (224 mg); FCC-AcOEt/hexane (2:3). **11** (301 mg, 54%); white wax. ^1^H NMR (CDCl_3_, 400 MHz) δ 8.26 (s, 2H), 7.35–7.26 (m, 14 H), 6.94 (t, 2H, *J* = 5.0 Hz), 4.42 (dd, 2H, *J* = 4.5, 8.8 Hz), 4.25 (dd, 2H, *J* = 4.6, 10.9 Hz), 4.13 (dd, 2H, *J* = 8.9, 11.1 Hz), 1.22 (s, 6H); ^13^C NMR (CDCl_3_, 101 MHz) δ 172.3, 155.2, 139.7, 130.9, 129.3, 128.6, 127.7, 127.5, 127.3, 72.4, 68.7, 22.7. Anal. calcd. for C_31_H_30_N_2_O_4_S_2_: C, 66.64; H, 5.41; N, 5.01; O, 11.45; S, 11.48. Found: C, 66.75; H, 5.54; N, 5.22; O, 11.61; S, 11.57. ${\left[\alpha \right]}_{D}^{25}$ = −8.7 (*c* 0.5 CH_2_Cl_2_).

*Bis((S)-3,3-dimethyl-2-((2-nitrobenzylidene)amino)butyl) pyridine-2,6-dicarboxylate* (**12**). Starting from **1g** (329 mg) and 2-nitrobenzaldehyde (302 mg); FCC-AcOEt/hexane (2:3). **12** (290 mg, 46%); white wax. ^1^H NMR (CDCl_3_, 400 MHz) δ 8.65 (s, 2H), 8.14 (d, 2H, *J* = 7.8 Hz), 8.05 (d, 2H, *J* = 6.3 Hz), 7.94 (d, 2H, *J* = 6.9 Hz), 7.88 (t, 2H, *J* = 7.8 Hz), 7.63 (t, 2H, *J* = 6.4 Hz), 7.52 (t, 2H, *J* = 6.6 Hz), 4.75 (dd, 2H, *J* = 2.6, 10.9 Hz), 4.55 (t, 2H, *J* = 9.6 Hz), 3.36 (dd, 2H, *J* = 2.7, 9.6 Hz), 1.06 (s, 18H); ^13^C NMR (CDCl_3_, 101 MHz) δ 164.1, 157.6, 148.3, 138.1, 133.4, 131.2, 130.6, 130.2, 129.6, 124.5, 124.1, 78.5, 65.9, 33.9, 27.1. Anal. calcd. for C_33_H_37_N_5_O_8_: C, 62.75; H, 5.90; N, 11.09; O, 20.26. Found: C, 62.80; H, 5.97; N, 11.22; O, 20.37. ${\left[\alpha \right]}_{D}^{25}$ = +27.1 (*c* 0.5 CH_2_Cl_2_).

*Bis(2-((thiophen-2-ylmethylene)amino)ethyl) isophthalate* (**13**). Starting from **1h** (216 mg) and 2-thiophenecarboxaldehyde (224 mg); FCC-AcOEt/hexane (2:3). **13** (229 mg, 52%); white wax. ^1^H NMR (CDCl_3_, 400 MHz) δ 8.55 (s, 1H), 8.36 (s, 2H), 8.09 (d, 2H, *J* = 1.8, 7.8 Hz), 7.40 (t, 1H, *J* = 7.7 Hz), 7.34 (d, 2H, *J* = 5.0 Hz), 7.28 (d, 2H, *J* = 3.7 Hz), 7.00 (dd, 2H, *J* = 3.5, 5.0 Hz), 4.55 (t, 4H, *J* = 5.6 Hz), 3.86 (t, 4H, *J* = 4.9 Hz); ^13^C NMR (CDCl_3_, 101 MHz) δ 165.6, 156.6, 133.8, 131.0, 130.7, 130.6, 129.3, 128.6, 128.3, 127.4, 64.5, 59.4. Anal. calcd. for C_22_H_20_N_2_O_4_S_2_: C, 59.98; H, 4.58; N, 6.36; O, 14.53; S, 14.56. Found: C, 60.04; H, 4.71; N, 6.42; O, 14.62; S, 14.78.

*(S)-3-Methyl-2-((thiophen-2-ylmethylene)amino)butyl benzoate* (**15**). Starting from **14** (189 mg) and 2-thiophenecarboxaldehyde (112 mg, 1 equiv.); FCC-AcOEt/hexane (2:3). **15** (93 mg, 31%); yellow oil. ^1^H NMR (CDCl_3_, 400 MHz) δ 8.37 (s, 1H), 7.97 (d, 2H, *J* = 7.0 Hz), 7.52 (t, 1H, *J* = 7.3 Hz), 7.40–7.36 (m, 4H), 7.07 (t, 1H, *J* = 5.0 Hz), 4.68 (dd, 1H, *J* = 3.9, 11.0 Hz), 4.36 (dd, 1H, *J* = 8.7, 11.0 Hz), 3.29–3.24 (m, 1H), 2.13–2.01 (m, 1H), 1.02 (d, 3H, *J* = 6.7 Hz), 0.99 (d, 3H, *J* = 6.7 Hz); ^13^C NMR (CDCl_3_, 101 MHz) δ 166.4, 154.5, 142.5, 133.1, 132.9, 130.3, 129.5, 128.9, 128.3, 127.3, 75.2, 66.5, 30.6, 19.7, 18.8. Anal. calcd. for C_17_H_19_NO_2_S: C, 67.75; H, 6.35; N, 4.65; O, 10.62; S, 10.64. Found: C, 67.89; H, 6.47; N, 4.69; O, 10.68; S, 10.72. ${\left[\alpha \right]}_{D}^{25}$ = +63.4 (*c* 0.5 CH_2_Cl_2_).

*(S)-N-(1-Hydroxy-3-methylbutan-2-yl)benzamide* (**16**). ^1^H NMR (CDCl_3_, 400 MHz) δ 7.78 (d, 2H, *J* = 7.0 Hz), 7.53–7.49 (m, 1H), 7.45–7.41 (m, 2H), 6.35 (d, 1H, *J* = 5.7 Hz), 3.98–3.91 (m, 1H), 3.84–3.71 (m, 2H), 2.07−1.98 (m, 1H), 1.04 (d, 3H, *J* = 6.8 Hz), 1.02 (d, 3H, *J* = 6.8 Hz). The characterization of **16** is consistent with that reported in the literature [32].

The detailed ^1^H, ^13^C NMR spectra for all compounds are provided in the Supplementary Materials.

### 3.4. Computational Methods

The structure of product **2b** was initially constructed in MOE [33] based on the X-ray structure of compound **2d**, followed by energy minimization using the MMFF94x force field [34] in the gas phase. A conformational search using the LowModeMD method in MOE was performed at the same level of theory with Hydrogen Mass Repartitioning (HMR), a Rejection Limit of 1000, and an Iteration Limit of 100,000, with default settings for other parameters. The 21 resulting geometries were reoptimized by density functional theory (DFT) with the ωB97X-D functional, which includes empirical dispersion corrections [35] and the 6-31+G(d,p) basis set, shown effective in similar studies [36]. Single-point energy (SP) calculations were then performed using the same function with the Def2TZVP basis set [37] and the CPCM solvation model for THF [38]. The geometry resembling the X-ray structure of compound **2d** (product2b_C0 in Table S3, Supplementary Materials) was the most stable, supporting the chosen methodology. NiCl_2_·4H_2_O was constructed and optimized in both singlet (multiplicity = 1) and triplet (multiplicity = 3) states. The singlet state converged to a square planar geometry with two water dissociating, while the triplet state converged to a square bipyramidal geometry, using all six coordination sites on Ni. This triplet configuration was 16.1 kcal/mol more stable and was thus used for all Ni chelates. Geometries involving Ni were manually constructed based on chemical insight, as reliable conformational searches for metal chelates could not be performed. Several conformations and coordination patterns were evaluated for each intermediate, and the most stable were selected for discussion. All geometry optimizations and SP energy calculations were performed using Gaussian16 [39].

## 4. Conclusions

In summary, we have studied the coupling between bis(2-oxazolines) and aromatic aldehydes, which, in the presence of catalytic amounts of NiCl_2_, affords ester-imine products in synthetically useful yields. This almost unknown transformation features 100% atom economy and involves the formal metathesis between the C=N double bond of the bis(2-oxazoline) moiety and the C=O double bond of the aldehyde. Future synthetic applications of this intriguing reaction are planned in future studies.

## Figures and Tables

**Table 1:** Optimization of the reaction conditions *^a^*.

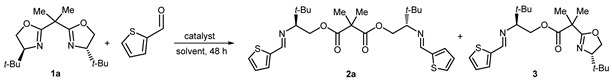				
Entry	Catalyst	Solvent	Temp. °C	Product (Yield) *^b^*
1	NiCl_2_	MeCN	r.t.	**2a** (53%)
**2**	**NiCl_2_**	**THF**	**r.t.**	**2a (81%)**
3 *^c^*	NiCl_2_	THF	r.t.	**2a** (36%) + **3** (42%)
4	NiCl_2_	CH_2_Cl_2_	r.t.	**2a** (38%) + **3** (23%)
5	NiCl_2_	Toluene	r.t.	**2a** (28%) + **3** (32%)
6 *^d^*	NiCl_2_	THF	reflux	**2a** (68%)
7	Ni(acac)_2_	THF	r.t.	not detected
8	Ni(OTf)_2_	THF	r.t	**2a** (25%) + **3** (38%)
9	PdCl_2_	THF	r.t.	not detected
10 *^d^*	PdCl_2_	THF	reflux	complex mixture
11	ZnCl_2_	THF	r.t.	not detected
12 *^d^*	ZnCl_2_	THF	reflux	complex mixture
13	Cu(OTf)_2_	THF	r.t.	complex mixture
14	TMSOTf	THF	r.t.	**2a** (45%)
15	-	THF	r.t.	not detected

*^a^* Reaction conditions: **1a** (1.0 mmol), thiophene-2-carboxaldheyde (2.0 mmol.), catalyst (10 mol%), solvent (0.1 M), 48 h. *^b^* Isolated yields. *^c^* Reaction performed using 1.0 mmol of **1a** and thiophene-2-carboxaldheyde. *^d^* Reaction time 24 h in oil bath.

## Data Availability

Data are contained within this article and Supplementary Materials.

## Supplementary Materials

The following supporting information can be downloaded at: https://www.mdpi.com/article/10.3390/molecules29235756/s1, Figure S1: Enlarged picture of optimized geometries of reactants, intermediates and products discussed in Figure 1. A: catalyst NiCl_2_·4H_2_O, reactants **1a**, benzaldehyde and water. B–G: intermediates II–VII, respectively. J: product **2b**; Table S1: Average distances and angles involving Ni atom. Angles involving Ni with O or N are generally measured between adjacent atoms unless otherwise specified; Figure S2: Alternative configurations for IntIII (A: pentavalent, and B: hexavalent; ΔHpenta-hexa= 7.5 kcal/mol), and alternative structure for IntV (C) where the amine is free and replaced by a water molecule in coordinating Ni; Table S2: Energies and corrections of each compound considered in this study; Table S3: Energies and corrections of all the conformations of product **2b** obtained by a conformational search using molecular mechanics and the MMFF94x force field in the gas phase, successively reoptimized at the WB97XD/Def2TZVP/CPCM(THF)//WB97XD/6-31+G(d,p) level; Figure S3: X-ray quality specimen of **2d**. The crystal has needle habit and is transparent and colorless with dimensions of 0.105 × 0.015 × 0.015 mm^3^; Figure S4: Up: Asymmetric unit of **2d** at RT, with the atom-numbering scheme. Thermal ellipsoids of non-H atoms were drawn at the 25% probability level. The usual color code was employed for atoms (grey: C; white: H; blue: N; red: O; dark yellow: Br). Down: Molecular structure of **2d**, with the Cahn–Ingold–Prelog descriptors highlighted; Table S4: CH···O contacts in **2d**; Figure S5: Crystal packing of **2d** at RT, as seen (a) along the a-cell axis; (b) the c-cell axis; (c) the b-cell axis. Color code as in Figure S4. The crystallographic reference system is also shown. Hydrogen atoms were omitted for clarity. References [31,40,41] are cited in the Supplementary Materials.

## References

[1] Dargaville T.R., Park J.R., Hoodenboom R. (2018). Poly(2-oxazoline) hydrogels: State-of-the-art and emerging applications. Macromol. Biosci..

[2] Yoshida M., Onda Y., Masuda Y., Doi T. (2016). Potent oxazoline analog of Apratoxin C; synthesis, biological evaluation, and conformational analysis. PeptideScience.

[3] Yadav P.N., Beveridge R.E., Blay J., Boyd A.R., Chojnacka M.W., Decken A., Deshpande A.A., Gardiner M.G., Hambley T.W., Hughes M.J. (2011). Platinum-oxazole complexes as anti-cancer agents: Syntheses, characterization and initial biological studies. Med. Chem. Commun..

[4] Connon R., Roche B., Rokadem B.V., Guiry P.J. (2021). Further developments and application of oxazoline-containing ligands in asymmetric catalysis. Chem. Rev..

[5] McManus A.H., Guiry P.J. (2004). Recent developments in the application of oxazoline-containing ligands in asymmetric catalysis. Chem. Rev..

[6] Gomez M., Muller G., Rocamora M. (1999). Coordination chemistry of oxazoline ligands. Coord. Chem. Rev..

[7] Desimoni G., Faita G., Jørgensen K.A. (2006). C2-Symmetric Chiral Bis (Oxazoline) Ligands in Asymmetric Catalysis. Chem. Rev..

[8] Deng S.K., Chen H.C., Liu Y.-F., Ji H.T., Shen J. (2024). Rin-opening phosphanylation of oxazolines. Tetrahedron Lett..

[9] Hess A., Guelen H.C., Alandini N., Mourati A., Guersoy Y.C., Knochel P. (2022). Preparation of polyfunctionalized aromatic nitriles from aryl oxazolines. Chem. Eur. J..

[10] Lin Q., Zhang S., Li B. (2020). KO*t*-Bu-Promoted selective ring-opening *N*-alkylation of 2-oxazolines to access 2-aminoethyl acetates and *N*-substituted thiazolidinones. Beilstein J. Org. Chem..

[11] Yang Z., Jie L., Yao Z., Yang Z., Cui X. (2019). Rhodium (III)-catalyzed synthesis of *N*-(2-acetoxyalkyl) isoquinolones from oxazolines and alkynes through C-N bond formation and ring-opening. Adv. Synth. Catal..

[12] Guan D., Luan H.X., Patiguli M., Jiao Q.J., Yun Q.Q., Chen Q.S., Xu C.J., Nie X.B., Hu F.P., Huang G.S. (2019). Metal–free Efficient Method for the Synthesis of N-(2-haloethyl)benzamides through the Ring-opening of 2-oxazolines. ChemistrySelect.

[13] Qiao K., Yuan X., Wan L., Zheng M.-W., Zhang D., Fan B.B., Di Z.C., Fang Z., Guo K. (2017). Highly efficient synthesis of β-nitrate ester carboxamides through the ring-opening of 2-oxazolines. Green Chem..

[14] Gutmann B., Roduit J.P., Roberge D., Kappe C.O. (2011). A two-step continuous-flow synthesis of *N*-(2-aminoethyl) acylamides through ring-opening/hydrogenation of oxazolines. Chem. Eur. J..

[15] Laitar D.S., Kramer J.W., Whiting B.T., Lobkovsky E.B., Coates G.W. (2009). β-Amidoaldehydes via oxazoline hydroformylation. Chem. Commun..

[16] Kukharev B.F., Stankevich V.K., Terent’eva V.P., Kukhareva V.A. (1987). Reaction of oxazolines with aldehydes. Zh. Org. Khim..

[17] Lerestif J.M., Toupet L., Sindandhit S., Tonnard F., Bazureau J.P., Hamelin J.A. (1997). New route to 2-oxazolines, bis-oxazolines, and 2-imidazoline-5-ones from imidates using solvent-free cycloadditions: Synthesis, chemical properties, and PM3 MO calculations. Tetrahedron.

[18] Maguet M., Poirier Y., Guglielmetti R. (1978). Spiropyrannes et mérocyanines en séries azahétérocycliques saturées. Bull. Soc. Chem. Fr..

[19] Lerestif J.M., Feuillet S., Bazureau J.P., Hamelin J. (1999). Novel Synthesis of Protected Methyl 4-Hydroxy-1,2,3,4-tetrahydroisoquinoline-3-carboxylate via Cleavage of Functionalized Dihydrooxazoles (Oxazolines). J. Chem. Res..

[20] Molteni L., Beccalli E.M., Castoldi L., Broggini G., Loro C. (2023). Methanol as a C1 Source for the Synthesis of 1,3-Polyheterocyclic Systems. Eur. J. Org. Chem..

[21] Loro C., Molteni L., Lo Presti L., Foschi F., Beccalli E.M., Broggini G. (2022). Non-decarboxylative ruthenium-catalyzed rearrangement of 4-alkylidene-isoxazol-5-ones to pyrazole- and isoxazole-4-carboxylic acids. Org. Lett..

[22] Molteni L., Loro C., Christodoulou M.S., Papis M., Foschi F., Beccalli E.M., Broggini G. (2022). Ruthenium-catalyzed decarboxylative rearrangement of 4-alkenyl-isoxazol-5-ones to pyrrole derivatives. Eur. J. Org. Chem..

[23] Issazadeh S., Khan M.J., Gan H., Zhang J., Henderson L.C., Varley R.J. (2024). Synthesis and cure kinetics of bisoxazoline monomers during cationic ring-opening polymerization. J. Appl. Polym. Sci..

[24] Taylan E., Küsefoğlu S.H. (2012). Chain Extension Reactions of Unsaturated Polyesters with Bis(2-Oxazoline)s. J. Appl. Polym. Sci..

[25] Fry E.M. (1950). Oxazolidine ring-opening. J. Org. Chem..

[26] Tschan M., Thomas C.M., Strub H., Carpentier J.F. (2009). Copper(II) triflate as a source of triflic acid: Effective, green catalyst of hydroalkoxylation reactions. Adv. Synth. Catal..

[27] Kang Y.B., Gade L.H. (2011). The nature of the catalytically active species in olefin deoxygenation with PhI(OAc)_2_: Metal or proton?. J. Am. Chem. Soc..

[28] Dang T., Boeck F., Hinterman L. (2011). Hidden Brønsted acid catalysis: Pathways of accidental or delicerate generation of triflic acid from metal triflates. J. Org. Chem..

[29] Loro C., Oble J., Foschi F., Papis M., Beccalli E.M., Giofrè S., Poli G., Broggini G. (2022). Acid-mediated decarboxylative C–H coupling between arenes and *O*-allyl carbamates. Org. Chem. Front..

[30] Loro C., Papis M., Foschi F., Broggini G., Poli G., Oble J. (2023). Copper(II)-Catalyzed Three-Component Arylation/Hydroamination Cascade from Allyl Alcohol: Access to 1-Aryl-2-sulfonylamino-propanes. J. Org. Chem..

[31] Schwekendiek K., Glorius F. (2006). Efficient Oxidative Synthesis of 2-Oxazolines. Synthesis.

[32] Soleymani Movahed F., Foo S.W., Mori S., Ogawa S., Saito S. (2022). Phosphorus-Based Organocatalysis for the Dehydrative Cyclization of *N*-(2-Hydroxyethyl)amides into 2-Oxazolines. J. Org. Chem..

[33] Chemical Computing Group ULC (2023). Molecular Operating Environment (MOE).

[34] Halgren T.A. (1996). Merck molecular force field. I. Basis, form, scope, parameterization, and performance of MMFF94. J. Comput. Chem..

[35] Chai J.-D., Head-Gordon M. (2008). Long-range corrected hybrid density functionals with damped atom–atom dispersion corrections. Phys. Chem. Chem. Phys..

[36] Papis M., Bucci R., Contini A., Gelmi M.L., Lo Presti L., Poli G., Broggini G., Loro C. (2023). Phosphine-Catalyzed Domino Regio- and Stereo-Selective Hexamerization of 2-(Bromomethyl)acrylates to 1,2-Bis(cyclohexenyl)ethenyl Derivatives. Org. Lett..

[37] Weigend F., Ahlrichs R. (2005). Balanced basis sets of split valence, triple zeta valence and quadrupole zeta valence quality for H to Rn: Design and assessment of accuracy. Phys. Chem. Chem. Phys..

[38] Cossi M., Rega N., Scalmani G., Barone V. (2003). Energies, structures, and electronic properties of molecules in solution with the C-PCM solvation model. J. Comput. Chem..

[39] Frisch M.J., Trucks G.W., Schlegel H.B., Scuseria G.E., Robb M.A., Cheeseman J.R., Scalmani G., Barone V., Petersson G.A., Nakatsuji H. (2016). Gaussian 16, Revision A. 03.

[40] Sheldrick G.M. (2008). A short history of Shelx. Acta Crystallogr. Sect. A.

[41] Parsons S., Flack H.D., Wagner T. (2013). Use of intensity quotients and differences in absolute structure refinement. Acta Crystallogr. Sect. B.

