# Supplementary material for: Doubly Metathetic NiCl2-Catalyzed Coupling Between Bis(2-oxazolines) and Aldehydes: A Novel Access to Bis(ester-imine) Derivatives
Source: Molecules. 2024 Dec 5;29(23):5756. doi: 10.3390/molecules29235756 (PMC11643831; doi:10.3390/molecules29235756)

Supporting information

Doubly Metathetic $NiCl_2$ Catalyzed Coupling Between Bis(2-oxazolines) and Aldehydes: a Novel Access to Bis(Ester-Imine) Derivatives

Sara Colombo [1], Julie Oble [2], Giovanni Poli [2], Leonardo Lo Presti [3], Giovanni Macetti [3], Alessandro Contini [4], Gianluigi Broggini [1], Marta Papis [1,*] and Camilla Loro [1,*]

[1] Dipartimento di Scienza e Alta Tecnologia, Università degli Studi dell'Insubria, via Valleggio 9, 22100, Como, Italy

[2] Sorbonne Université, Faculté des Sciences et Ingénierie, CNRS, Institut Parisien de Chimie Moléculaire, IPCM, 4 place Jussieu, 75005 Paris, France

[3] Dipartimento di Chimica, Università degli Studi di Milano, via Golgi 19, 20133 Milano, Italy

[4] Dipartimento di Scienze Farmaceutiche, DISFARM Università degli Studi di Milano, Via Venezian 21, 20133, Milano, Italy

e-mail: camilla.loro@uninsubria.it; mpapis@uninsubria.it

Table of contents

General information S-2

Preparation of (*S*)-4-isopropyl-2-phenyl-4,5-dihydrooxazole **14** S-3

Computational methods S-4

Crystallographic data for compound **2d** S-17

^{1}H NMR spectra of known compounds S-20

^{1}H NMR and ^{13}C NMR spectra of unknown compounds S-21

General information

All available chemicals and solvents were purchased from commercial sources and were used without any further purification. Thin layer chromatography (TLC) was performed using 0.25 mm silica gel precoated plates Si 60-F254 (Merck, Darmstadt, Germany) visualized by UV-254 light and CAM staining. Purification by flash column chromatography (FCC) was conducted by using silica gel Si 60, 230-240 mesh, 0.040-0.063 mm (Merck). Melting points were determined on a Stuart Scientific SMP3 and are correct. ^{1}H and ^{13}C NMR spectra were recorded on a Bruker Avance 400 (400 and 101 MHz, respectively); chemical shifts are indicated in parts per million downfield from $SiMe_4$, using the residual proton ($CHCl_3$ = 7.26 ppm) and carbon ($CDCl_3$ = 77.0 ppm) solvent resonances as internal reference. Coupling constant values *J* are given in Hz. High-resolution mass spectra (HRMS) were recorded using a mass spectrometer from Thermo Fisher Scientific with an electron spray ion source (ESI) and a LTQ Orbitrap as detector. Optical rotations were measured on JASCO P-2000 polarimeter using a 100 mm path-length cell at 589 nm and reported as follows; $[\alpha]_D^{T\ °C}$ (c mg/10 mL, solvent).

Preparation of (*S*)-4-isopropyl-2-phenyl-4,5-dihydrooxazole 14

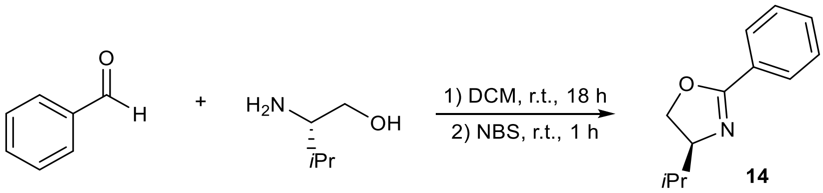

Starting material **14** was prepared according to literature procedure[1] described as follows. To a solution of the benzaldehyde (200 mg, 1.9 mmol, 1 equiv.) in DCM (8 mL), (*S*)-valinol (190 mg, 1.9 mmol, 1 equiv.) was added and the mixture was stirred at room temperature for 18 h.

NBS (270 mg, 1.9 mmol, 1 equiv.) was subsequently added and the mixture was stirred for 1 h at room temperature. The crude mixture was filtrated, the organic phase was washed with a saturated solution of Na_2CO_3 (10 mL), dried over Na_2SO_4 and concentrated to afford the mono-oxazoline **14** as a yellow liquid in 95% yield. ^{1}H NMR ($CDCl_3$, 400 MHz) δ 7.78 (d, 2H, *J* = 7.0 Hz), 7.53-7.49 (m, 1H), 7.45-7.41 (m, 2H), 3.98-3.91 (m, 1H), 3.84-3.71 (m, 2H), 2.07-1.98 (m, 1H), 1.04 (d, 3H, *J* = 6.8 Hz), 1.02 (d, 3H, *J* = 6.8 Hz). The characterization of **14** was consistent with that reported in literature.[1]

---

[1] Schwekendiek, K.; Glorius, F. Efficient Oxidative Synthesis of 2-Oxazolines. *Synthesis*, **2006**, *18*, 2996-3002

Computational methods

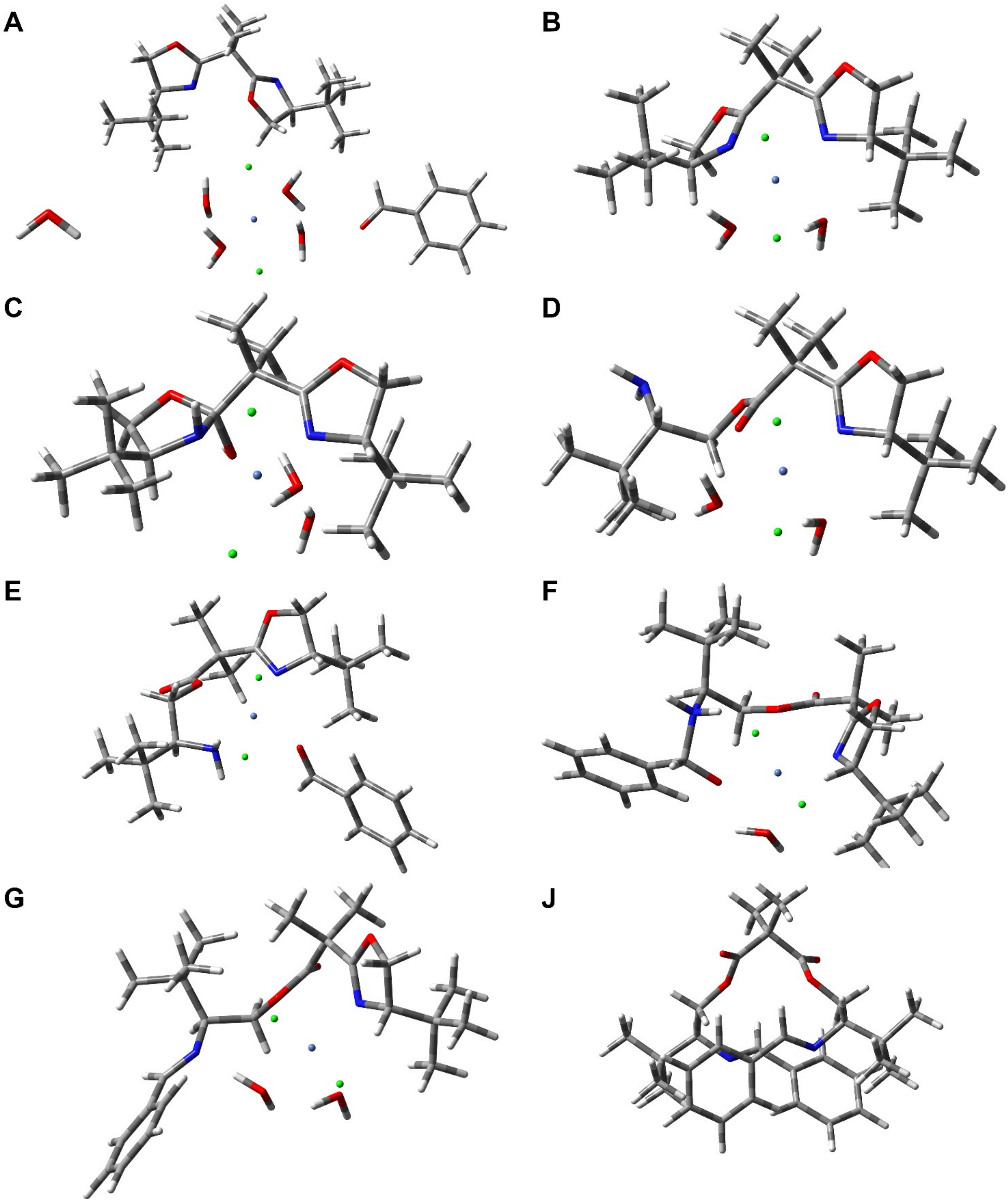

Figure S1. Enlarged picture of optimized geometries of reactants, intermediates and products discussed in Figure 1. A: catalyst $NiCl_2 \cdot 4H_2O$, reactants **1a**, benzaldehyde and water. B-G: intermediates II-VII, respectively. J: product **2b**

Table S1. Average distances and angles involving Ni atom. Angles involving Ni with O or N are generally measured between adjacent atoms, unless otherwise specified.

	**Distances (Å)**			**Angles (deg.)**				
**Compound**	Ni-O	Ni-Cl	Ni-N	O-Ni-O	O-Ni-Cl	Cl-Ni-Cl	N-Ni-O	N-Ni-Cl

**$NiCl_2{\cdot}4H_2O$**	2.12	2.32	//	90.0	90.0	180.0	//	//
**IntII[a]**	2.19	2.39	2.09	77.93	84.9	164.9	96.4	94.9
**IntIII[b]**	2.12	2.35	2.08	//	86.9	146.0	134.1[c]	99.2
**IntIV[d]**	2.13	2.38	2.07	87.7	87.9	167.5	92.7	96.1
**IntV [e]**	2.19	2.39	2.08	171.0	89.9	176.1	90.0	89.9
**IntVI**	2.18	2.42	2.05	84.5	90.5	174.7	95.8	88.6
**IntVII**	2.23[f]	2.35	2.06	88.0	88.4	170.6	92.0	94.6

a. Ni-O distance is the average distance between Ni and water oxygen; O-Ni-O is the angle between adjacent water oxygens and Ni. b. Only the Ni-coordinated water is considered in measures of distances and angles. c. The two N-Ni-O angles, involving water oxygen, Ni, and hemiaminal or 2-oxazoline nitrogens, measured 174.1 and 94.1 degrees respectively. d. O-Ni distances and angle were determined by considering the two water molecules and the ester sp^2 oxygen deriving from the opening of the first 2-oxazoline moiety. e. The O-Ni-O angle involves the sp^3 ester oxygen and the carbonyl oxygen of benzaldehyde, that occupy opposite vertices of the square bipyramid base. The N-Ni-N angle formed by Ni with the amino and the 2-oxazoline nitrogens, that also occupy opposite vertices, measures 161.8 deg. f. The distance between Ni and the ester sp^3 oxygen measures 2.48 Å, where distances between Ni and water oxygens are 2.10 Å averagely.

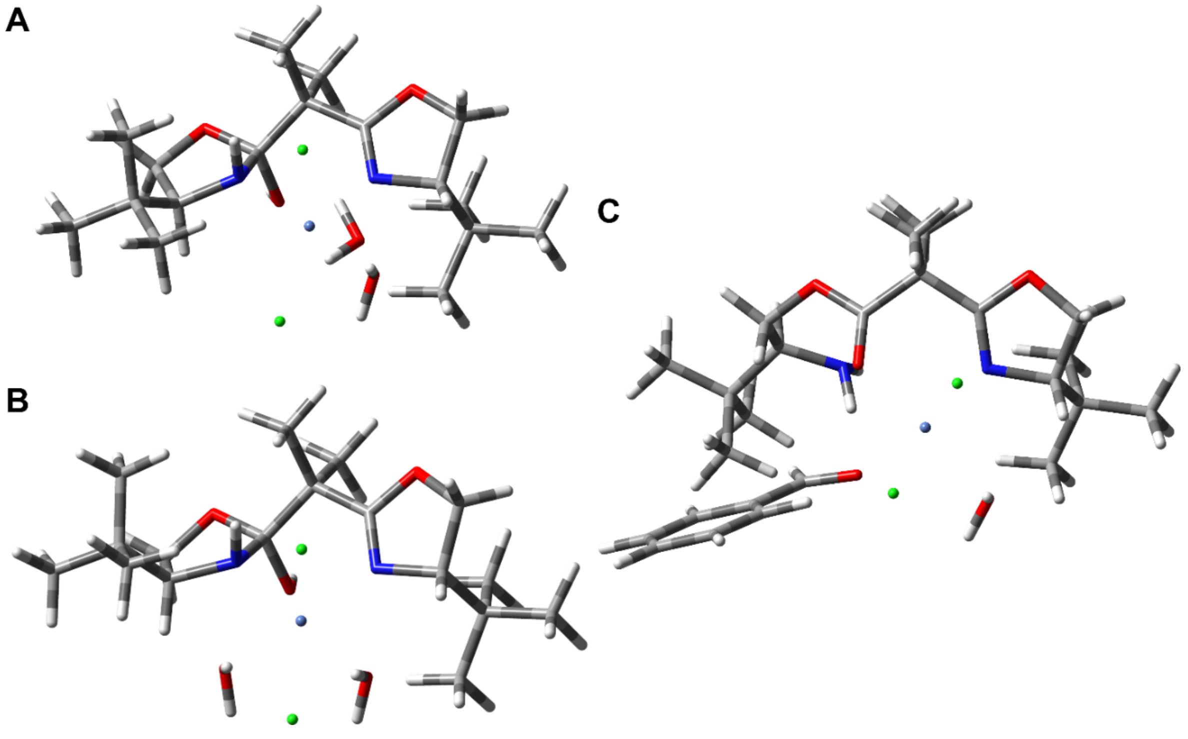

Figure S2. Alternative configurations for IntIII (A: pentavalent, and B: hexavalent; $\Delta H_{penta\text{-}hexa}$= 7.5 kcal/mol), and alternative structure for IntV (C) where the amine is free and replaced by a water molecule in coordinating Ni.

Table S2. Energies and corrections of each compound considered in this study

	E(SCF)	ZPEc	Hc	Gc	E(SCF)+ZPE	E(SCF) CPCM	E(SCF)+Hc	E(SCF)+Gc	E(SCF)+ZPEc	E(SCF)+Hc	E(SCF)+Gc
**PhCHO**	-345.472824	0.111002	0.118231	0.080442	-345.361822	-345.5890398	-345.354593	-345.392382	-345.4780378	-345.4708088	-345.5085978
**H_2O**	-76.409539	0.021747	0.025526	0.003459	-76.387792	-76.44494225	-76.384013	-76.40608	-76.42319525	-76.41941625	-76.44148325
**$NiCl_2 \cdot 4H_2O$**	-2734.38285	0.104647	0.120926	0.061536	-2734.2782	-2734.774942	-2734.261921	-2734.321311	-2734.670295	-2734.654016	-2734.713406
**Bisoxazoline 1a**	-925.635853	0.457696	0.481858	0.404847	-925.178157	-925.9240492	-925.153995	-925.231006	-925.4663532	-925.4421912	-925.5192022
**IntII**	-3507.21202	0.515738	0.549749	0.453203	-3506.696278	-3507.823008	-3506.662267	-3506.758813	-3507.30727	-3507.273259	-3507.369805
**IntIII penta**	-3583.6428	0.544508	0.579707	0.480241	-3583.098289	-3584.280617	-3583.06309	-3583.162556	-3583.736109	-3583.70091	-3583.800376
**IntIII hexa**	-3583.62736	0.544226	0.579289	0.481837	-3583.083133	-3584.268271	-3583.04807	-3583.145522	-3583.724045	-3583.688982	-3583.786434
**IntIV**	-3583.63478	0.542767	0.57947	0.476185	-3583.092011	-3584.275514	-3583.055308	-3583.158593	-3583.732747	-3583.696044	-3583.799329
**IntV, 0 H2O**	-3776.28209	0.605207	0.643773	0.534166	-3775.676885	-3776.977487	-3775.638319	-3775.747926	-3776.37228	-3776.333714	-3776.443321
**IntV, 1 H2O, free NH2**	-3852.69653	0.629229	0.67099	0.55355	-3852.067301	-3853.420151	-3852.02554	-3852.14298	-3852.790922	-3852.749161	-3852.866601
**IntVI**	-3852.69116	0.633637	0.673169	0.563241	-3852.057522	-3853.413412	-3852.01799	-3852.127918	-3852.779775	-3852.740243	-3852.850171
**IntVII**	-3852.69303	0.629886	0.670818	0.556837	-3852.063144	-3853.415442	-3852.022212	-3852.136193	-3852.785556	-3852.744624	-3852.858605
**Product 2b**	-1616.646437	0.683911	0.722688	0.612528	-1615.962526	-1617.155485	-1615.923749	-1616.033909	-1616.471574	-1616.432797	-1616.542957

Table S3. Energies and corrections of all the conformations of product **2b** obtained by a conformational search using molecular mechanics and the MMFF94x force field in gas phase, successively reoptimized at the WB97XD/Def2TZVP/CPCM(THF)//WB97XD/6-31+G(d,p) level

	E(SCF)	ZPEc	Hc	Gc	E(SCF)+ZPE	E+Hc	E+Gc	E(SCF)Sol	Esol+ZPE	Esol+Hc	Esol+Gc
**product2b_C0**	-1616.646437	0.683911	0.722688	0.612528	-1615.962526	-1615.923749	-1616.033909	-1617.1555	-1616.471574	-1616.4328	-1616.543
**product2b_C1**	-1616.647350	0.685393	0.724313	0.615914	-1615.961957	-1615.923037	-1616.031436	-1617.1562	-1616.470757	-1616.4318	-1616.5402
**product2b_C2**	-1616.634903	0.684481	0.723820	0.610325	-1615.950422	-1615.911083	-1616.024578	-1617.1458	-1616.461269	-1616.4219	-1616.5354
**product2b_C3**	-1616.636947	0.684170	0.723729	0.609428	-1615.952777	-1615.913218	-1616.027519	-1617.1491	-1616.464945	-1616.4254	-1616.5397
**product2b_C4**	-1616.637235	0.685048	0.724276	0.611426	-1615.952187	-1615.912959	-1616.025809	-1617.1467	-1616.461676	-1616.4224	-1616.5353
**product2b_C5**	-1616.636946	0.684169	0.723729	0.609343	-1615.952777	-1615.913217	-1616.027603	-1617.1491	-1616.464944	-1616.4254	-1616.5398
**product2b_C6**	-1616.637235	0.685049	0.724276	0.611428	-1615.952186	-1615.912959	-1616.025807	-1617.1467	-1616.461675	-1616.4224	-1616.5353
**product2b_C7**	-1616.637235	0.685048	0.724275	0.611423	-1615.952187	-1615.912960	-1616.025812	-1617.1467	-1616.461676	-1616.4224	-1616.5353
**product2b_C8**	-1616.640298	0.684895	0.724019	0.612846	-1615.955403	-1615.916279	-1616.027452	-1617.1512	-1616.466275	-1616.4272	-1616.5383
**product2b_C9**	Not converged										
**product2b_C10**	-1616.634613	0.684872	0.723921	0.613888	-1615.949741	-1615.910692	-1616.020725	-1617.145	-1616.460130	-1616.4211	-1616.5311
**product2b_C11**	-1616.637826	0.684112	0.723732	0.609690	-1615.953714	-1615.914094	-1616.028136	-1617.1484	-1616.464256	-1616.4246	-1616.5387

**product2b_C12**	-1616.640299	0.684902	0.724023	0.612862	-1615.955397	-1615.916276	-1616.027437	-1617.1512	-1616.466265	-1616.4271	-1616.5383
**product2b_C13**	-1616.639063	0.685190	0.724109	0.613936	-1615.953873	-1615.914954	-1616.025127	-1617.1508	-1616.465636	-1616.4267	-1616.5369
**product2b_C14**	-1616.647350	0.685400	0.724316	0.615937	-1615.961950	-1615.923034	-1616.031413	-1617.1561	-1616.470748	-1616.4318	-1616.5402
**product2b_C15**	-1616.642077	0.684822	0.724285	0.611056	-1615.957255	-1615.917792	-1616.031021	-1617.1498	-1616.465001	-1616.4255	-1616.5388
**product2b_C16**	-1616.635880	0.685304	0.724423	0.612539	-1615.950576	-1615.911457	-1616.023341	-1617.1465	-1616.461216	-1616.4221	-1616.534
**product2b_C17**	-1616.637920	0.685040	0.724258	0.611810	-1615.952880	-1615.913662	-1616.026110	-1617.1486	-1616.463574	-1616.4244	-1616.5368
**product2b_C18**	-1616.632501	0.685185	0.724282	0.612683	-1615.947316	-1615.908219	-1616.019818	-1617.1447	-1616.459471	-1616.4204	-1616.532
**product2b_C19**	-1616.636287	0.684322	0.723820	0.609190	-1615.951965	-1615.912467	-1616.027097	-1617.1476	-1616.463327	-1616.4238	-1616.5385
**product2b_C20**	-1616.629890	0.684409	0.723924	0.609113	-1615.945481	-1615.905966	-1616.020777	-1617.1436	-1616.459233	-1616.4197	-1616.5345

Coordinates of all the optimized geometries in Gaussian16 input format.

All the stationary points were confirmed as minima by vibrational analysis, and 0 imaginary frequencies were found.

Single point energy calculations were all performed on optimized geometries by using the following root:

wb97xd/def2tzvp scrf=(cpcm,solvent=thf) geom=allcheck guess=tcheck 10f 6d gfinput gfprint

%chk=benzaldehyde.chk

opt freq wb97xd/6-31+g(d,p) 10f 6d gfinput gfprint

PhCHO

0 1

O	-2.84299900	-0.39457700	-0.00053300
C	-1.99034400	0.46721500	0.00042000
H	-2.27598900	1.54023300	0.00146400
C	-0.53159000	0.20839200	0.00013700
C	-0.04186400	-1.10222100	0.00015000
C	0.35582500	1.28702500	-0.00005900
C	1.32875700	-1.32615000	0.00011100
H	-0.74947700	-1.92526700	0.00022000
C	1.72961600	1.06156700	-0.00019200
H	-0.03159100	2.30317400	-0.00008900
C	2.21353500	-0.24505700	-0.00009600
H	1.71326500	-2.34096900	0.00027600
H	2.41983600	1.89884300	-0.00033500
H	3.28432900	-0.42402800	-0.00009000

%chk=h2o.chk

opt freq wb97xd/6-31+g(d,p) 10f 6d gfinput gfprint

H2O

0 1

O	0.00000000	0.11571500	0.00000000
H	0.76635500	-0.46284600	0.00000000
H	-0.76635500	-0.46287700	0.00000000

%chk=nicl2_4h2o_triplet.chk

opt freq uwb97xd/6-31+g(d,p) 10f 6d gfinput gfprint

NiCl2-4H2O triplet

0 3

Ni	0.00060400	-0.00004300	0.00018000
Cl	2.32244600	-0.00239800	0.00228000
Cl	-2.32197400	0.00289200	-0.00232500
O	-0.00236700	-2.00917000	-0.66899000
H	-0.78582300	-2.12297000	-1.22106200
O	0.00352600	0.66846600	-2.00931000
H	-0.77812700	1.22256300	-2.12553700
H	0.78003900	-2.12662300	-1.22173000
H	0.78777700	1.21916500	-2.12425900
O	0.00371900	2.00938200	0.66882800
O	-0.00670700	-0.66945200	2.00911500
H	0.77080900	-1.22878400	2.12742400
H	-0.79508900	-1.21486200	2.12144400
H	0.78802700	2.12359300	1.21963700
H	-0.77789500	2.12691900	1.22266400

%chk=bisoxazoline.chk

opt freq wb97xd/6-31+g(d,p) 10f 6d gfinput gfprint

Bisoxazoline **1a**

0 1

C	-1.36126100	1.16563200	-0.19748500
C	0.03026800	1.67973900	0.08874400

C	0.38474800	2.74964900	-0.95647800
C	0.10560500	2.27109400	1.51056600
O	-2.37626600	1.95244400	0.24091700
C	-3.58522000	1.33014900	-0.22877100
C	-3.11117200	-0.02360900	-0.81495000
C	-3.56203600	-1.28969700	-0.03962200
C	-2.96356400	-2.52079200	-0.73524900
C	-5.09380900	-1.38493000	-0.08134500
C	-3.07315000	-1.24449300	1.41469100
N	-1.65014800	0.09837100	-0.81975100
C	2.62009600	-0.87112800	-0.50849100
C	4.09698400	-0.61310400	-0.11171000
C	4.81059700	0.05056000	-1.29814800
C	4.77530900	-1.95680200	0.19233000
C	4.18502500	0.30784700	1.11263900
N	1.92479500	0.37677300	-0.84526400
C	0.98067300	0.51270400	-0.00866200
O	0.81030200	-0.44797100	0.93424100
C	1.72505500	-1.49844500	0.58907600
H	1.38810500	3.13774500	-0.76479700
H	-0.33290700	3.57238800	-0.89606100
H	0.37172300	2.32760000	-1.96310900
H	1.13256200	2.58626500	1.71756700
H	-0.19075900	1.53734500	2.26307900
H	-0.55093900	3.13983800	1.58899200
H	-4.26738000	1.24428300	0.61949400
H	-4.03175600	1.98437400	-0.98244600
H	-3.45892000	-0.13341000	-1.84930200
H	-1.87174200	-2.47392500	-0.73223300
H	-3.29673600	-2.58490000	-1.77760500
H	-3.27814100	-3.43730300	-0.22396900
H	-5.42962800	-2.30606700	0.40649500
H	-5.46075300	-1.40148200	-1.11434300
H	-5.57609800	-0.54848200	0.43657800
H	-3.48411400	-0.38799800	1.96122500
H	-1.98128400	-1.18459600	1.45689900
H	-3.38668700	-2.15025200	1.94463600
H	4.34084800	1.00434000	-1.55088500
H	4.77268800	-0.59003600	-2.18675400
H	5.86424300	0.23025300	-1.05718400
H	4.33166800	-2.45903200	1.05907100
H	5.83726500	-1.80446400	0.41241600
H	4.70621700	-2.63615500	-0.66525300
H	5.23277400	0.48482000	1.37778600
H	3.69192800	-0.12433000	1.99047000
H	3.72113500	1.27677400	0.90376200
H	2.63055300	-1.51064800	-1.39912700
H	2.25514600	-1.79495700	1.49662000
H	1.14129300	-2.34602600	0.21901100

%chk=int_II.chk

opt freq uwb97xd/6-31+g(d,p) 10f 6d gfinput gfprint

IntII

0 3

C	1.23150800	1.47482400	0.45430700
C	-0.00783500	2.28128600	0.16867400
C	-0.60462400	2.77371200	1.50999300
C	0.40402700	3.47952600	-0.71032200
O	2.17179600	2.14967900	1.12334100
C	3.17442500	1.17107400	1.48182000
C	2.90086500	0.00707700	0.51883400
C	3.84947100	-0.06388100	-0.70949600
C	3.42720700	-1.22348300	-1.61975300
C	5.27498200	-0.33453200	-0.20174300
C	3.82284500	1.24334700	-1.51485600
N	1.49566400	0.26810900	0.12565500
C	-2.69827700	-0.00416700	-0.93404900
C	-3.93459300	0.04663300	0.00782000
C	-3.78909400	-1.00438200	1.11521600
C	-5.18494300	-0.28352400	-0.82279100
C	-4.09544600	1.42495400	0.66546800
N	-1.43215100	0.25879100	-0.21492000
C	-1.07205600	1.45146700	-0.51004100
O	-1.78487100	2.08696100	-1.44747000
C	-2.66919300	1.08853700	-2.00897100
H	-1.51791000	3.34291500	1.31534300
H	0.11373400	3.42560300	2.01074000
H	-0.83066400	1.92380600	2.15962000
H	-0.45862600	4.12273000	-0.89058500
H	0.79532900	3.14823500	-1.67559600
H	1.17509200	4.05607600	-0.19659100
H	4.14886300	1.64534100	1.37104000
H	3.00166600	0.89545400	2.52504500
H	2.93600200	-0.94983900	1.04266300
H	2.39936900	-1.11105800	-1.97353800
H	3.50068000	-2.17799300	-1.08929700
H	4.08731200	-1.27094100	-2.49273400
H	5.94947000	-0.48557600	-1.05073300
H	5.30708500	-1.24006600	0.41448100
H	5.67956200	0.49448900	0.38885200
H	4.11419200	2.11165500	-0.91336600
H	2.82638300	1.42787300	-1.92841100
H	4.52077300	1.17625400	-2.35553100
H	-2.87445500	-0.84478100	1.69642600
H	-3.78688400	-2.01416200	0.68678600
H	-4.64102000	-0.94861600	1.80067100
H	-5.39744200	0.47730500	-1.58099700
H	-6.06222300	-0.34394500	-0.17074600
H	-5.07932800	-1.24807700	-1.33225100
H	-4.99952400	1.43801700	1.28248500
H	-4.18818900	2.23115400	-0.06970200
H	-3.24792100	1.64714900	1.31956700

H	-2.63435000	-0.99283400	-1.39621400
H	-3.62430800	1.57206900	-2.21019000
H	-2.21780700	0.73612900	-2.93988300
Ni	0.01207400	-1.18240800	0.25848000
Cl	-0.16166000	-2.00864800	-2.00698800
Cl	-0.07837000	-0.92166000	2.60319600
O	1.50732100	-2.75332500	0.46667200
H	1.08987500	-3.30404600	1.14001000
O	-1.23336800	-2.95558300	0.63566500
H	-1.84400000	-2.78705300	1.36157800
H	1.31979600	-3.16857400	-0.39023400
H	-1.72528900	-3.14298800	-0.17318800

%chk=int_III.chk

opt freq uwb97xd/6-31+g(d,p) 10f 6d gfinput gfprint

IntIII

0 3

C	1.33705700	1.38762300	0.80070700
C	0.14063200	2.30679600	0.77517100
C	-0.50545600	2.32747400	2.17564200
C	0.62949900	3.72571500	0.41766300
O	2.37302400	1.84237800	1.51609500
C	3.31647800	0.74895700	1.58680800
C	2.89604200	-0.16206000	0.42651600
C	3.72849200	0.01208700	-0.87558400
C	3.13458500	-0.85647500	-1.99149500
C	5.16258000	-0.46192700	-0.59181200
C	3.74153700	1.47583900	-1.34147000
N	1.48725100	0.24761900	0.23597500
C	-2.72000700	0.52415600	-0.80462300
C	-3.83776500	-0.32514500	-0.15417200
C	-3.45433100	-1.81270800	-0.16901400
C	-5.10306200	-0.14475700	-1.01110300
C	-4.12411800	0.10261800	1.29491500
N	-1.43823800	0.52178200	-0.04936400
C	-0.85740100	1.83598500	-0.31367500
O	-1.93572300	2.72743600	-0.34988400
C	-3.04316700	2.03591900	-0.93304200
H	-1.42680900	2.91332900	2.14031900
H	0.18021600	2.79442800	2.88601400
H	-0.72272100	1.32201300	2.54739200
H	-0.22866900	4.39732800	0.34846800
H	1.17543000	3.73979100	-0.52996700
H	1.30359400	4.09266100	1.19274700
H	4.31672900	1.17224000	1.50401500
H	3.19136200	0.26971800	2.56152700
H	2.91658600	-1.21472700	0.71599700
H	2.10871800	-0.56650300	-2.23199800
H	3.12913000	-1.91170700	-1.70427900
H	3.73734900	-0.75387200	-2.90031700
H	5.75684500	-0.42016300	-1.51016000
H	5.17081200	-1.49845400	-0.23663200
H	5.67398400	0.15804700	0.15272100
H	4.16549600	2.15286900	-0.59158700
H	2.72919500	1.81394800	-1.58517000
H	4.34780400	1.56849000	-2.24819900
H	-2.59910400	-2.01378700	0.48302800
H	-3.20210500	-2.14876700	-1.17846000
H	-4.29352300	-2.41207000	0.20114800
H	-5.48383200	0.88195700	-0.98648600
H	-5.90040300	-0.79654700	-0.64056100
H	-4.91004500	-0.41429800	-2.05527200
H	-5.00084600	-0.43607400	1.66812100
H	-4.33601300	1.17359300	1.38555600
H	-3.29570200	-0.14737700	1.96741300
H	-2.49979000	0.10901900	-1.79060900
H	-3.93156800	2.34467200	-0.37938400
H	-3.15913900	2.32910400	-1.98056500
Ni	-0.02606800	-1.05085200	-0.16356500
Cl	-0.51144700	-1.67816000	-2.31302700
Cl	-0.47037700	-1.42205900	2.17077000
O	1.23812500	-2.75596200	-0.19027600
H	1.01750800	-3.44812100	0.47420300
O	0.52090900	-4.34024000	1.88407400
H	0.12569500	-3.53848300	2.27768200
H	1.01820800	-3.09981600	-1.06801500
H	-0.18710900	-4.98634000	1.82246300
H	-1.65508900	0.48134300	0.94604600
O	-0.25951600	1.76059800	-1.57836200
H	-0.29850300	2.64008600	-1.96705700

%chk=int_III_hexa.chk

opt freq uwb97xd/6-31+g(d,p) 10f 6d gfinput gfprint

IntIII hexavalent

0 3

C	1.22614800	1.47948000	0.50690100
C	0.01151200	2.28085700	0.10789600
C	-0.73046200	2.72747100	1.38289900
C	0.48977800	3.52868500	-0.66352200
O	2.10995600	2.16662700	1.24502500
C	3.06602800	1.19091100	1.71499600

C	2.88769400	0.02271400	0.73844100
C	3.92586500	-0.01911200	-0.41916900
C	3.58243400	-1.15887300	-1.38475400
C	5.31214600	-0.28844500	0.18772200
C	3.94766500	1.30190700	-1.20299300
N	1.51224000	0.26036600	0.24111700
C	-2.78495100	0.01021600	-0.76850400
C	-3.95525100	-0.05134600	0.25565900
C	-3.76369000	-1.19598300	1.25999900
C	-5.24737800	-0.33210300	-0.53297700
C	-4.11130300	1.26337900	1.03830300
N	-1.43549100	0.23547800	-0.15683200
C	-0.88225000	1.42489500	-0.82056700
O	-1.98574000	2.17331000	-1.24232900
C	-2.90557000	1.20326000	-1.74410800
H	-1.68200100	3.18847100	1.10973900
H	-0.12398300	3.45920800	1.91994000
H	-0.90929400	1.89509500	2.07088900
H	-0.37666700	4.10647500	-0.99299400
H	1.09645600	3.26241000	-1.53378300
H	1.10478300	4.15259700	-0.01315500
H	4.04894700	1.66053800	1.70292100
H	2.78645800	0.92236900	2.73722900
H	2.90657300	-0.93461000	1.26177800
H	2.58470400	-1.04171100	-1.81399400
H	3.61857700	-2.12605300	-0.87459100
H	4.30873500	-1.18273800	-2.20465900
H	6.05061200	-0.41069200	-0.61124500
H	5.30868800	-1.20959000	0.78146300
H	5.66106200	0.52858000	0.82856900
H	4.19226600	2.16281700	-0.57128400
H	2.97969400	1.48441900	-1.68044600
H	4.70167300	1.24916000	-1.99495800
H	-2.83467200	-1.10394900	1.83084000
H	-3.74722700	-2.16115900	0.74902700
H	-4.59560200	-1.19430400	1.97280700
H	-5.53248100	0.49469700	-1.19123900
H	-6.07702700	-0.49251800	0.16272700
H	-5.14645900	-1.23543500	-1.14453800
H	-5.01681900	1.21512300	1.65136400
H	-4.19437200	2.13861400	0.38704400
H	-3.27471900	1.43169200	1.72316300
H	-2.74701300	-0.93309300	-1.31884600
H	-3.88832200	1.67157700	-1.75739400
H	-2.63040800	0.91109700	-2.76168100
Ni	0.05623200	-1.25456300	0.11969100
Cl	0.17865700	-2.26589800	-2.07352500
Cl	-0.18119300	-1.09821900	2.50670500
O	1.56708000	-2.76621700	0.52997000
H	1.30208600	-3.28827500	1.29460900
O	-1.44831000	-2.82283700	0.22899200
H	-1.19018000	-3.35704700	-0.53934800
H	1.48262600	-3.29532600	-0.27898000
H	-1.42879100	-3.34482200	1.03741500
H	-1.56653700	0.45477400	0.82936600
O	-0.18157400	0.96793900	-1.94101500
H	-0.14178700	1.69040000	-2.57561000

%chk=int_IV.chk

opt freq uwb97xd/6-31+g(d,p) 10f 6d gfinput gfprint

IntIV

0 3

C	-1.64170700	-1.48248200	0.47078900
C	-0.31972200	-2.19694200	0.62398000
C	-0.08622800	-2.48981000	2.12697600
C	-0.36675800	-3.50266000	-0.18666300
O	-2.69861100	-2.21090700	0.84103300
C	-3.82260900	-1.30039500	0.84902000
C	-3.33679300	-0.11647000	-0.00226400
C	-3.88273600	-0.08274400	-1.45550200
C	-3.27498400	1.10962300	-2.20406100
C	-5.40737500	0.10352800	-1.39230200
C	-3.54490600	-1.37766700	-2.20816700
N	-1.86803100	-0.29733400	0.04337000
C	3.87096500	-0.89749900	-0.14552500
C	4.79281600	0.33494800	-0.42539100
C	6.03038100	0.25264600	0.47979400
C	4.03204500	1.62811900	-0.08251900
C	5.24435700	0.37839100	-1.89305500
N	4.51312100	-2.20744100	-0.10333200
C	0.81233100	-1.27770500	0.18427600
O	1.64926700	-1.82034500	-0.66521200
C	2.72113800	-0.97394200	-1.14834500
H	0.88948400	-2.96718300	2.25700900
H	-0.85947600	-3.17134700	2.48675100
H	-0.12363500	-1.56585900	2.70937300
H	0.57454600	-4.04314400	-0.07293800
H	-0.52488600	-3.30742800	-1.25018500
H	-1.18253000	-4.12769500	0.18093200
H	-4.68067900	-1.83779700	0.44707200
H	-4.00461500	-1.01721500	1.88847600
H	-3.57583400	0.83333900	0.48297800
H	-2.18569200	1.04297100	-2.26073600
H	-3.52912500	2.05060100	-1.70598800
H	-3.66973100	1.14781500	-3.22525300
H	-5.80926900	0.22105600	-2.40374900
H	-5.67044100	1.00232400	-0.82320700
H	-5.92114700	-0.75038100	-0.93765300
H	-3.95786000	-2.26657600	-1.71806200

H	-2.46174800	-1.50506300	-2.30099400
H	-3.95966300	-1.33814500	-3.22042300
H	6.68009000	-0.58560100	0.20860400
H	5.74566300	0.14024700	1.53310000
H	6.62407900	1.16832200	0.39188200
H	3.14050400	1.76521200	-0.70363800
H	4.67841600	2.49883800	-0.23538500
H	3.72667500	1.61796000	0.97148700
H	5.96411200	1.18955700	-2.04407000
H	4.40600900	0.55593500	-2.57437000
H	5.73993700	-0.55310500	-2.19197200
H	3.43474900	-0.73372600	0.84683700
H	3.04051500	-1.46688700	-2.06812600
H	2.28909800	-0.00046300	-1.38423000
Ni	-0.59021500	1.26732900	0.50760800
Cl	0.28609400	1.86600400	-1.62203800
Cl	-1.26596300	1.12638200	2.77553100
O	-1.97436200	2.84815700	0.22731200
H	-1.89949400	3.36734600	1.03758700
O	0.76341400	2.75463100	1.27517900
H	0.91294400	2.45338700	2.18148500
H	-1.52227700	3.33113700	-0.48052500
H	1.58471200	2.67657000	0.77483600
H	5.04382000	-2.39565800	-0.94836800
O	0.96835800	-0.14852900	0.63652500
H	5.14506900	-2.28937100	0.68384100

%chk=int_V.chk

opt freq uwb97xd/6-31+g(d,p) 10f 6d gfinput gfprint

IntV

0 3

C	-1.11161200	2.57749300	0.12593300
C	-2.35366300	2.39185600	-0.72698800
C	-3.48688900	3.32277700	-0.23869900
C	-1.98696300	2.73605400	-2.18465300
O	-1.06626100	3.72824800	0.81164600
C	0.12187400	3.65492100	1.63313000
C	0.96028600	2.56903500	0.95056700
C	2.08359000	3.10200100	0.01813900
C	2.75116700	1.92490800	-0.70244000
C	3.13573300	3.80673400	0.88869400
C	1.53549000	4.07824800	-1.03285200
N	-0.07524900	1.82649900	0.20167900
C	-2.49055500	-2.14117800	-0.04576000
C	-3.23539200	-3.49234200	0.14478700
C	-2.41642800	-4.60812100	-0.52441100
C	-4.60255700	-3.41381600	-0.55560400
C	-3.43669500	-3.83221000	1.62958000
N	-1.06638400	-2.15713700	0.34261500
C	-2.95098700	0.98310900	-0.66860000
O	-2.47693900	0.25677900	0.36732600
C	-3.14606800	-0.98058000	0.69545400
H	-4.36349000	3.17087000	-0.87138700
H	-3.17086300	4.36450700	-0.31098500
H	-3.75651400	3.11820000	0.80096900
H	-2.87339900	2.62094300	-2.81209800
H	-1.20455600	2.06898500	-2.55183000
H	-1.64728100	3.77467800	-2.23783700
H	0.56957600	4.64811100	1.65183500
H	-0.19155000	3.35940000	2.63761800
H	1.39593300	1.89289900	1.69149900
H	2.02782800	1.38115900	-1.31840900
H	3.19330700	1.23207100	0.02049600
H	3.55304200	2.28970600	-1.35354800
H	3.97913100	4.12977200	0.26965400
H	3.52446500	3.13239200	1.65996700
H	2.73877700	4.69804400	1.38629400
H	1.04599700	4.94750300	-0.58079100
H	0.81781600	3.58119900	-1.69230200
H	2.35455700	4.45021300	-1.65700300
H	-1.48279100	-4.80973600	0.01133600
H	-2.17273600	-4.35332900	-1.56236300
H	-2.98935900	-5.54076400	-0.53353500
H	-5.26790200	-2.68086300	-0.09019700
H	-5.10506500	-4.38537100	-0.50608400
H	-4.49063500	-3.14298800	-1.61060400
H	-3.87481200	-4.83072600	1.72883500
H	-4.11396900	-3.12776700	2.12283500
H	-2.49202800	-3.83470900	2.18500500
H	-2.49892600	-1.89877300	-1.11427900
H	-3.00939000	-1.06193800	1.77545200
H	-4.20020200	-0.88091500	0.44043200
Ni	-0.25098200	-0.24188400	0.24837400
Cl	-0.31113600	-0.44254900	-2.13827300
Cl	-0.27854700	-0.17685000	2.62393100
O	1.72061100	-0.95689400	0.33059500
H	-0.93372400	-2.46970900	1.30346600
O	-3.84292600	0.61126400	-1.38584400
H	-0.54282400	-2.76449100	-0.27997700
C	2.42836500	-1.31929400	-0.60331400
H	2.02222300	-1.30084500	-1.62756800
C	3.81235800	-1.75996600	-0.40873200
C	4.37554600	-1.79459200	0.87385700
C	4.56988700	-2.12881000	-1.52467600
C	5.69331800	-2.19970900	1.03155500
H	3.76572700	-1.50158400	1.72305600
C	5.89111900	-2.53335400	-1.36207300
H	4.12210400	-2.09300700	-2.51439900
C	6.44868400	-2.56797600	-0.08518400
H	6.13783300	-2.23016100	2.02083900
H	6.48409400	-2.81913000	-2.22441200

H	7.47961400	-2.88317600	0.04324400

%chk=IntV_h2o_free-nh2.chk
opt freq uwb97xd/6-31+g(d,p) 10f 6d gfinput gfprint

IntV with 1h2o and free amine.

0 3

C	2.60567600	-0.04429100	-1.23161200
C	1.76538500	-0.90989300	-2.14201100
C	1.63387000	-0.20594700	-3.51758200
C	2.47614800	-2.27050600	-2.28571600
O	3.82157600	0.20468200	-1.73356400
C	4.40473900	1.21460000	-0.88331300
C	3.55483300	1.14706500	0.39455600
C	4.21916300	0.41329700	1.58966400
C	3.24956200	0.39509000	2.77613400
C	5.47826600	1.19511700	1.99797300
C	4.59407300	-1.02818500	1.21754400
N	2.33492900	0.45871900	-0.08653200
C	-0.96278000	-3.49516100	-0.19726000
C	-2.18592600	-3.89825800	0.68888300
C	-1.67619300	-4.76620900	1.84904100
C	-3.17787300	-4.72652900	-0.14501600
C	-2.90629300	-2.67131000	1.27041700
N	0.14542100	-2.84004200	0.47869900
C	0.35066200	-1.09137900	-1.61895000
O	-0.22297800	-2.18495300	-2.09366400
C	-1.39493200	-2.63730300	-1.40246300
H	1.00623300	-0.81111500	-4.17686800
H	2.62366100	-0.10709300	-3.96681100
H	1.19660500	0.78921000	-3.39964400
H	1.96139800	-2.88035200	-3.02923900
H	2.47070400	-2.80553100	-1.33274200
H	3.50614600	-2.11083300	-2.60967400
H	5.45687500	0.96375000	-0.75215300
H	4.30318400	2.17300400	-1.39812400
H	3.26837800	2.15014600	0.72033000
H	2.33323600	-0.15070500	2.54364800
H	2.96103900	1.41243500	3.06088800
H	3.72599400	-0.07891200	3.64165500
H	5.91649400	0.74969600	2.89697800
H	5.23502600	2.23859400	2.22848800
H	6.25287800	1.18885000	1.22357500
H	5.29545500	-1.07245700	0.37644400
H	3.70260000	-1.60640200	0.95405900
H	5.07157100	-1.52150700	2.07049700
H	-1.04532000	-4.18915100	2.53171100
H	-1.09680300	-5.62164000	1.48052600
H	-2.51799700	-5.15838300	2.42929300
H	-3.65141800	-4.13361800	-0.93469000
H	-3.98049800	-5.11161200	0.49283800
H	-2.68416300	-5.58625500	-0.61349500
H	-3.67013200	-2.99311600	1.98677800
H	-3.41889200	-2.09678200	0.49018000
H	-2.21755500	-2.00088200	1.79547900
H	-0.55684900	-4.42898400	-0.61136500
H	-1.98814200	-1.77188600	-1.10406500
H	-1.94791800	-3.22458900	-2.13421600
Ni	0.43619100	1.22837900	0.25718700
Cl	-0.25216300	0.01253100	2.21707200
Cl	0.89322400	2.84289600	-1.43152300
O	1.00419600	2.77340800	1.60146600
H	0.74858000	3.55742400	1.09499000
O	-1.56071300	2.05171600	0.24971900
H	0.40376500	2.67047400	2.35084300
H	-0.12593700	-2.03290800	1.04233700
O	-0.27214000	-0.27744500	-0.95082100
H	0.64465700	-3.48471200	1.07795700
C	-2.54222700	1.32841100	0.35365400
H	-2.41214400	0.27055800	0.63075300
C	-3.91328200	1.80449700	0.13286800
C	-4.15347300	3.09691700	-0.34931800
H	-3.30773900	3.74170900	-0.56632600
C	-4.97991000	0.94346100	0.40769300
H	-4.78009200	-0.05625400	0.78515300
C	-5.45982800	3.52087800	-0.55100300
H	-5.65457500	4.51896200	-0.92942900
C	-6.28810100	1.37386600	0.21009900
H	-7.11927700	0.71143500	0.42793100
C	-6.52454400	2.66094200	-0.26967000
H	-7.54452000	2.99755000	-0.42798200

%chk=int_VI.chk

opt freq uwb97xd/6-31+g(d,p) 10f 6d gfinput gfprint

IntVI

0 3

C	-2.58528000	1.12694300	0.00661500
C	-2.02409700	2.06337800	1.05374500
C	-2.06075400	3.52354800	0.54625100
C	-2.93069600	1.91025300	2.29621200
O	-3.52246300	1.68192600	-0.77487200
C	-3.89637900	0.66518600	-1.73431300
C	-3.40998600	-0.63518800	-1.08496300
C	-4.51111900	-1.46119000	-0.36484400
C	-3.86923400	-2.64411200	0.37038000
C	-5.46920300	-2.00387000	-1.43712800
C	-5.29015600	-0.61237000	0.64991000
N	-2.38612600	-0.12799200	-0.14412500

C	2.55487000	1.22235900	-0.15099700
C	2.64529000	2.63563900	-0.80456300
C	3.95991100	2.72617800	-1.60244800
C	2.68449400	3.70088800	0.30188600
C	1.46350300	2.92673800	-1.74498600
N	2.31421800	0.19096400	-1.19961900
C	-0.58130500	1.82990300	1.50350500
O	0.22294300	1.20638800	0.60478800
C	1.60279400	1.05912700	1.02256000
H	-1.65618800	4.17997200	1.31950100
H	-3.08931200	3.81764000	0.33372900
H	-1.47622500	3.64617500	-0.36996400
H	-2.62565100	2.63091200	3.05655700
H	-2.83753400	0.90227600	2.70814800
H	-3.97045300	2.10064800	2.01710600
H	-4.97349500	0.73548900	-1.88249700
H	-3.36775000	0.88263000	-2.66560800
H	-2.91367100	-1.27373900	-1.81984300
H	-3.19325300	-2.29725400	1.15838000
H	-3.30476800	-3.27499200	-0.32312400
H	-4.64747300	-3.25870000	0.83572800
H	-6.22898400	-2.63996000	-0.97169500
H	-4.93165600	-2.60969300	-2.17492400
H	-5.99709100	-1.20624000	-1.97152500
H	-5.77980200	0.25193100	0.18786300
H	-4.63231600	-0.25378600	1.44651600
H	-6.07112000	-1.22010300	1.11812300
H	4.00558900	2.01438700	-2.43593400
H	4.83022300	2.55183900	-0.96077500
H	4.06003200	3.72166400	-2.04523800
H	1.72637000	3.78777400	0.82240800
H	2.91009000	4.67699600	-0.13786400
H	3.45962600	3.48442700	1.04578700
H	1.52417300	3.96235800	-2.09430100
H	0.50466800	2.78635600	-1.24292100
H	1.46057900	2.28486300	-2.63188900
H	3.54097600	0.99385700	0.27303600
H	1.82147600	1.80836400	1.77944900
H	1.68933600	0.06841700	1.46797900
Ni	-0.53768700	-0.99286100	-0.00428400
Cl	-0.73102600	-1.35015000	2.31076200
Cl	-0.36852100	-0.39053600	-2.40984500
O	-0.82904200	-3.07205900	-0.27920700
H	0.14095000	-3.15360800	-0.33458100
O	1.36975300	-1.63931900	0.01096500
H	-1.03653400	-3.29281700	0.64186900
H	1.35988100	0.27089300	-1.62951200
O	-0.16447700	2.30025700	2.53007500
H	2.97711700	0.34008800	-1.96004100
C	2.37390700	-1.33915600	-0.79925500
H	2.31121000	-1.79574400	-1.80646900
C	3.75503600	-1.57898800	-0.20489000
C	3.88103600	-1.86975300	1.15178200
C	4.89889600	-1.48747400	-1.00057200
C	5.14493600	-2.04560800	1.71218600
H	2.97565700	-1.96770600	1.74366700
C	6.16250600	-1.65919400	-0.44148200
H	4.80700400	-1.29796900	-2.06945900
C	6.28618600	-1.93435000	0.92007000
H	5.23792900	-2.27430400	2.76931000
H	7.04716300	-1.58988200	-1.06686800
H	7.26946000	-2.07334200	1.35849700

%chk=int_VII.chk

opt freq uwb97xd/6-31+g(d,p) 10f 6d gfinput gfprint

IntVII

0 3

C	2.78263100	0.86286700	0.69268800
C	2.16158200	2.19595700	0.36711900
C	1.44511500	2.70358900	1.63943200
C	3.29904800	3.16122000	-0.02197700
O	3.71066000	0.93226500	1.65256200
C	4.01423200	-0.43333800	2.01096300
C	3.51631900	-1.23921400	0.80425400
C	4.62454500	-1.67521800	-0.19353500
C	3.99405000	-2.43515300	-1.36653200
C	5.58580300	-2.62529500	0.53984300
C	5.39816400	-0.46430800	-0.73460800
N	2.54551800	-0.29339800	0.19695700
C	-2.42827800	1.83064400	-0.90321200
C	-2.56880700	2.92323400	0.20754900
C	-4.03935700	3.36530700	0.29562400
C	-1.73644300	4.15643400	-0.17418900
C	-2.14480300	2.37320400	1.57762000
N	-2.95757300	0.56096100	-0.42760200
C	1.17078100	2.14892200	-0.80831300
O	0.03035600	1.45108500	-0.55657200
C	-1.04013200	1.65039300	-1.52357800
H	0.88221300	3.61422200	1.41877700
H	2.19409300	2.93542800	2.39901600
H	0.76879400	1.94578300	2.03739800
H	2.88477300	4.15143200	-0.22049800
H	3.81465600	2.82446700	-0.92363700
H	4.01555200	3.23077500	0.79776000
H	5.08580700	-0.49163800	2.19686400
H	3.45583300	-0.66428300	2.92154700
H	2.96992400	-2.12692200	1.13054100
H	3.26930300	-1.82100800	-1.90612800
H	3.48514500	-3.33787100	-1.01258400
H	4.77335100	-2.74337600	-2.07194300

H	6.31567200	-3.03182500	-0.16753900
H	5.04448600	-3.46965700	0.98169600
H	6.15056200	-2.12819400	1.33560100
H	5.86927800	0.12014900	0.06358800
H	4.74023900	0.19744200	-1.30637200
H	6.19240200	-0.80163500	-1.40817600
H	-4.70096400	2.53733900	0.56868900
H	-4.39132500	3.79463700	-0.64998500
H	-4.14773400	4.12928000	1.07235600
H	-0.66327800	3.95665100	-0.17389600
H	-1.92034800	4.96130200	0.54451600
H	-2.00717200	4.53096900	-1.16834800
H	-2.06126700	3.19348400	2.29919600
H	-1.19505900	1.84346900	1.53107500
H	-2.88261600	1.66208900	1.95930700
H	-3.03067800	2.18031500	-1.76148600
H	-0.78778000	2.53023100	-2.11311500
H	-1.02255300	0.78866300	-2.19431000
Ni	0.64204000	-0.92828600	-0.24708300
Cl	0.83217300	-1.09637200	-2.60202300
Cl	0.08300200	-0.82607500	2.01559000
O	1.05215000	-3.01064800	-0.12317400
H	0.26045000	-3.32421500	0.33291600
O	-1.30566900	-1.56817500	-0.61438700
H	0.96851500	-3.26038900	-1.05482700
H	-1.92226300	-0.83892800	-0.34586600
O	1.34076000	2.77532100	-1.82207100
H	-1.32781600	-1.60608800	-1.58170200
C	-4.17874800	0.27468000	-0.64941600
H	-4.81386900	0.93262500	-1.26060300
C	-4.82024800	-0.93151400	-0.10429800
C	-4.24151000	-1.64387600	0.95507300
C	-6.03995800	-1.35759000	-0.64024800
C	-4.86993200	-2.78044700	1.44813100
H	-3.30940600	-1.29950600	1.39368200
C	-6.66430700	-2.49889500	-0.14650100
H	-6.49669900	-0.79642000	-1.45171300
C	-6.07738500	-3.21167900	0.89704700
H	-4.41968800	-3.32785800	2.26971500
H	-7.60654700	-2.82916000	-0.57154500
H	-6.56493300	-4.09942700	1.28774500

%chk=product2b.chk
opt freq uwb97xd/6-31+g(d,p) 10f 6d gfinput gfprint

product2b C0

0 1

C	0.28912900	0.79836400	2.58692400
C	1.25540400	0.20173500	3.40097200
C	2.37685600	0.92323600	3.80527800
C	2.53268000	2.24634800	3.40127900
C	1.57377400	2.84352900	2.58027200
C	0.46004500	2.12373300	2.17021900
C	-0.90096200	0.02320700	2.19250100
N	-1.79123800	0.49171600	1.41749100
C	-2.91651800	-0.35985700	1.07417800
C	-2.58357000	-1.04110300	-0.24961900
O	-1.56084900	-1.99980500	0.05972400
C	-4.23092200	0.46506400	1.03868900
C	-4.19449700	1.49734200	-0.09441900
C	-4.37219600	1.18878400	2.38592200
C	-5.43446000	-0.47250500	0.85811300
C	-1.00302800	-2.67668200	-0.94498200
C	0.06816700	-3.62805200	-0.40009800
C	-0.57303300	-4.63122500	0.56714800
C	0.76247700	-4.35103600	-1.56221500
O	-1.32119500	-2.55645200	-2.10559200
C	1.09700300	-2.76897000	0.34521300
O	1.54652700	-1.80442200	-0.45815500
C	2.56841300	-0.92213300	0.03250700
C	2.87599600	0.02193800	-1.12591200
C	4.20499500	0.80655600	-0.96045900
C	4.21559300	1.59260300	0.35581100
C	4.32726600	1.78283400	-2.13976700
C	5.39585700	-0.16329800	-1.00235700
N	1.75616200	0.93924100	-1.25494600
O	1.47877700	-2.94569900	1.47891200
C	0.83064500	0.64067500	-2.07203700
C	-2.56778500	3.12819600	-2.75541700
C	-1.57593500	3.54010400	-1.86279700
C	-0.47482300	2.72981700	-1.62033600
C	-0.35124800	1.49877900	-2.27479900
C	-1.35162200	1.08586000	-3.15896700
C	-2.45829700	1.89807600	-3.39787000
H	1.13636600	-0.83570800	3.70221000
H	3.12600200	0.45194500	4.43367900
H	3.40596300	2.81071100	3.71388200
H	1.70352700	3.87156600	2.25596600
H	-0.29339200	2.56540800	1.52614900
H	-0.97533000	-0.98914900	2.61731100
H	-2.20002200	-0.32554600	-0.98076300
H	-3.43940200	-1.56972700	-0.67548300
H	-4.19291400	1.02145400	-1.08100200
H	-3.29782300	2.11839000	-0.02266700
H	-5.07460400	2.14811400	-0.04420300
H	-3.55206800	1.89543200	2.53943100
H	-4.36719000	0.47599200	3.21942100
H	-5.31646300	1.74261000	2.42122500
H	-6.36552100	0.09901400	0.93347000
H	-5.45601800	-1.24759000	1.63305300
H	-5.43821600	-0.96660700	-0.11858400
H	-3.04818000	-1.16068200	1.82351300
H	-1.03442200	-4.12511400	1.41626800
H	0.19008300	-5.31301300	0.94947300
H	-1.33441100	-5.21663200	0.04315100

H	0.03882900	-4.97304800	-2.09432100
H	1.55848100	-4.99426200	-1.17552900
H	1.18859900	-3.64136100	-2.27280300
H	5.11794700	2.21084100	0.42077700
H	4.20476700	0.93005700	1.22818800
H	3.34042700	2.24385800	0.42191900
H	3.51150800	2.51093200	-2.13201300
H	4.29785900	1.24977600	-3.09774800
H	5.27641200	2.32666500	-2.08517400
H	5.37966700	-0.77587600	-1.91135400
H	5.42006900	-0.83516100	-0.13865900
H	6.33462100	0.40047700	-0.99819700
H	2.97552500	-0.60569600	-2.02914900
H	2.19221300	-0.38014500	0.90342200
H	3.43340900	-1.52105200	0.32564100
H	0.86781500	-0.27342900	-2.68311800
H	-3.42923600	3.76273500	-2.93978500
H	-1.66732900	4.49559500	-1.35534600
H	0.30822600	3.03129800	-0.93202100
H	-1.26628300	0.11998800	-3.65067500
H	-3.23249100	1.56976800	-4.08432000

Crystallographic data for compound 2d

CCDC 2385941 contains the supplementary crystallographic data for this paper. These data can be obtained free of charge from The Cambridge Crystallographic Data Centre via www.ccdc.cam.ac.uk/structures

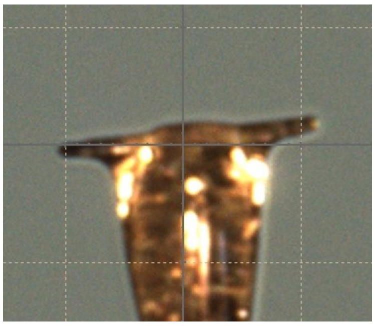

Figure S3. X-ray quality specimen of **2d**. The crystal has needle habit, transparent, colourless with dimensions 0.105 x 0.015 x 0.015 mm.

A X-ray quality sample was retrieved from the batch obtained directly from the mother liquor by slow evaporation at room temperature. The crystal was cut from a larger agglomerate using a blade and polished by mechanical ablation in a drop of perfluorinated oil.

The data collection was carried out at room temperature on a Rigaku XtaLAB Synergy-S k-geometry diffractometer equipped with a HyPix area detector by using monochromated Cu Kα (λ = 1.54184 Å) radiation from PhotonJet micro-focus sealed X-ray tube. A 80.7 % complete dataset was recorded, covering a hemisphere up to $\sin\vartheta/\lambda$ = 0.55 $Å^{-1}$, with 11 independent runs at variable crystal-to-detector distance. Overall, 10758 diffraction effects were measured (3705 independent). The CrysAlisPRO [CrysAlisPRO, Oxford Diffraction /Agilent Technologies UK Ltd, Yarnton, England] suite of programs was employed throughout to carry out data reduction and scaling procedures. The sample was heavily twinned. After de-convolution, due to poor statistics, only the major component was considered for the development of the structural model. Structure solution and refinement were carried out by means of Shelxs and Shexl programs. [2]

The compound **2d** crystallizes in the orthorhombic Sohncke space group $P2^12^12^1$. The estimated unit cell parameters (Å, deg) were a = 5.9800(3), b = 12.1226(3), c = 45.977(3), α = 90., β = 90., γ = 90., V = 3333.0(3), from 3542 intense reflections among 7.5 e 125.6 deg of 2ϑ (final integration result). The final least-squares model converged to R1(F) = 0.0581 for 2487 Fo > 4σ(Fo), 0.0965 for all the 3705 independent data with highest Fourier residuals as large as +0.57 / –0.40 e/$Å^3$, both close to Br atoms.

The compound is chiral with asymmetric carbon atoms at C8 and C20 (Figure S4) and crystallizes as a *S*,*S* pure enantiomer. Figure S4 shows the absolute configuration of the chiral centres (C8(S) and C20(S)). The absolute structure is secured by anomalous dispersion effect prompted by the Cu Kα radiation, as testified by the refined Flack parameter (0.00(3)). [3]

[2] Sheldrick, G. M. A short history of Shelx, Acta Crystallogr. Section A, **2008**, *64*, 112-122.
[3] Parsons, S.; Flack, H. D.; Wagner, T. Acta Crystallogr. Section B, **2013**, *69*, 249-259.

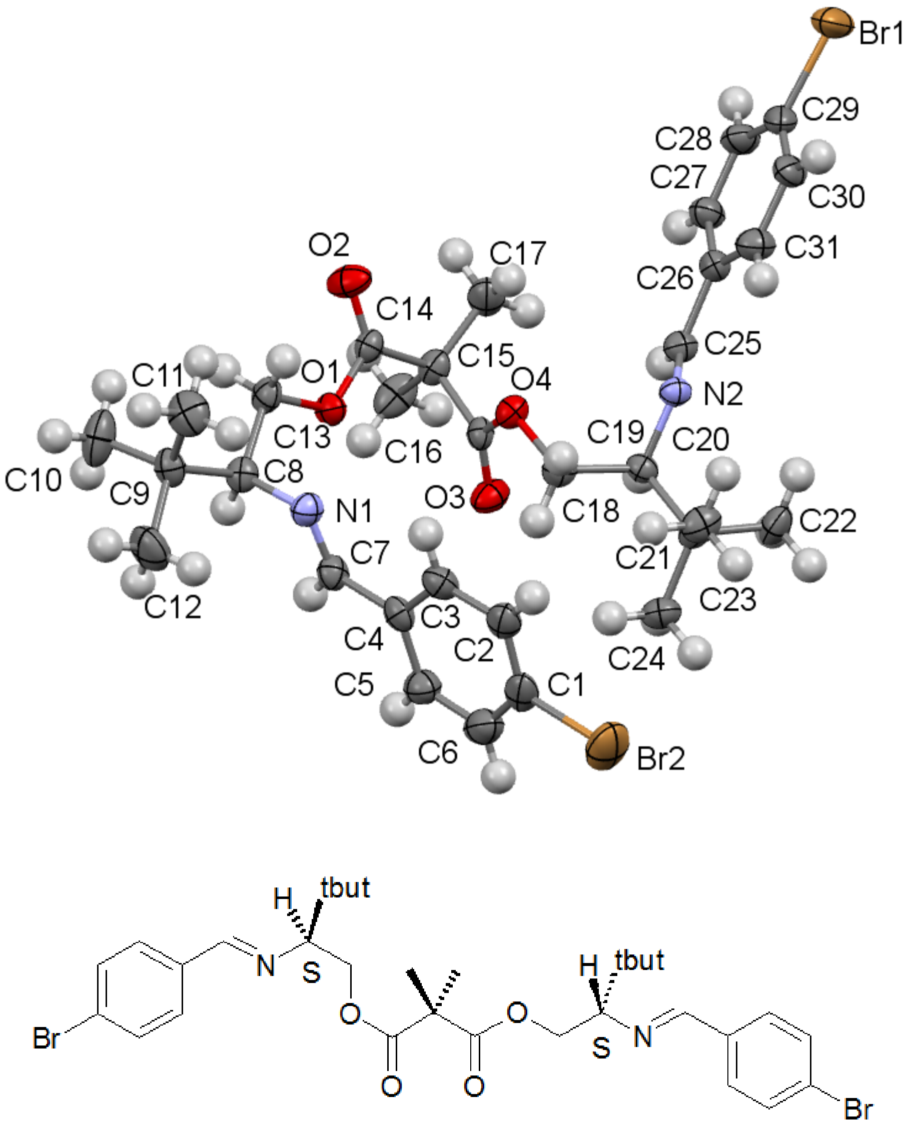

Figure S4. Up: Asymmetric unit of **2d** at RT, with the atom-numbering scheme. Thermal ellipsoids of non-H atoms were drawn at the 25 % probability level. The usual colour code was employed for atoms (grey: C; white: H; blue: N; red: O; dark yellow: Br). Down: Molecular structure of **2d**, with the Cahn-Ingold-Prelog descriptors highlighted

As for the crystal packing (Figure S5), no strong hydrogen bond donors are present, so the only relevant contact with distance H···acceptor lower than the sum of the van der Waals radii is a weak intermolecular one between the C16 methyl group, and the ester O4 oxygen as an acceptor (Table S4).

Table S4. CH···O contacts in **2d**.

D–H···A	d_{D-H}, Å	$d_{H\cdots A}$, Å	$d_{D\cdots A}$, Å	α_{DHA}, deg	Symmetry operation
C16–H16···O4	0.96	2.52	3.374(13)	148	1+x,y,1/z

Overall, the packing is likely dominated by steric requirements, with translation-dependent molecules. The asymmetric unit is folded in a "zeta" fashion so that no internal molecular symmetry elements survive. Adjacent molecules along *a* and *c* are wrapped along the corresponding 2_1 axes to maximize the distance between bulky *t*-butyl groups, which project into the free space between closely packed molecules. No stacking interactions are formed at all.

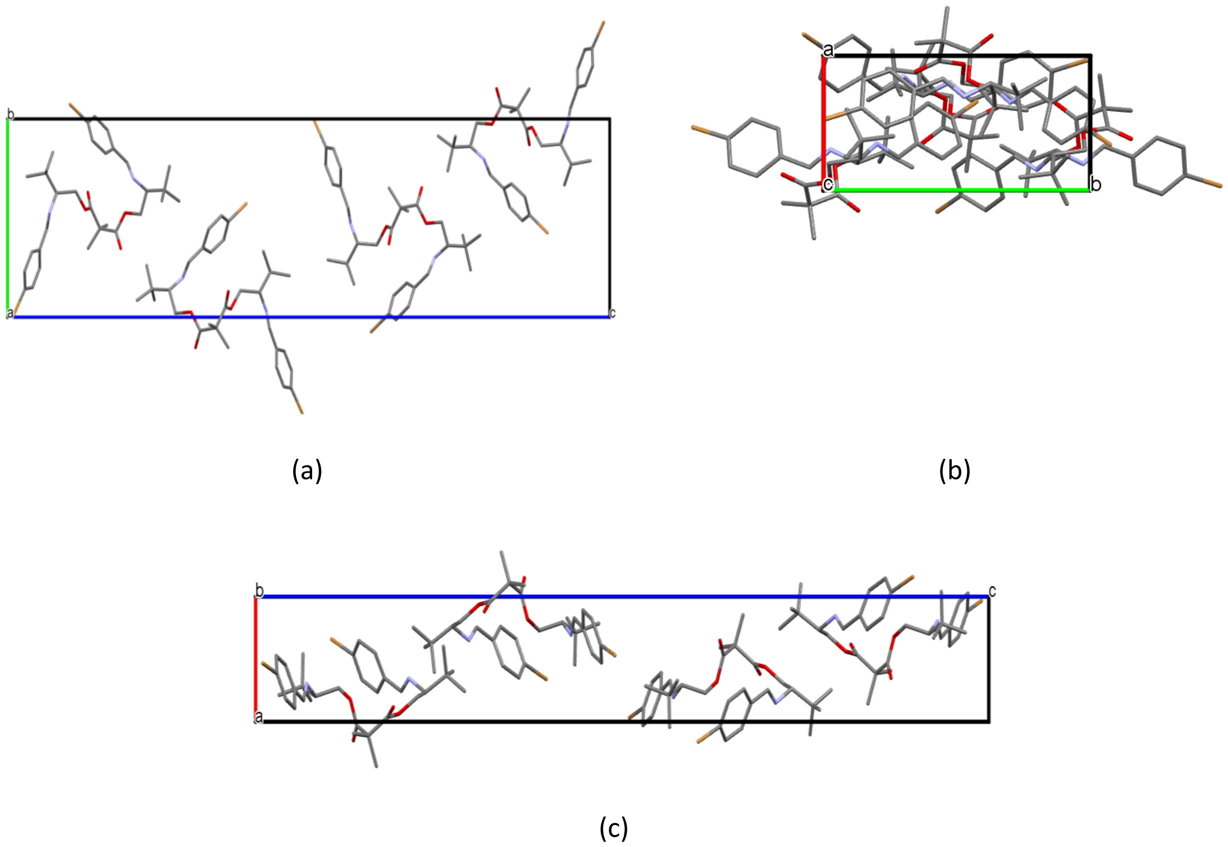

Figure S5. Crystal packing of **2d** at RT, as seen (a) along the *a* cell axis; (b) the *c* cell axis; (c) the *b* cell axis. Colour code as in Figure S4. The crystallographic reference system is also shown. Hydrogen atoms were omitted for clarity.

^{1}H NMR spectra of known compounds

(*S*)-4-Isopropyl-2-phenyl-4,5-dihydrooxazole (14)

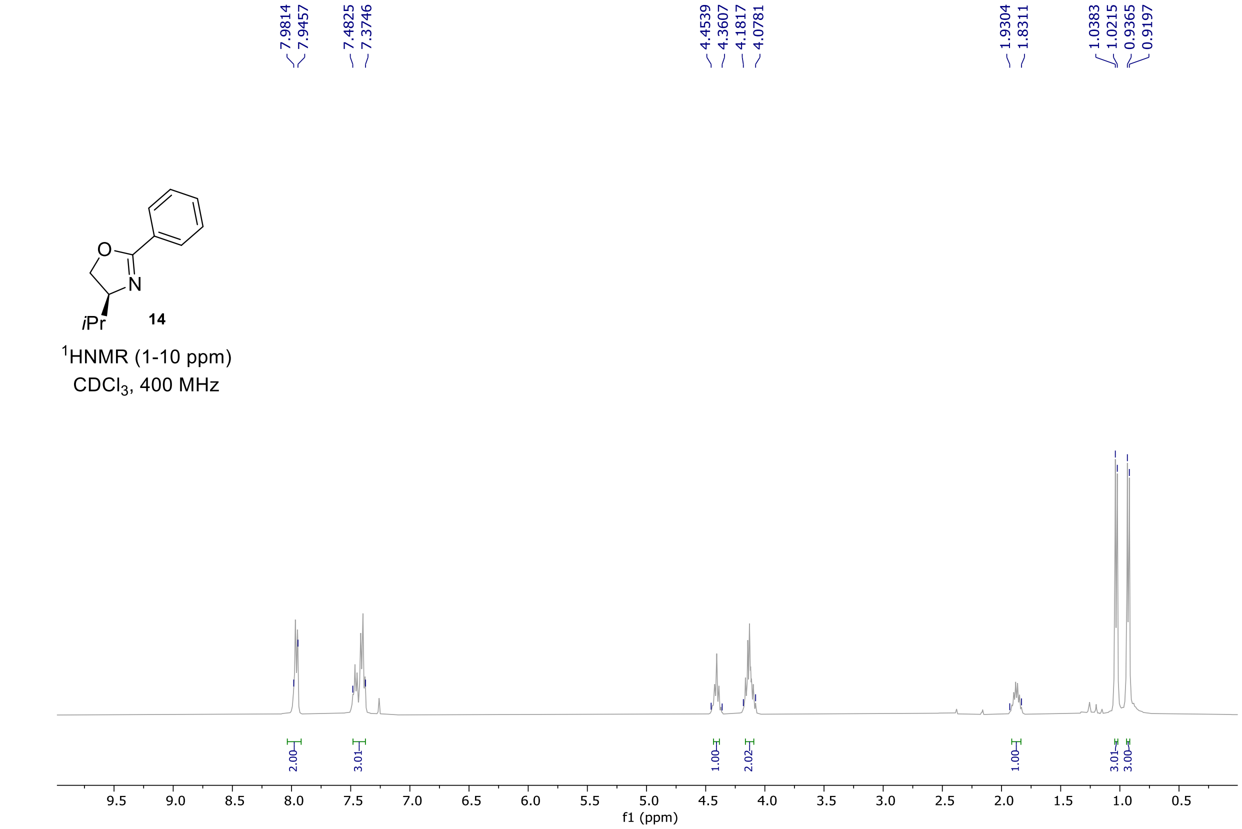

(*S*)-*N*-(1-Hydroxy-3-methylbutan-2-yl)benzamide (16)

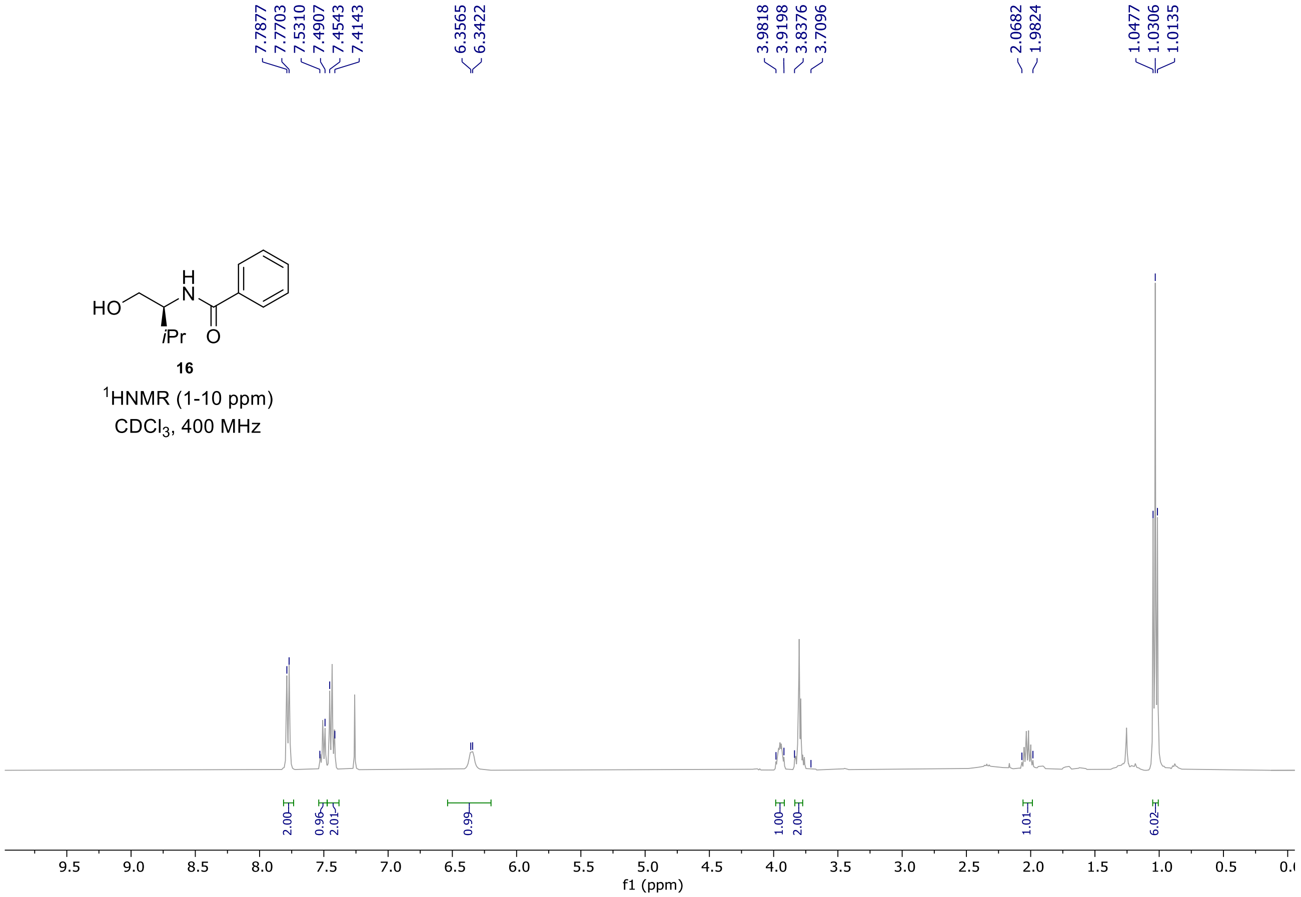

^{1}H NMR and ^{13}C NMR spectra of unknown compounds

Bis((*S*)-3,3-dimethyl-2-((thiophen-2-ylmethylene)amino)*tert*-butyl) 2,2-dimethylmalonate (2a)

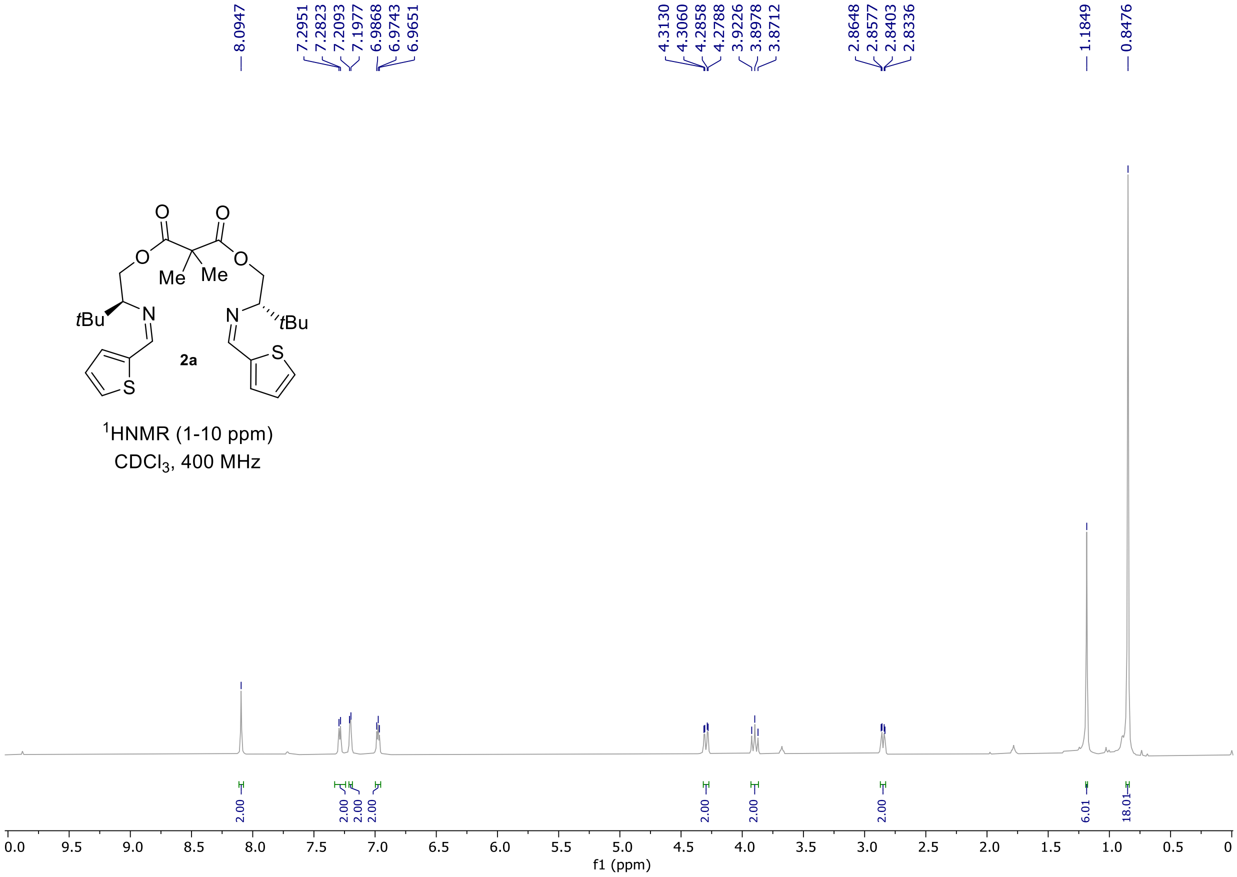

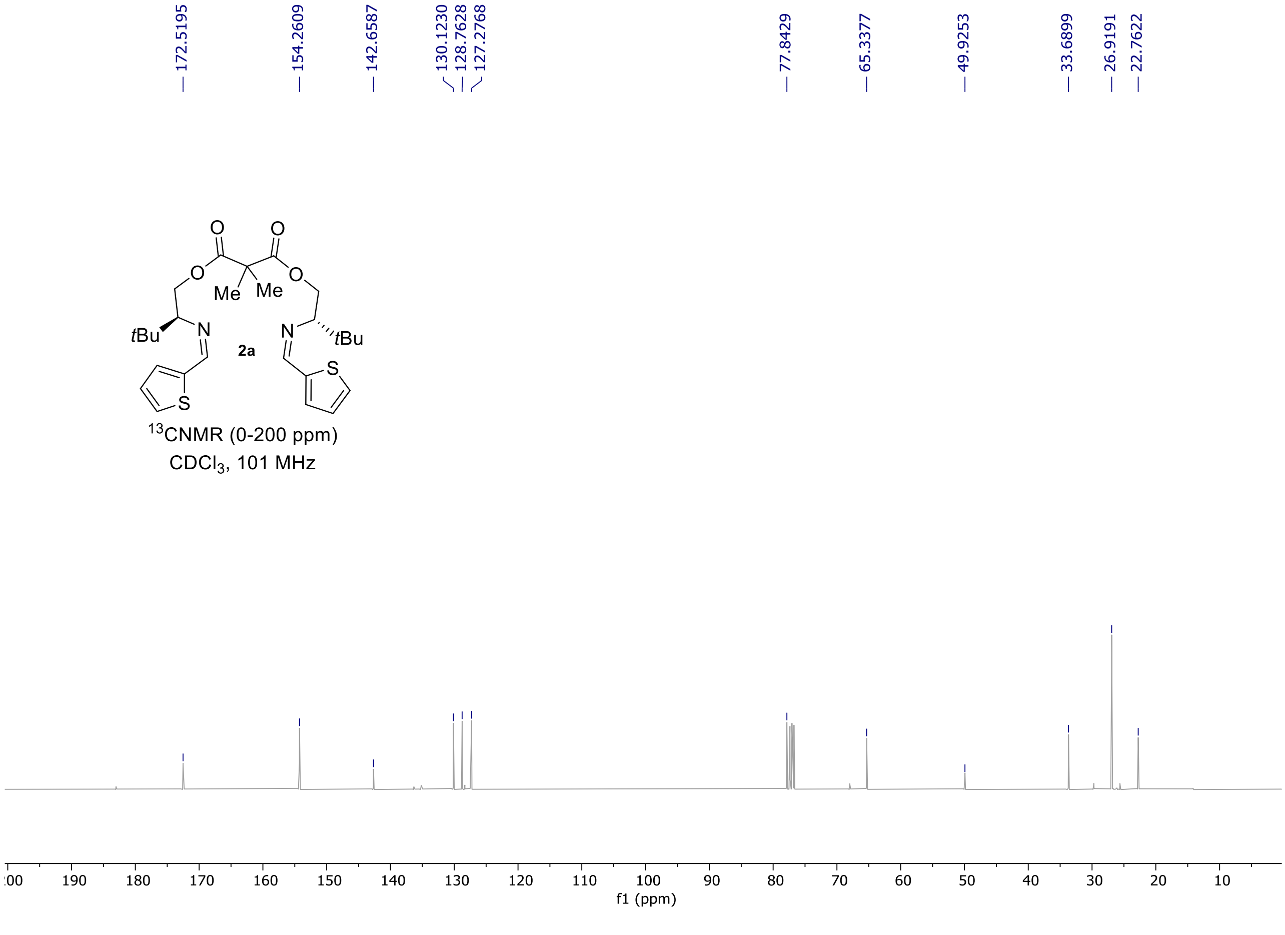

Bis((*S*)-3,3-dimethyl-2-((benzylidene)amino)*tert*-butyl) 2,2-dimethylmalonate (2b)

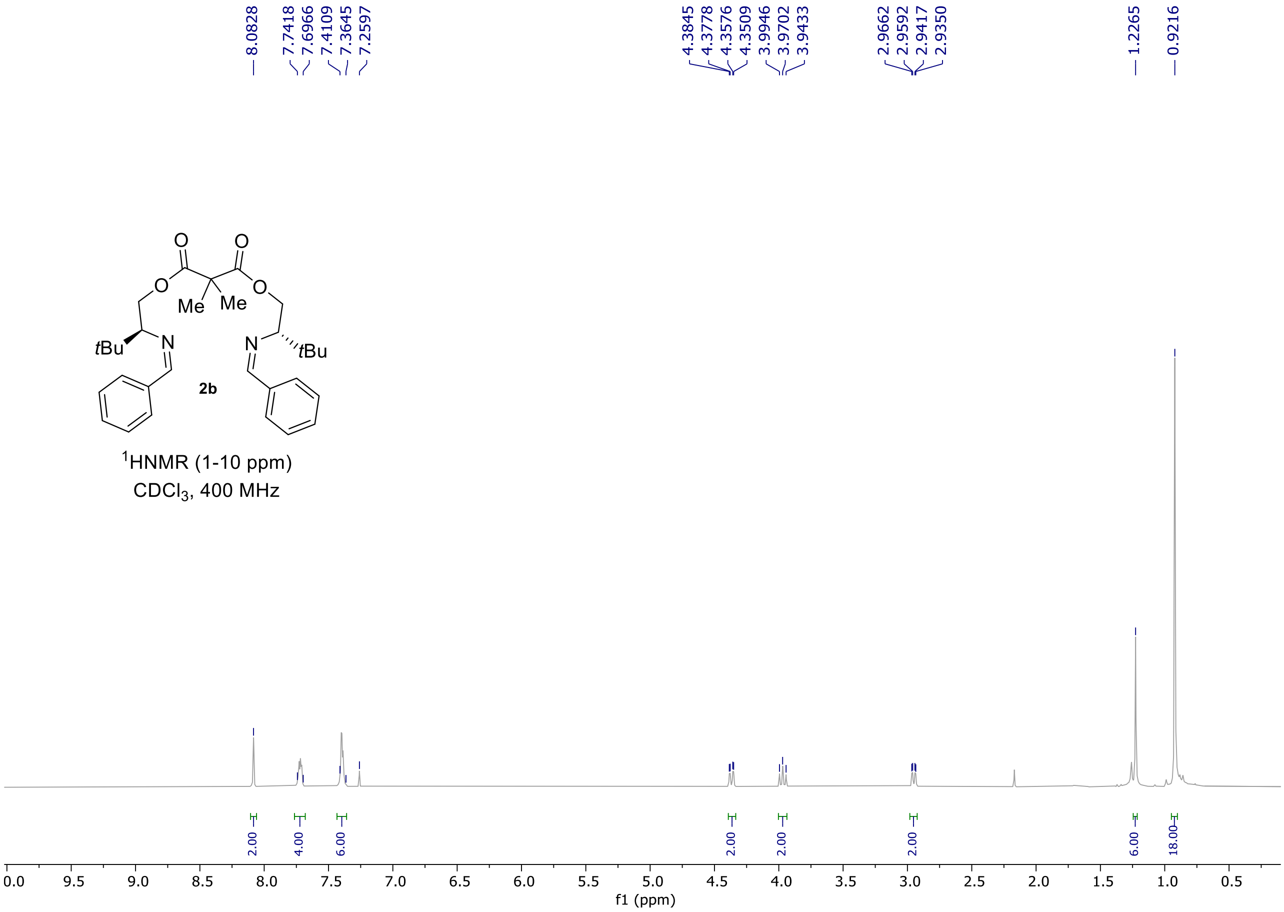

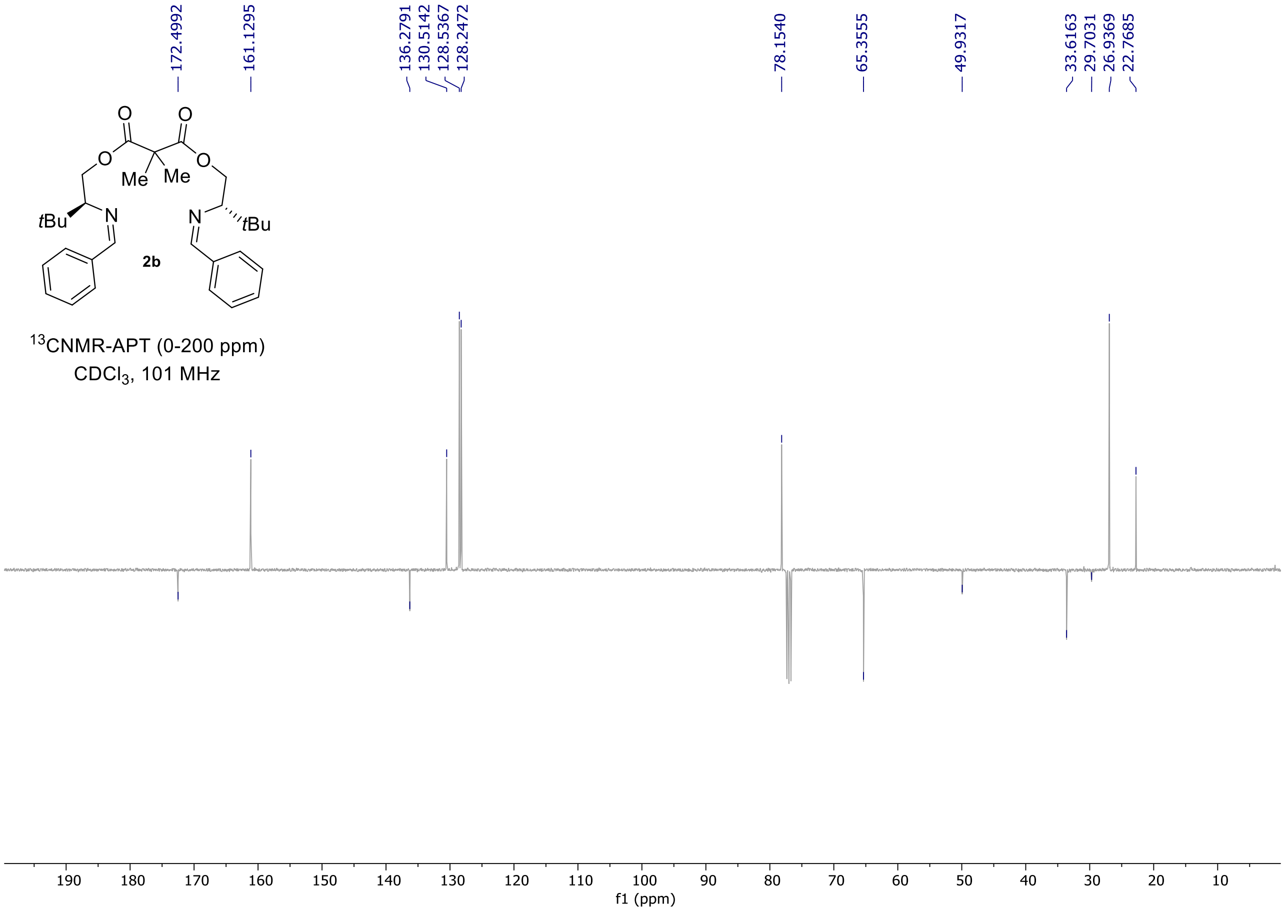

Bis((*S*)-3,3-dimethyl-2-((4-methoxybenzylidene)amino)*tert*-butyl) 2,2-dimethylmalonate (2c)

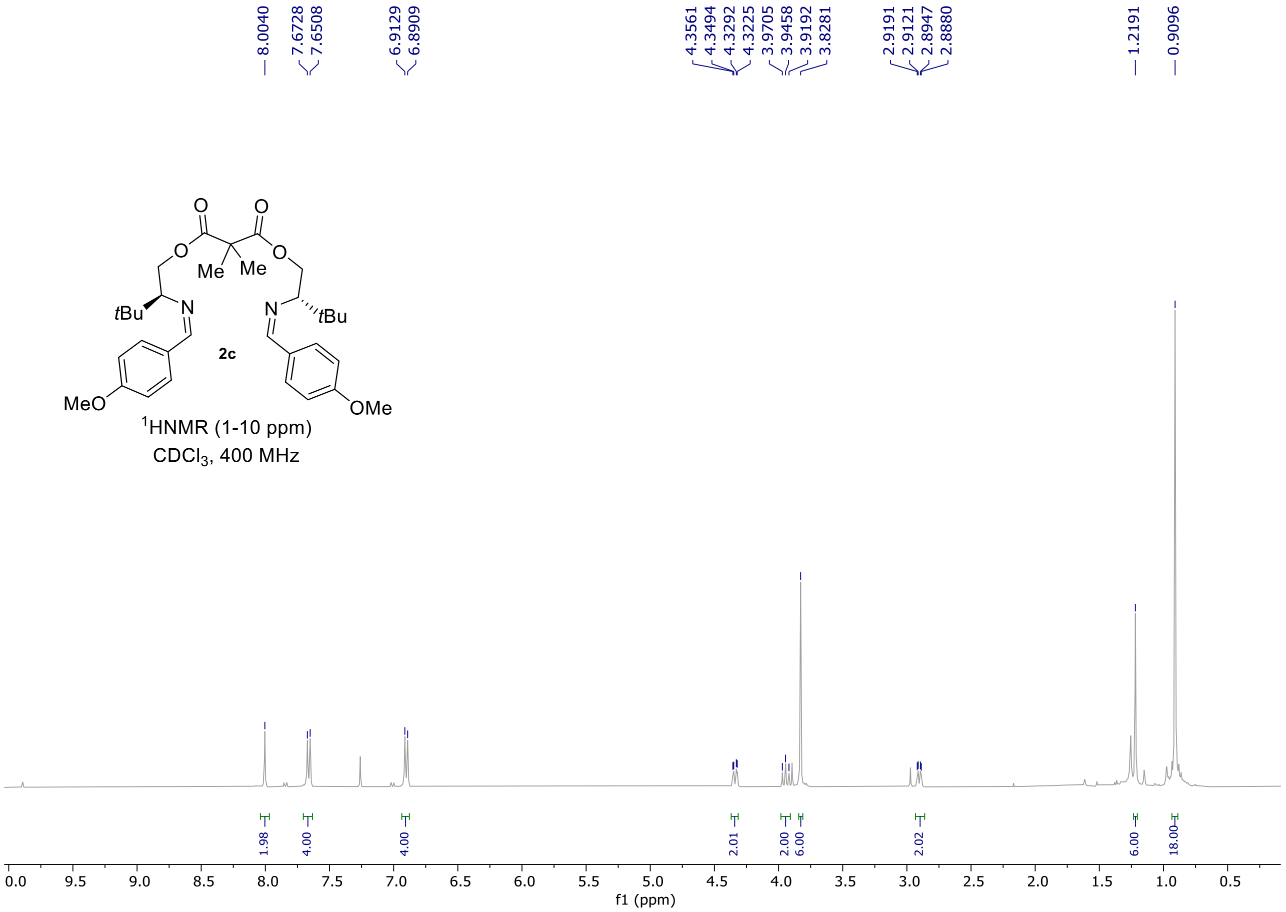

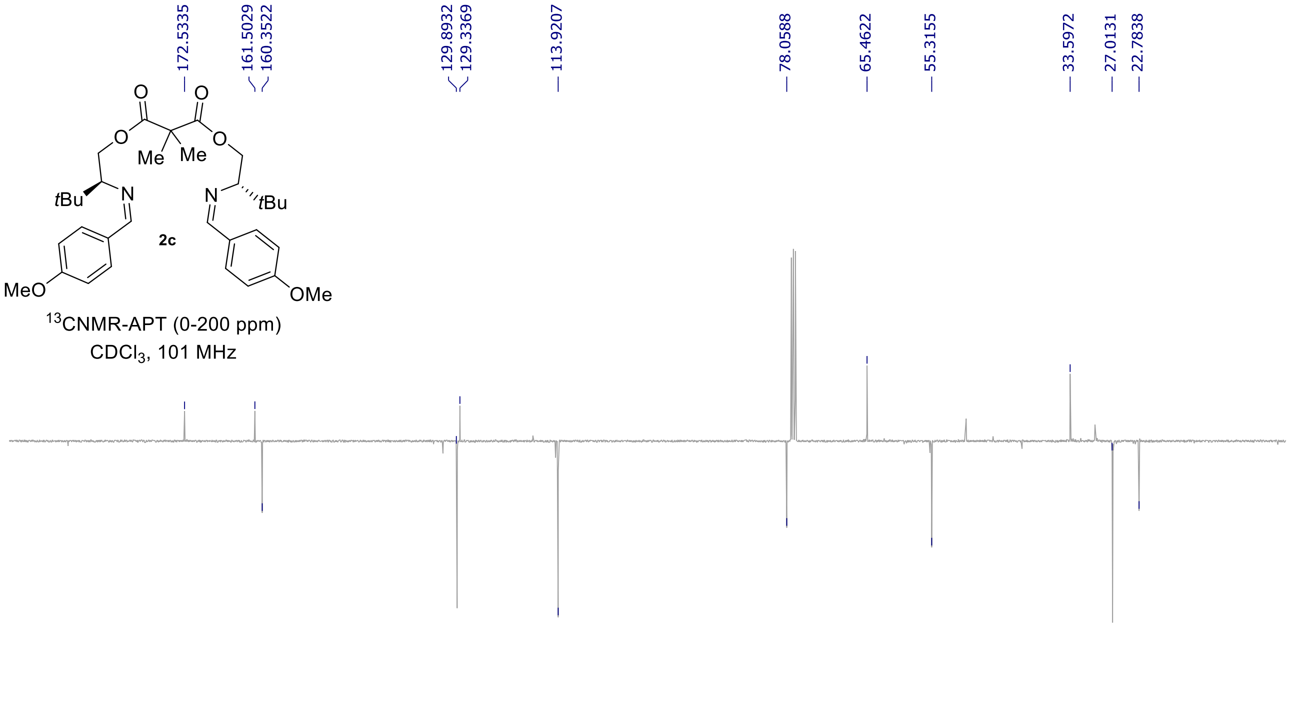

Bis((*S*)-3,3-dimethyl-2-((4-bromobenzylidene)amino)*tert*-butyl) 2,2-dimethylmalonate (2d)

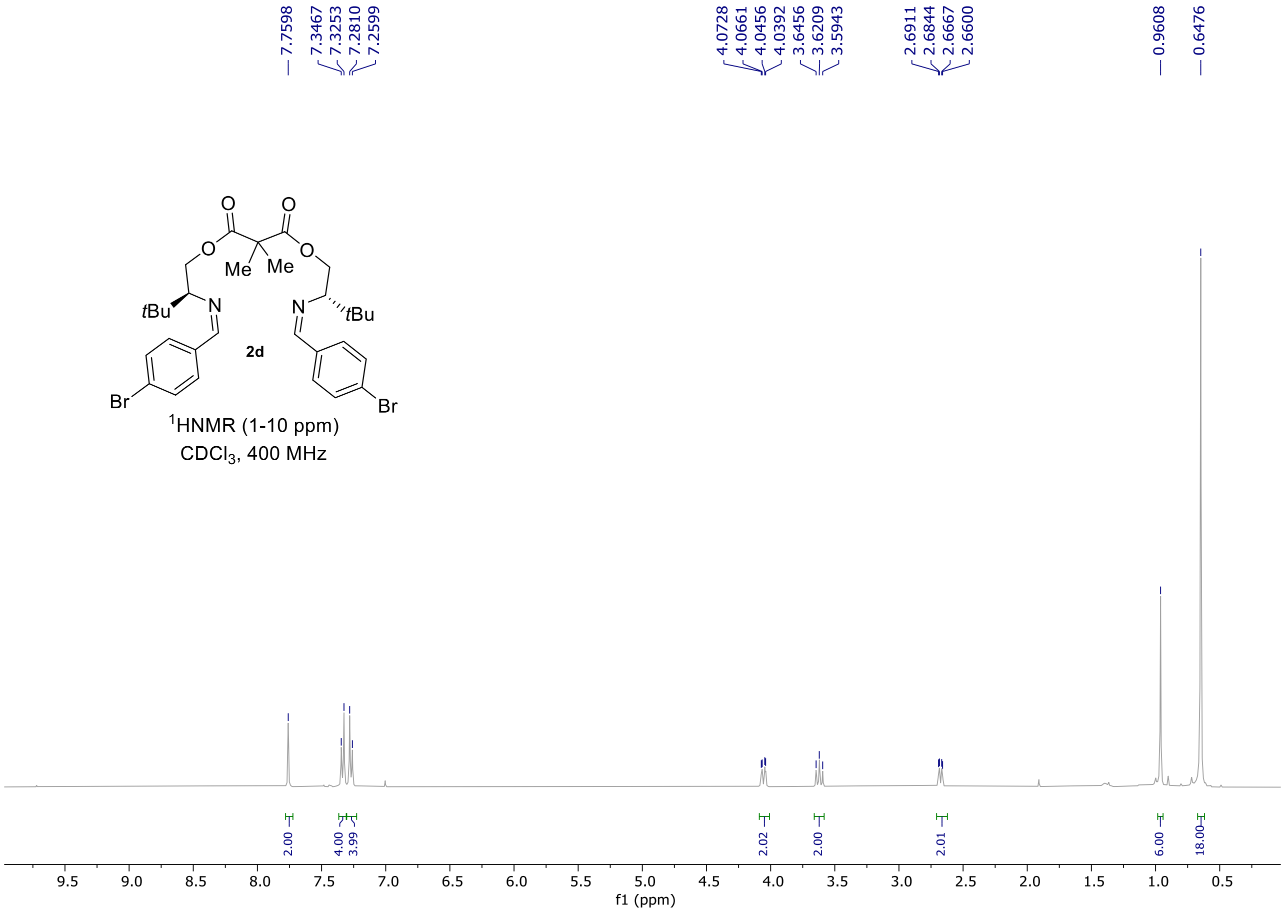

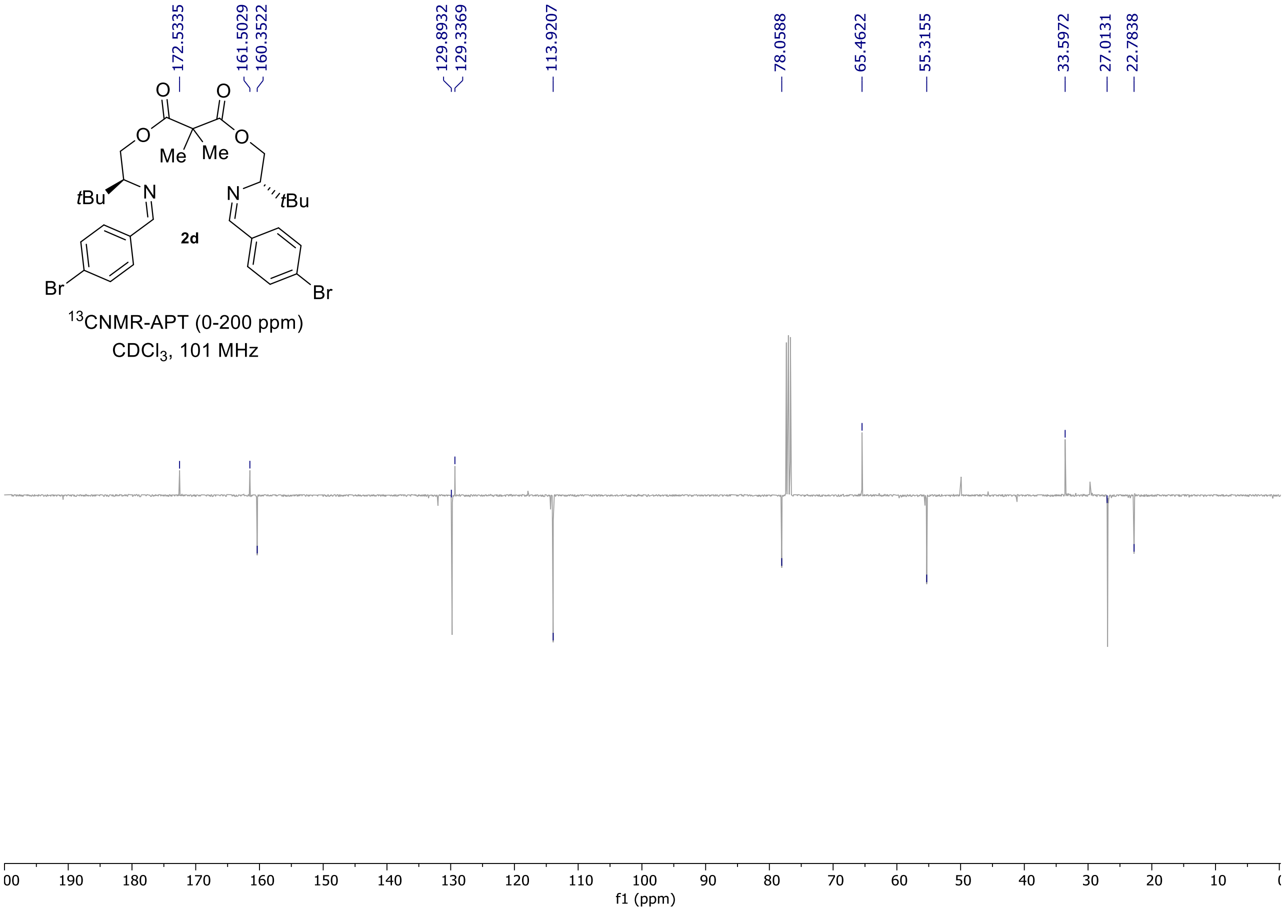

Bis((*S*)-3,3-dimethyl-2-((4-nitrobenzylidene)amino)*tert*-butyl) 2,2-dimethylmalonate (2e)

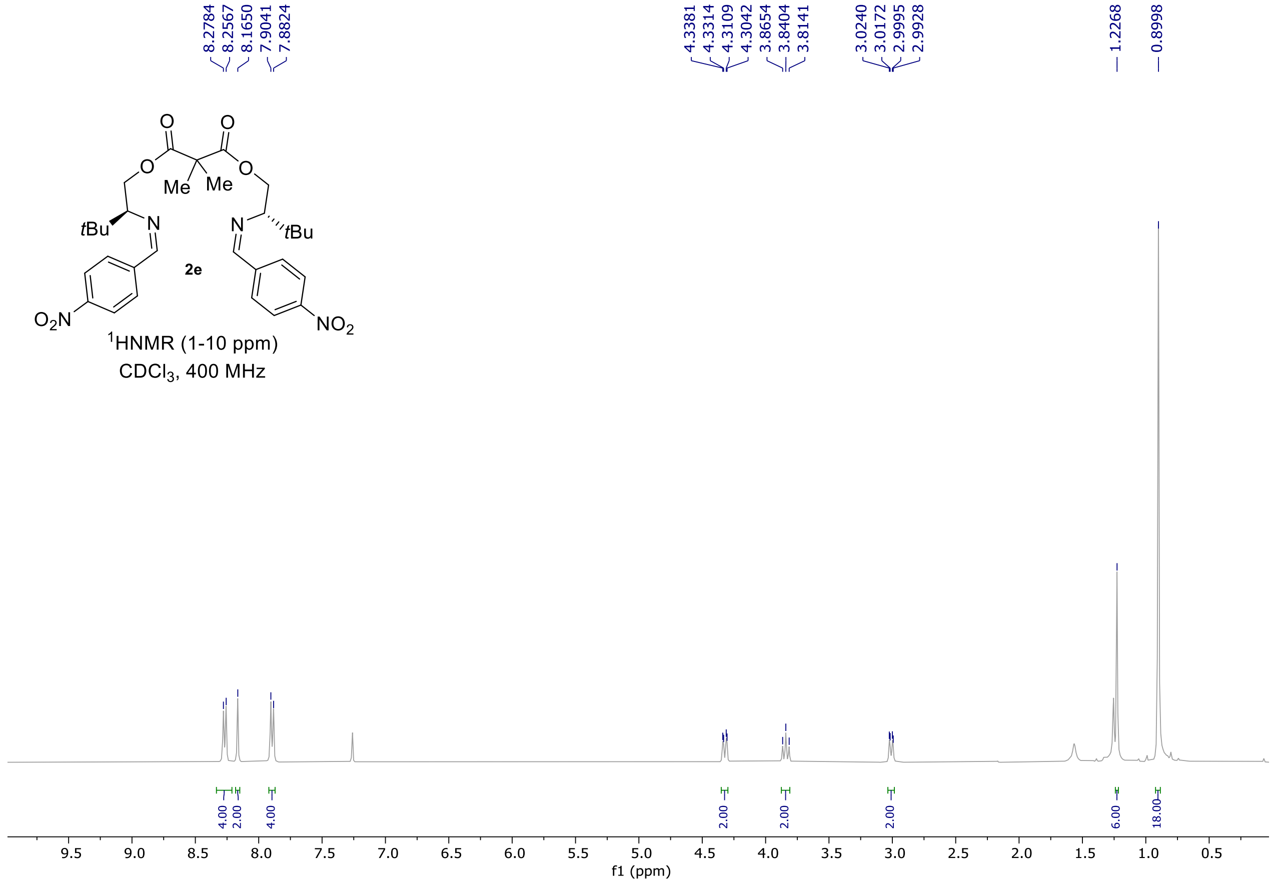

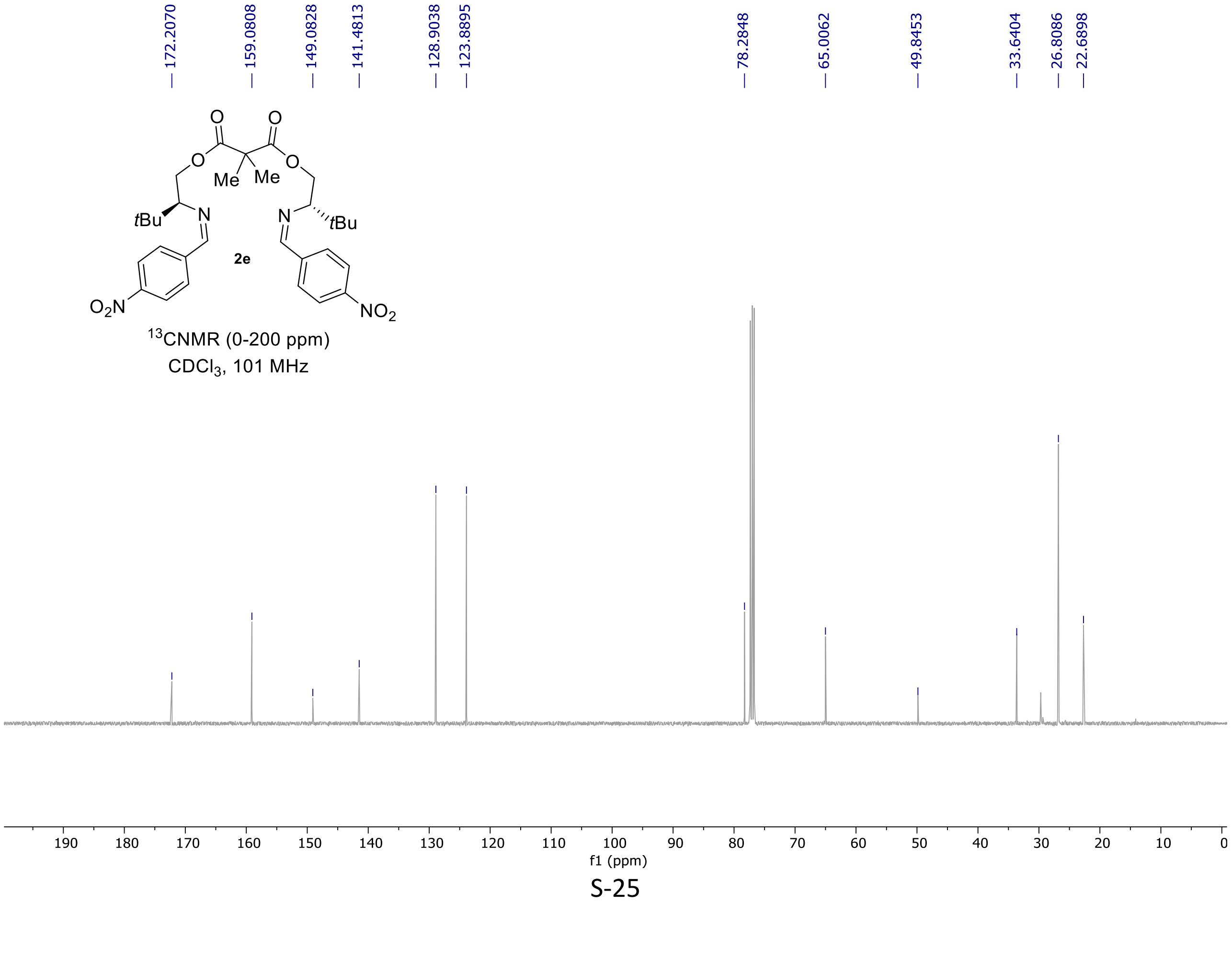

Bis((*S*)-3,3-dimethyl-2-((2-nitrobenzylidene)amino)*tert*-butyl) 2,2-dimethylmalonate (2f)

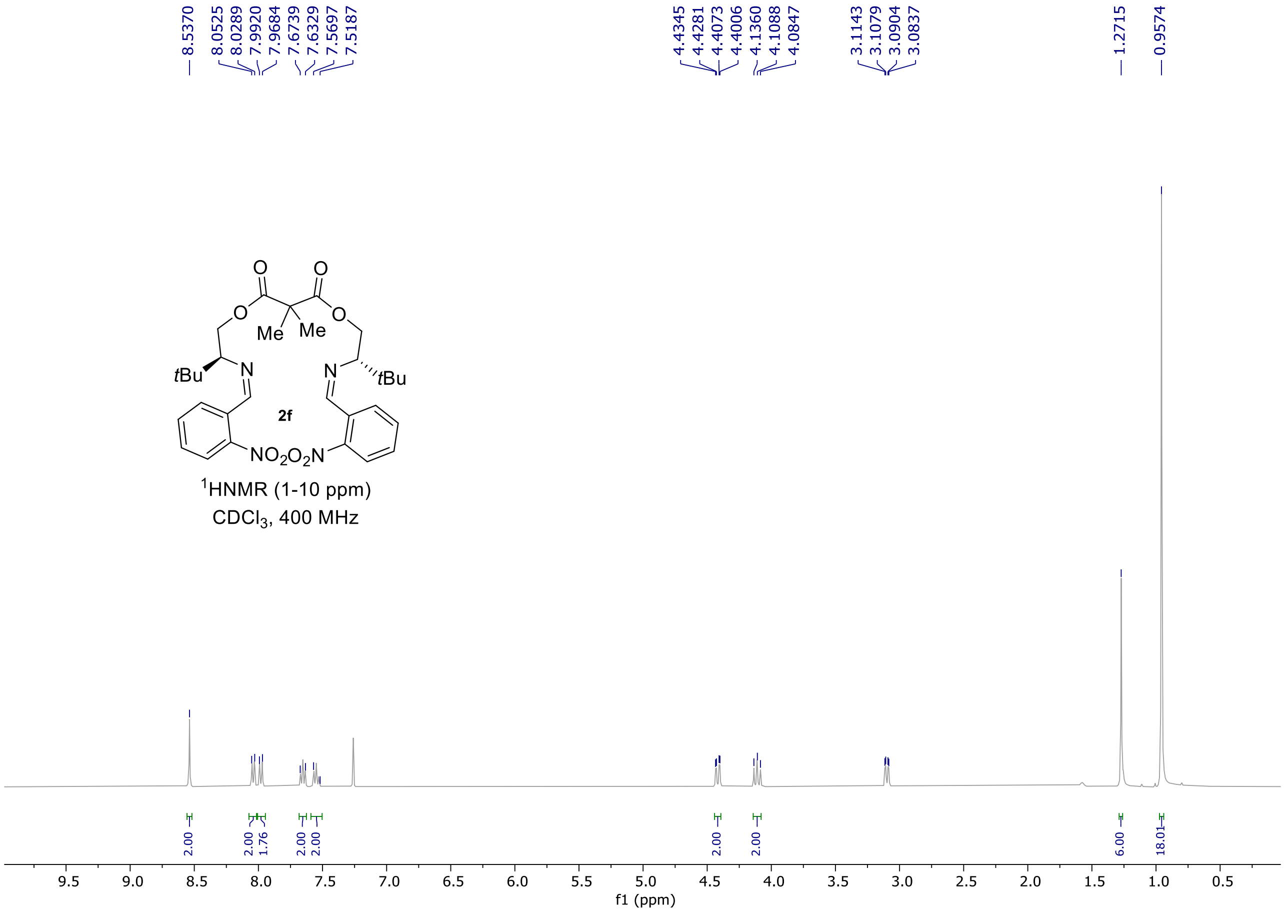

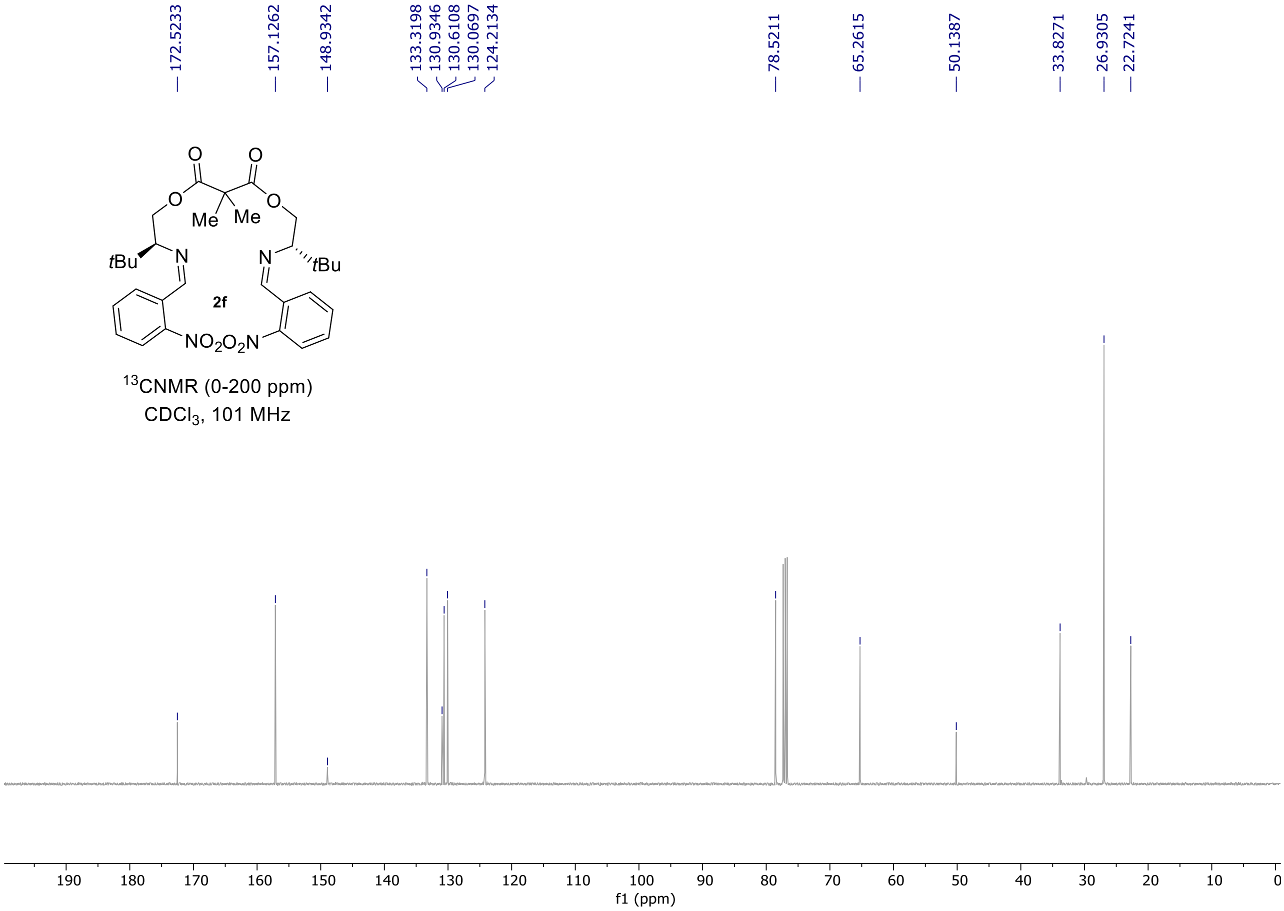

Bis((*S*)-3,3-dimethyl-2-((3-trifluoromethylbenzylidene)amino)*tert*-butyl) 2,2-dimethylmalonate (2g)

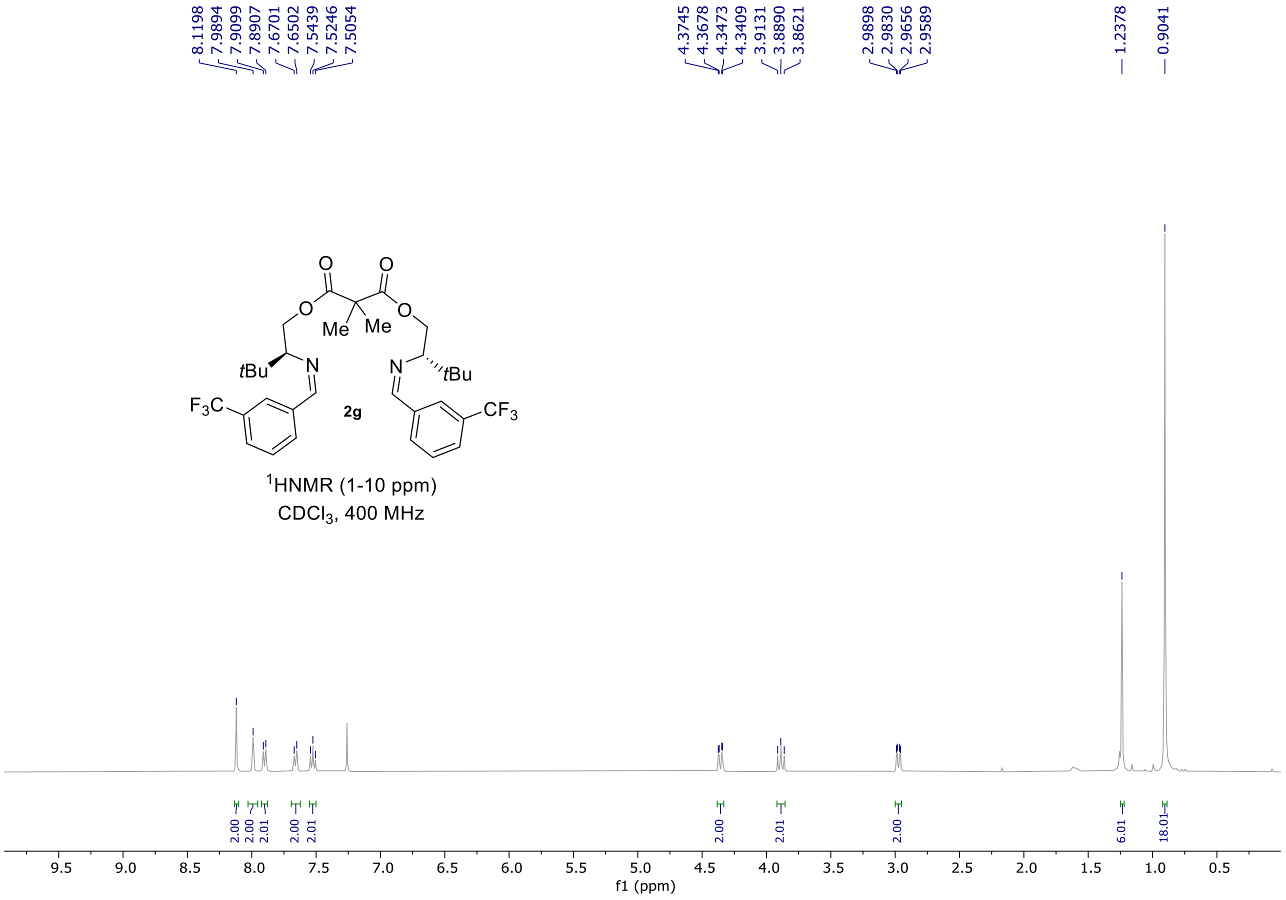

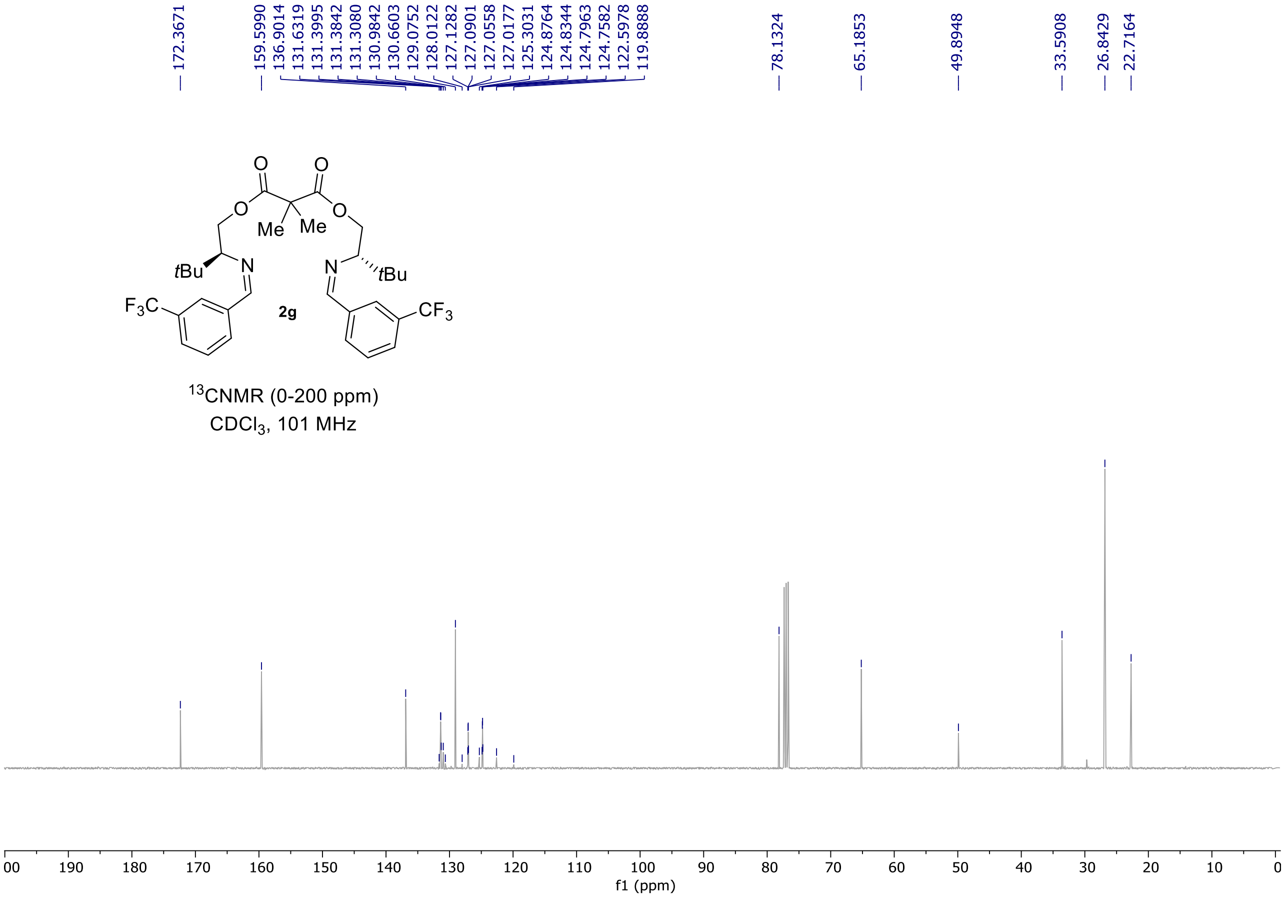

Bis((*S*)-3,3-dimethyl-2-((naphthalen-2-ylmethylene)amino)*tert*-butyl) 2,2-dimethylmalonate (2h)

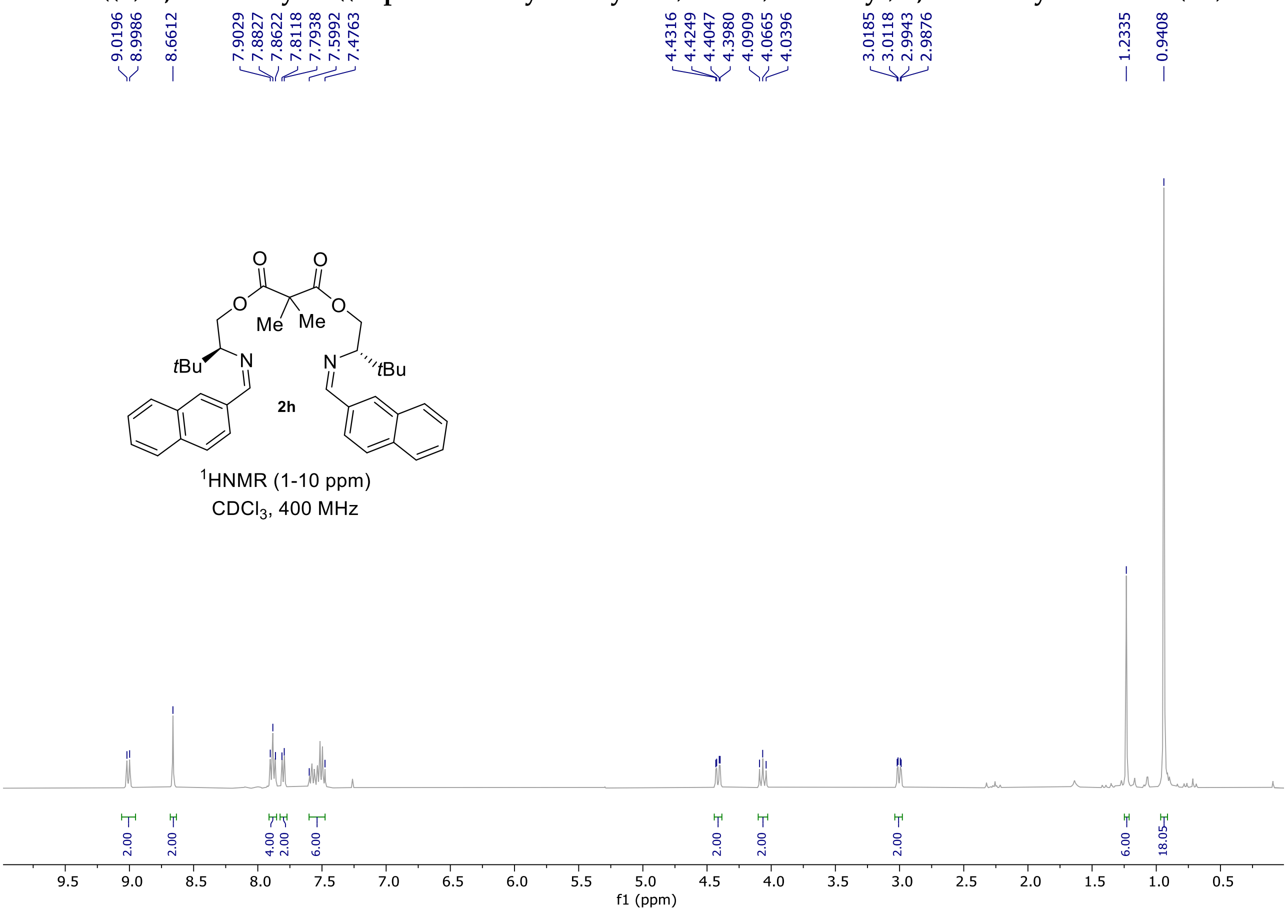

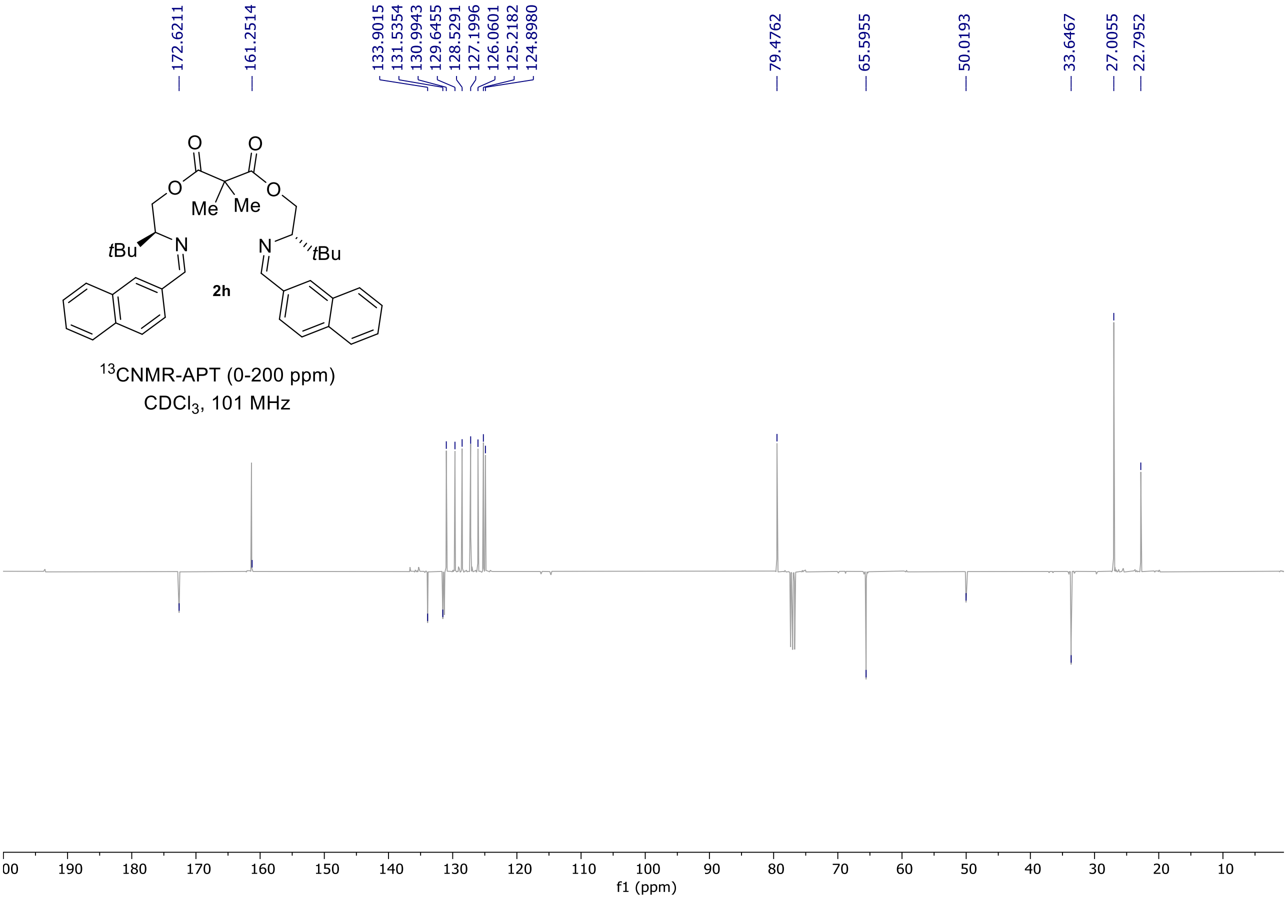

Bis((*S*)-3,3-dimethyl-2-((benzo[*d*][1,3]dioxol-5-ylmethylene)amino)*tert*-butyl) 2,2-dimethylmalonate (2i)

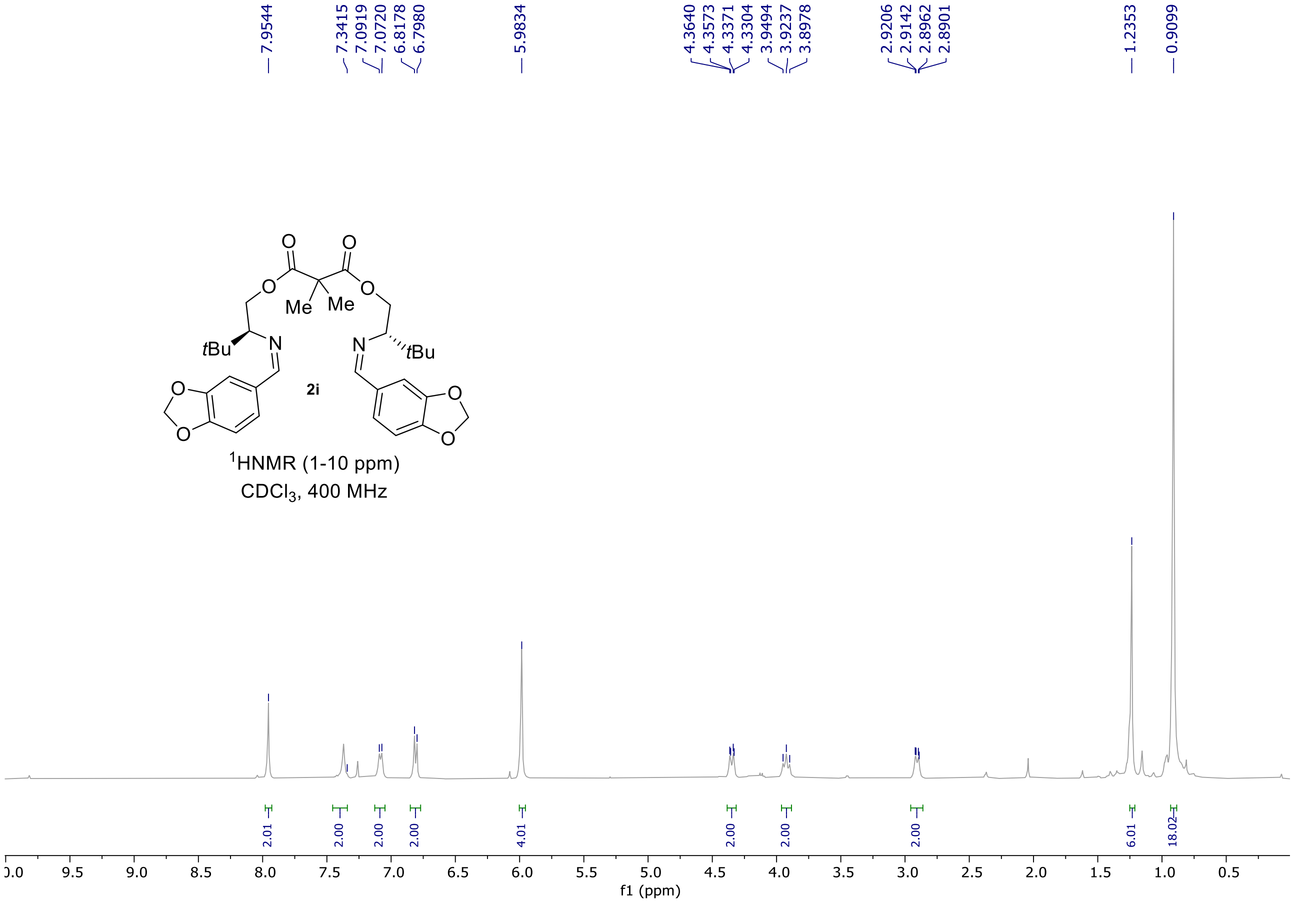

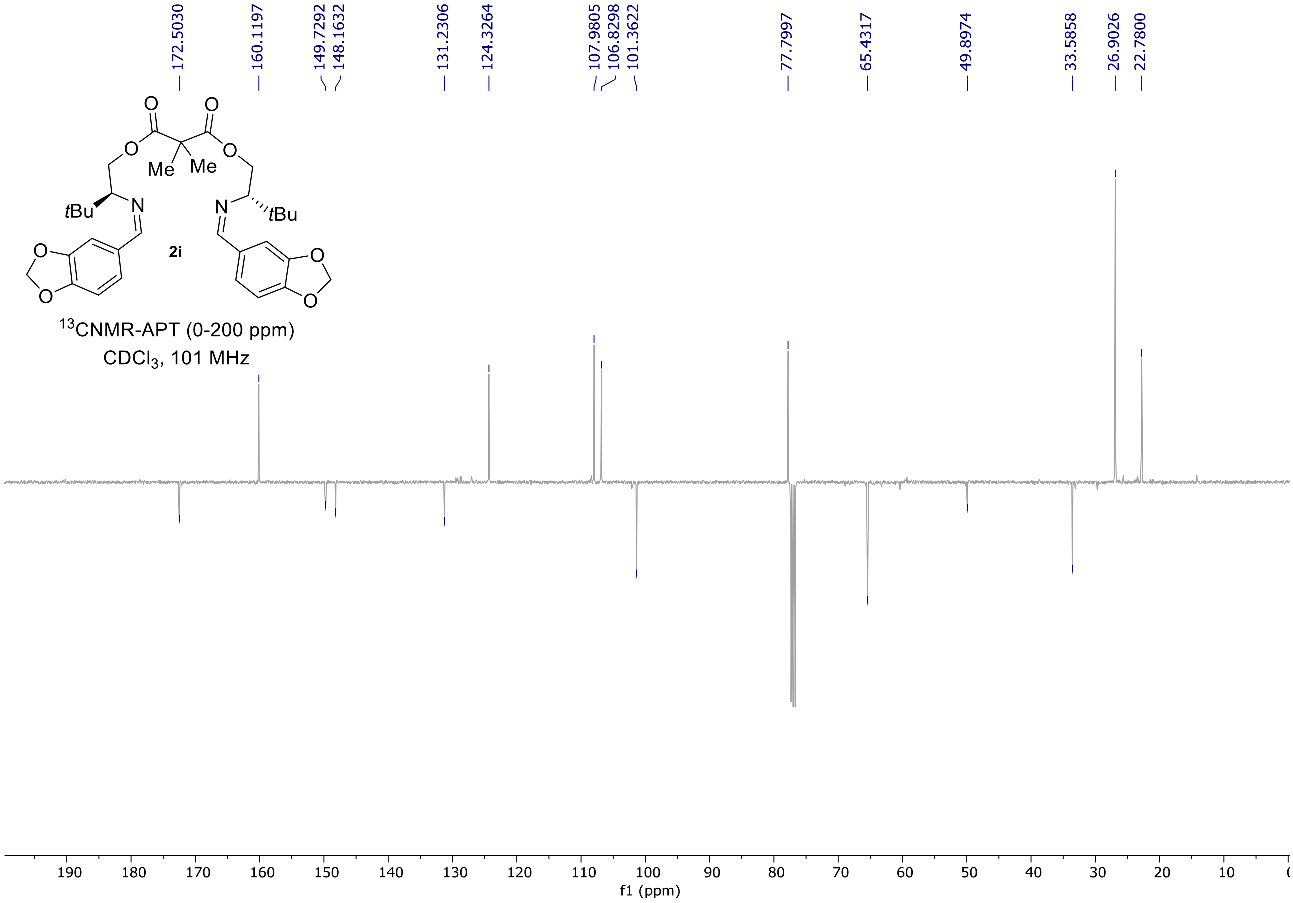

Bis((*S*)-3,3-dimethyl-2-((pyridin-4-ylmethylene)amino)*tert*-butyl) 2,2-dimethylmalonate (2j)

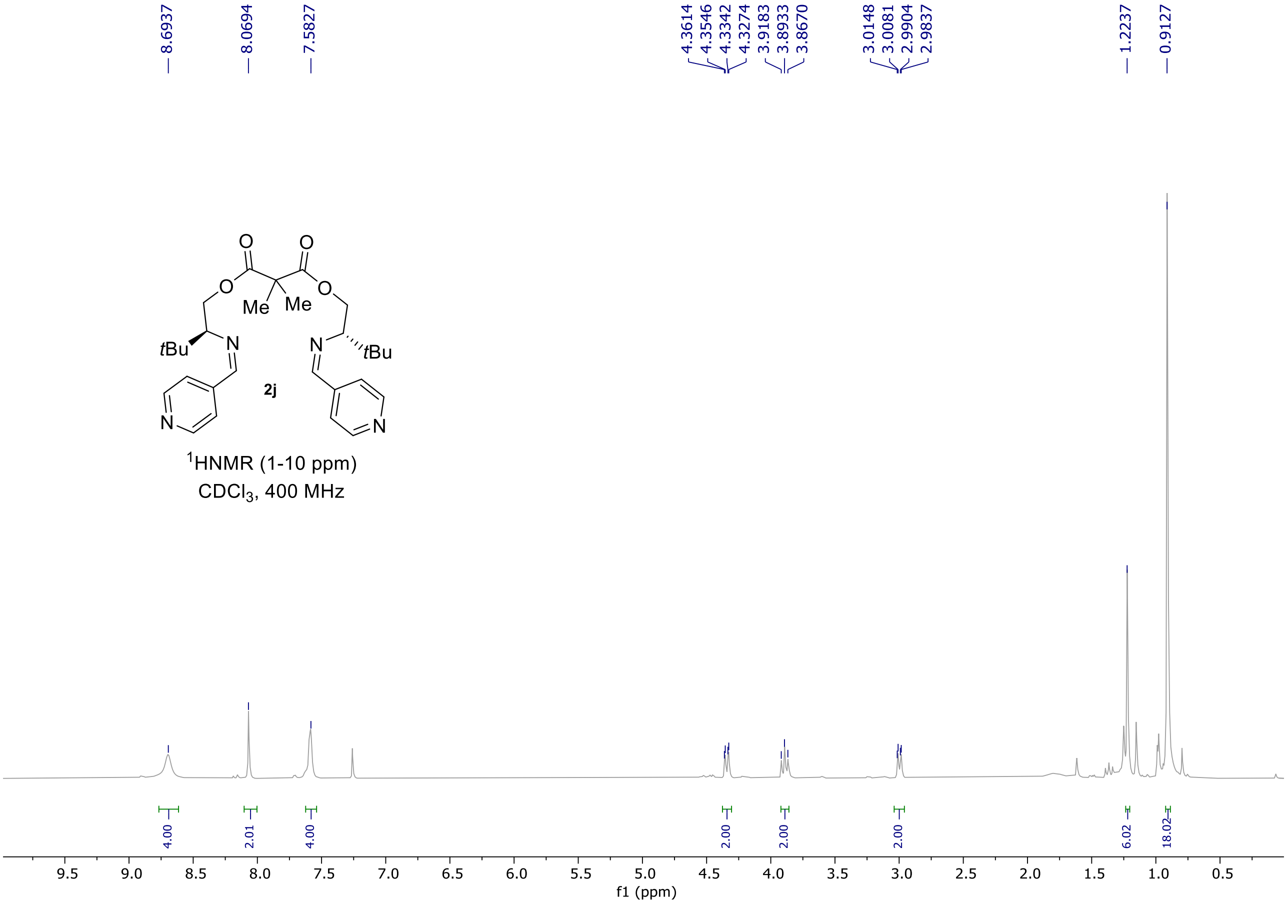

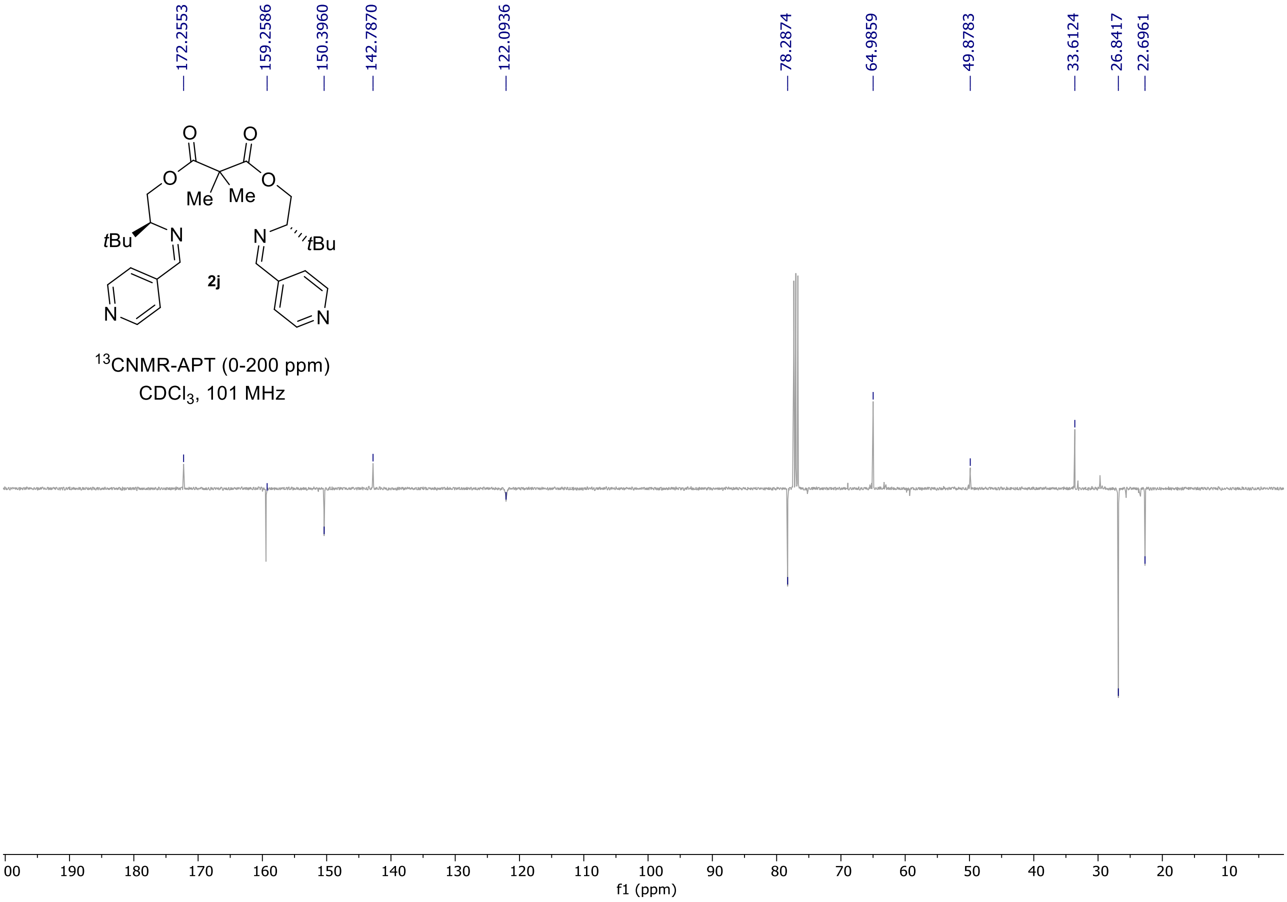

Bis((*S*)-3,3-dimethyl-2-((benzofuran-2-ylmethylene)amino)*tert*-butyl) 2,2-dimethylmalonate (2k)

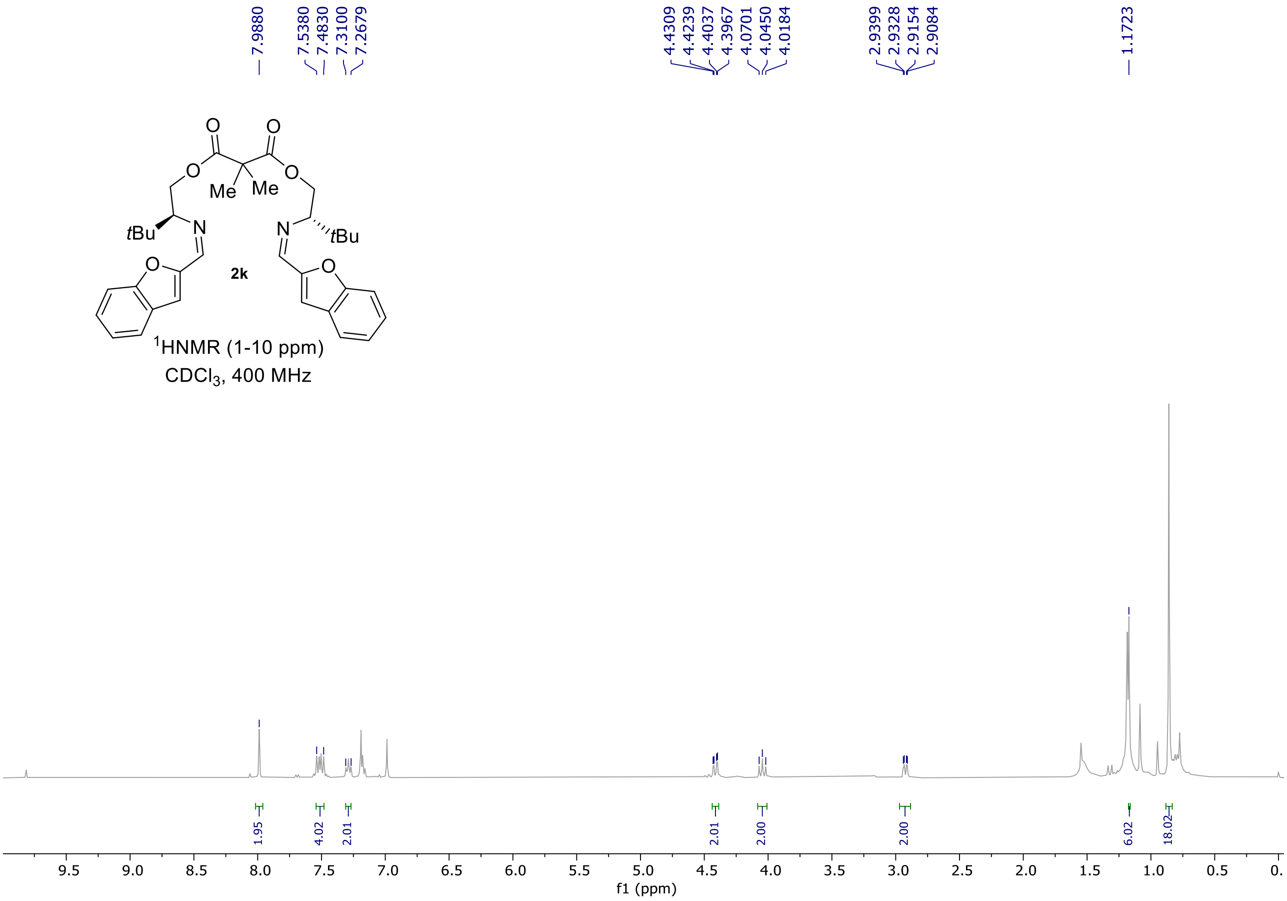

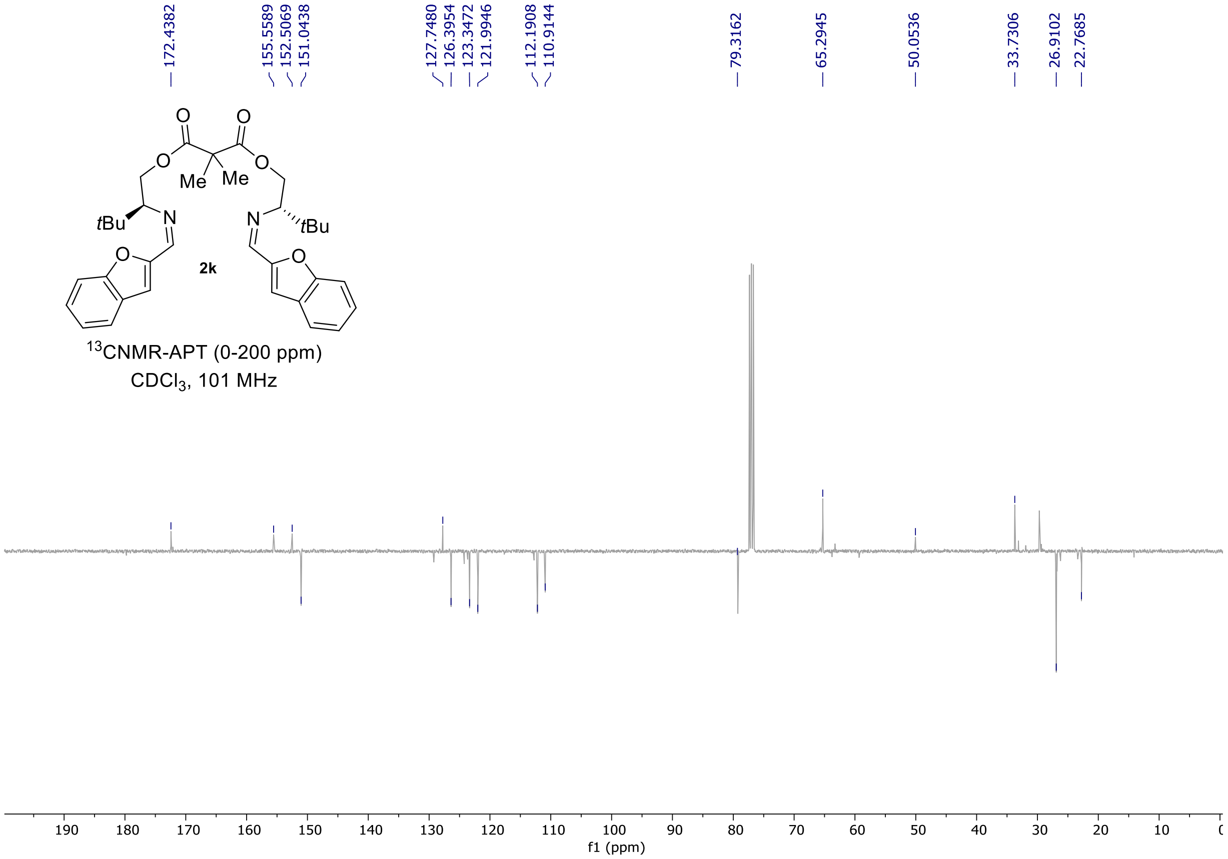

(*S*)-3,3-dimethyl-2-((thiophen-2-ylmethylene)amino)butyl 2-((S)-4-(*tert*-butyl)-4,5-dihydrooxazol-2-yl)-2-methylpropanoate (3)

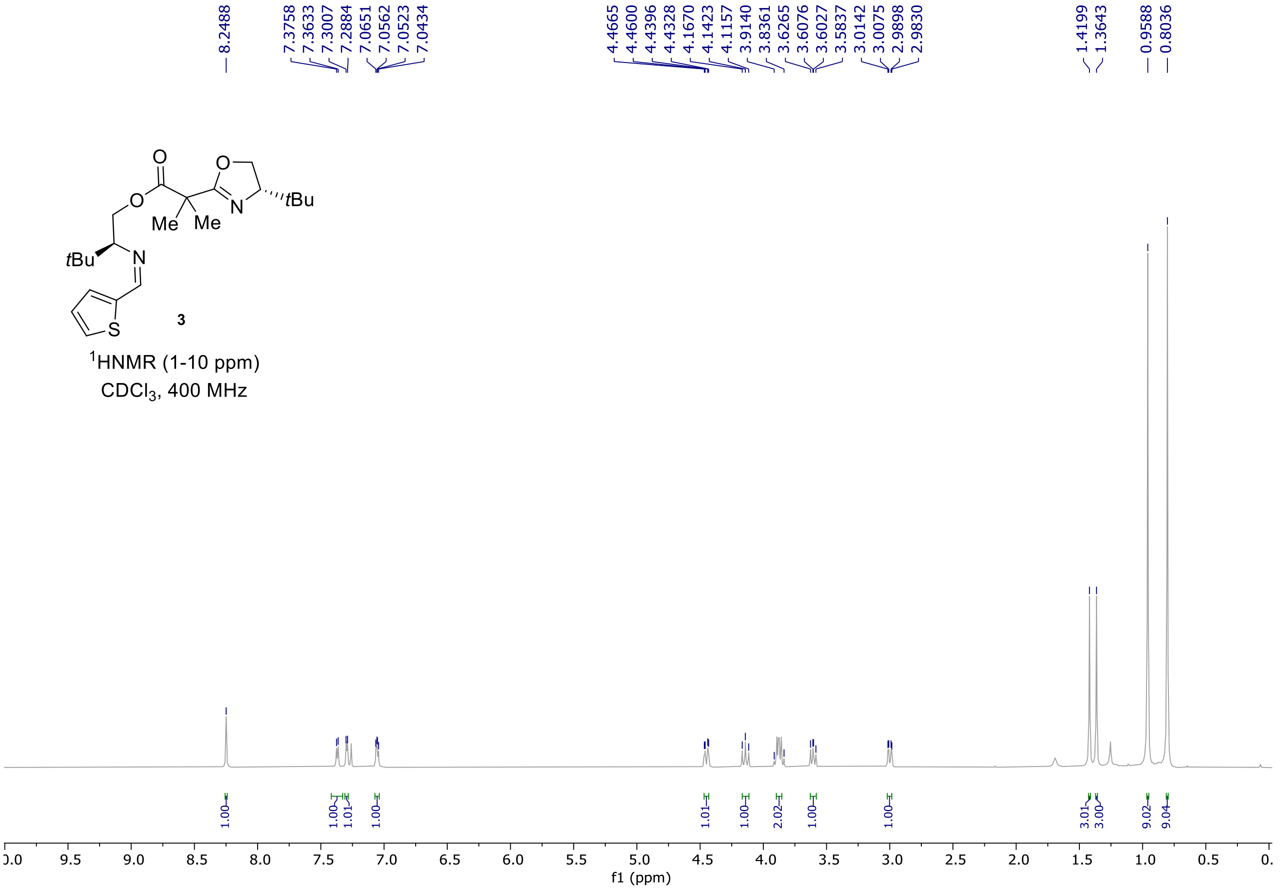

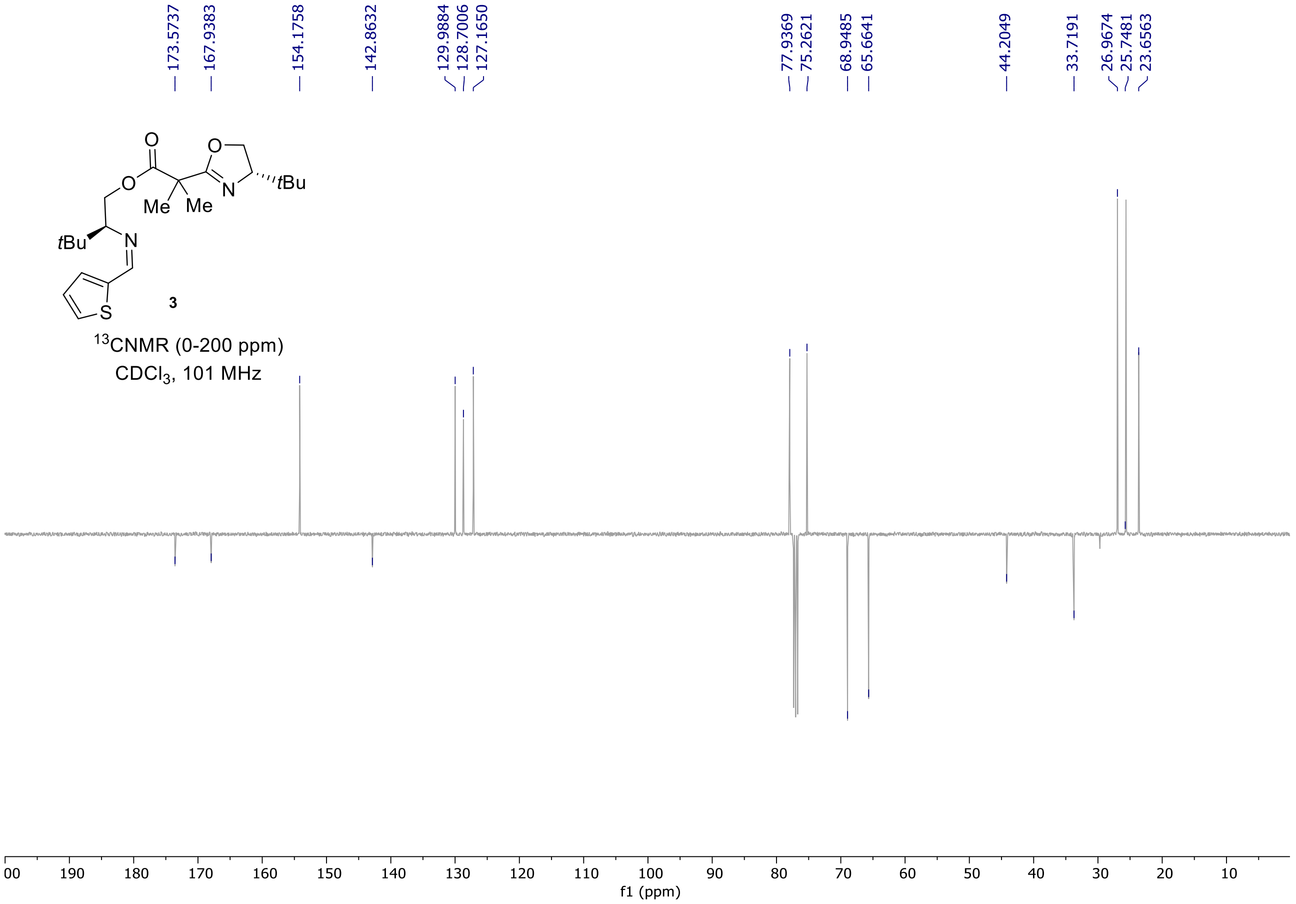

Bis((*S*)-3,3-dimethyl-2-((4-nitrobenzylidene)amino)*iso*-propyl) 2,2-dimethylmalonate (4)

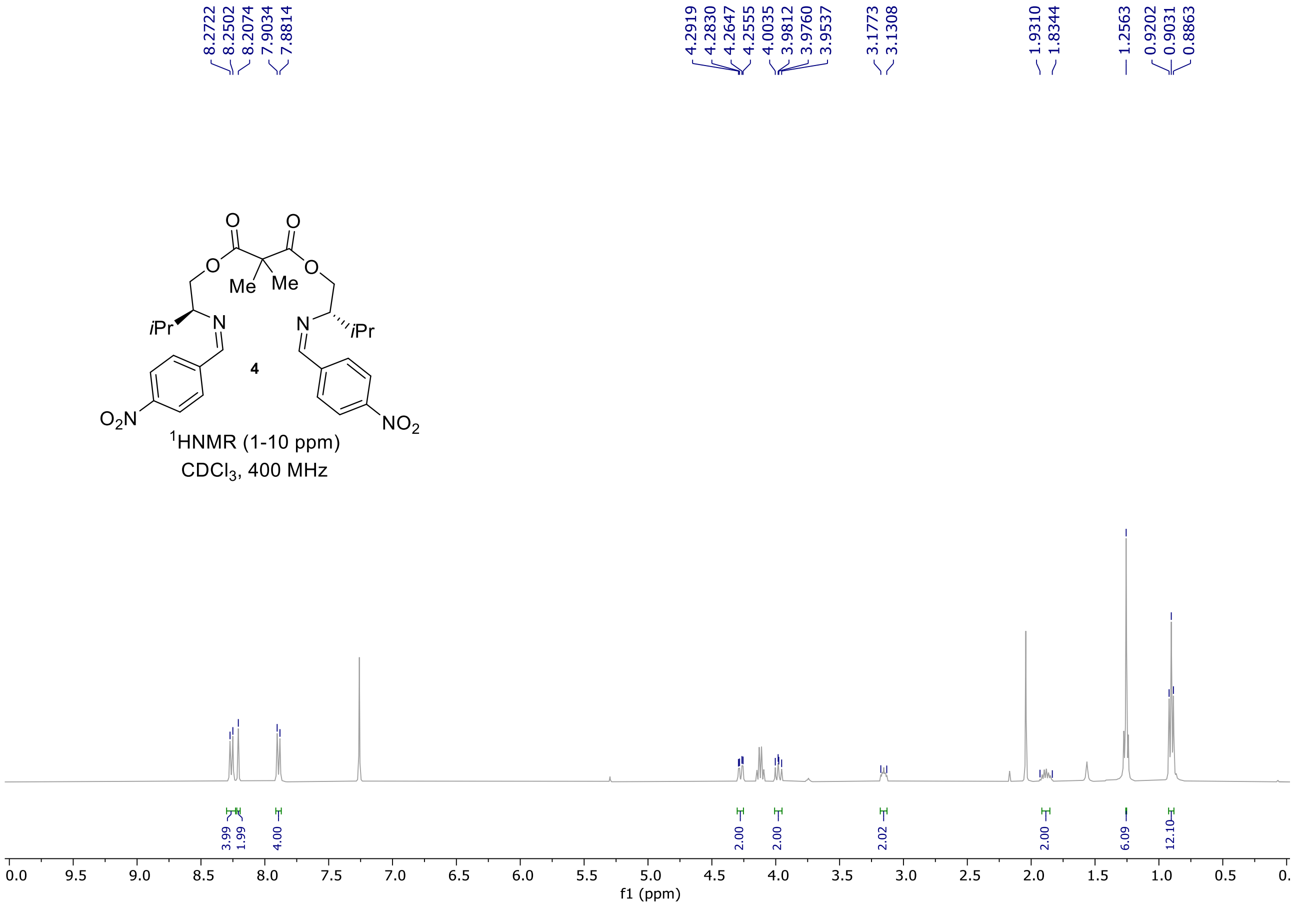

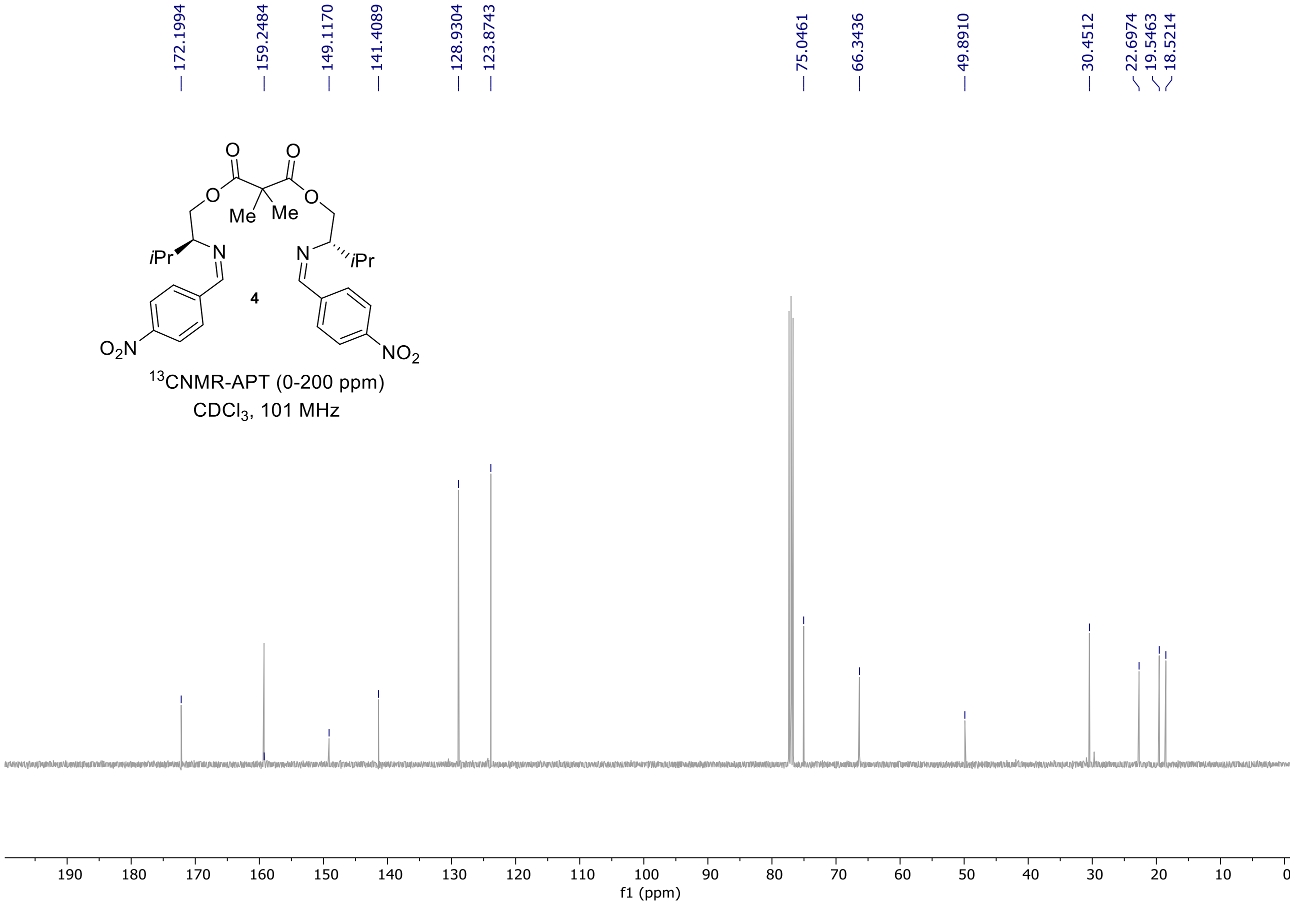

Bis((*S*)-3,3-dimethyl-2-((2-nitrobenzylidene)amino)*iso*-propyl) 2,2-dimethylmalonate (5)

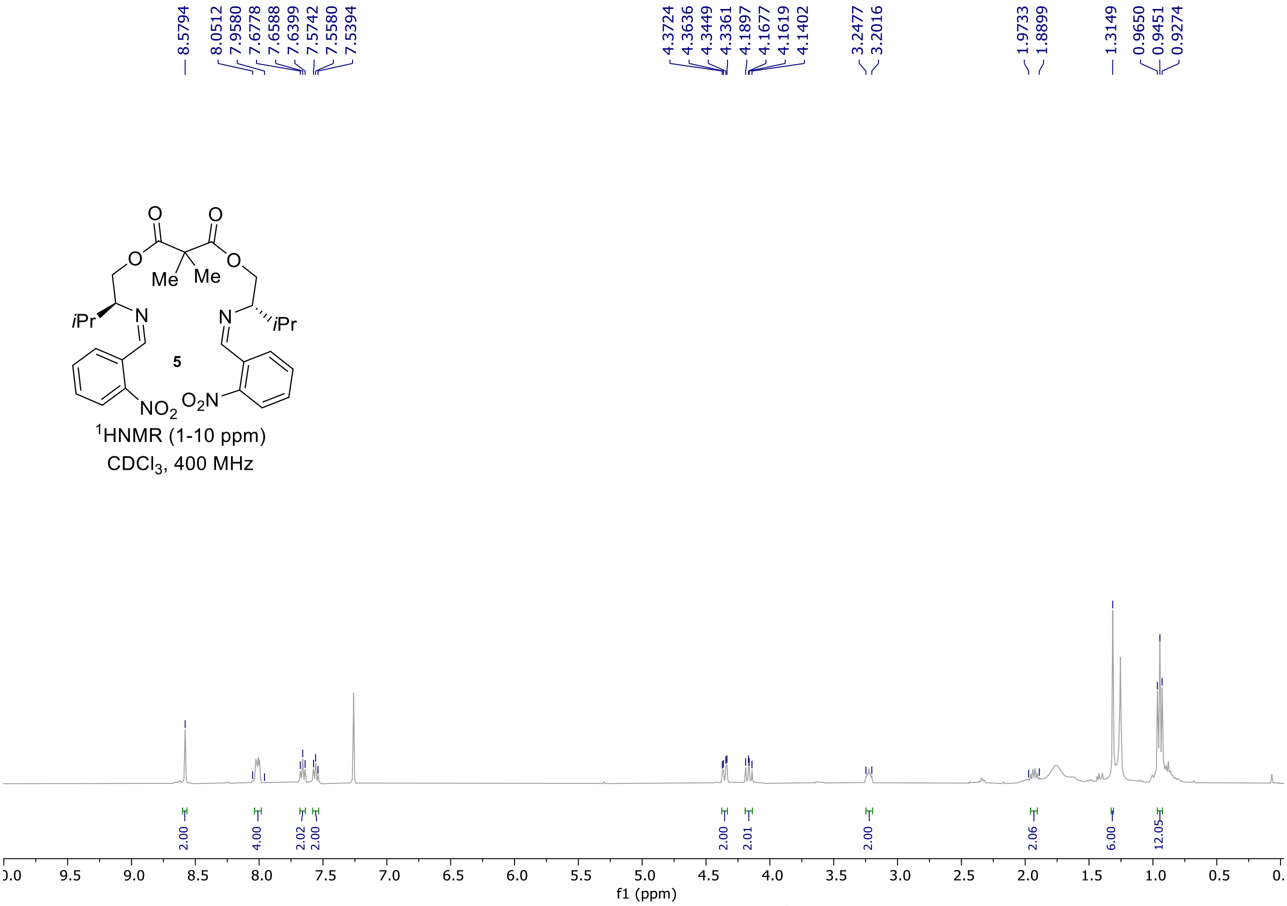

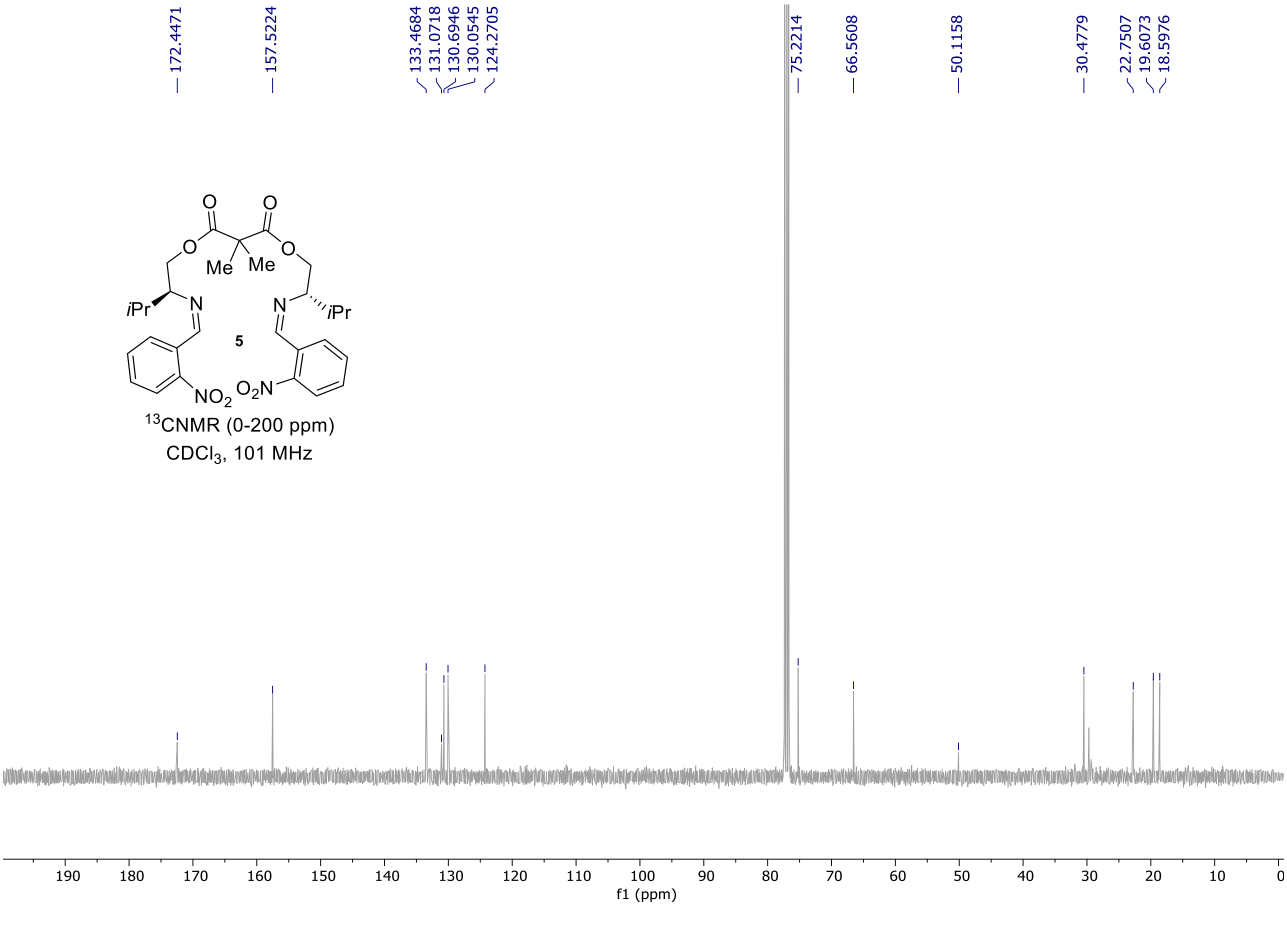

Bis((*S*)-3,3-dimethyl-2-((trifluoromethyl)benzylidene)amino)*iso*-propyl) 2,2-dimethylmalonate (6)

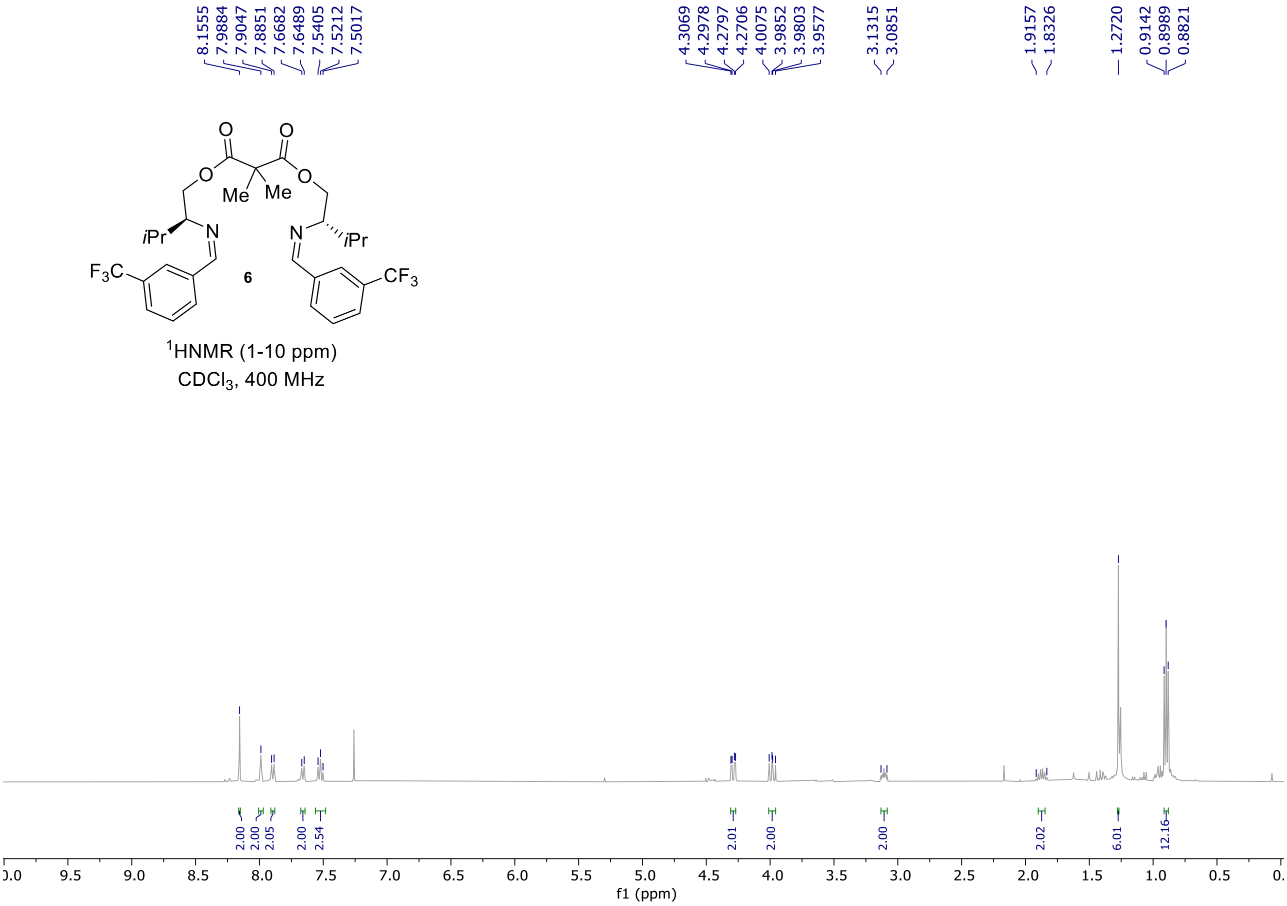

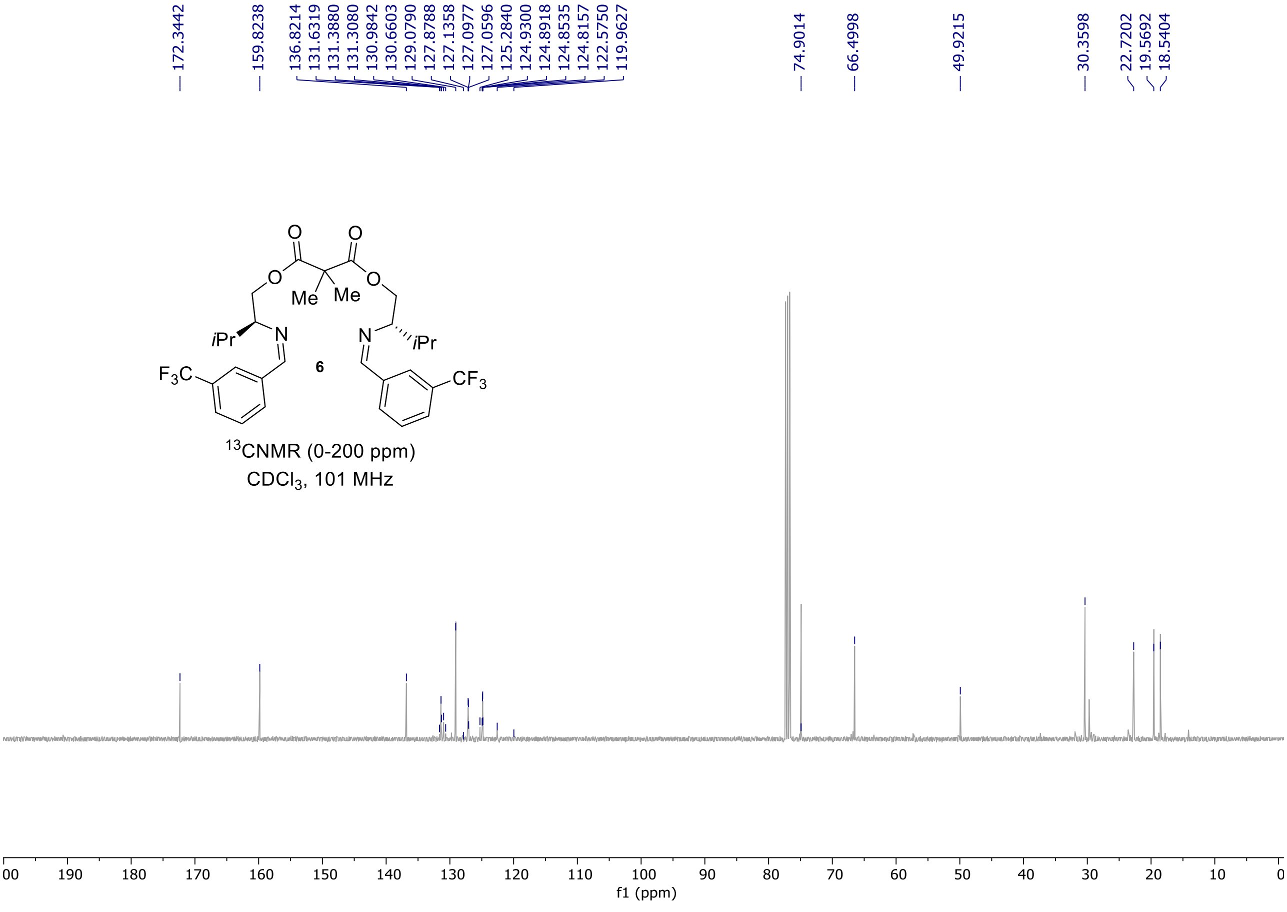

Bis((*S*)-3,3-dimethyl-2-(thiophen-2-ylmethylene)amino)*iso*-propyl) 2,2-dimethylmalonate (7)

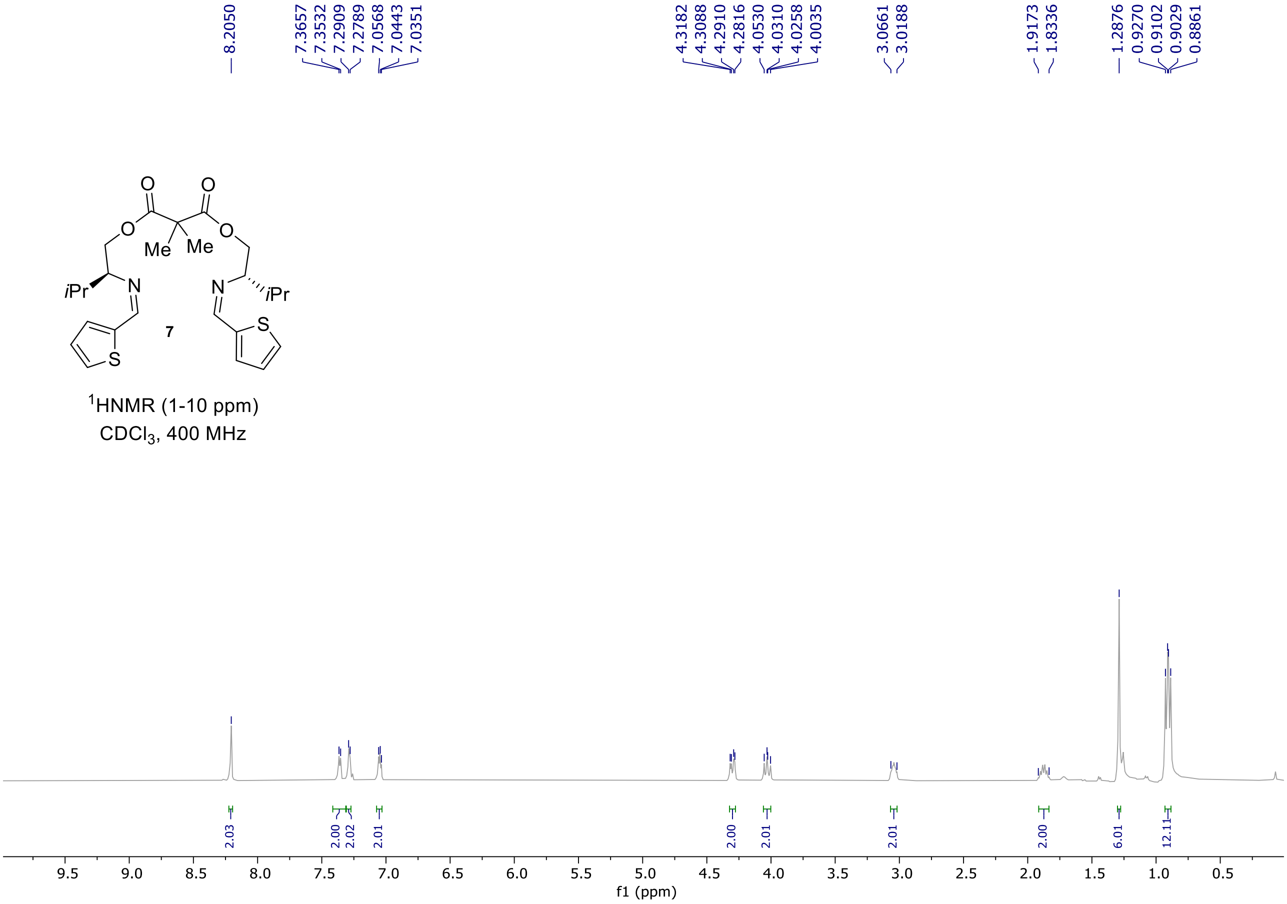

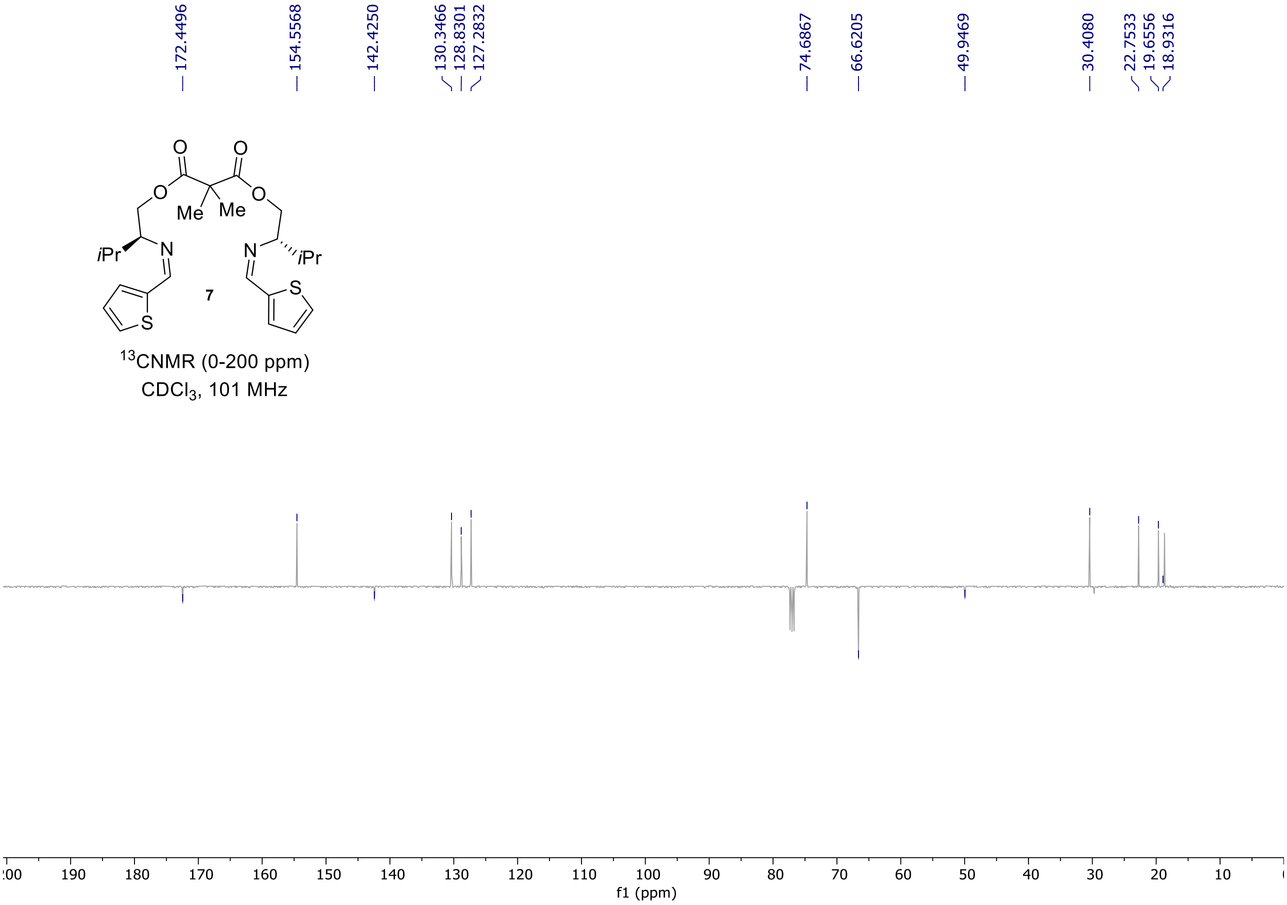

Bis((*S*)-3-phenyl-2-((thiophen-2-ylmethylene)amino)propyl) 2,2-dimethylmalonate (8)

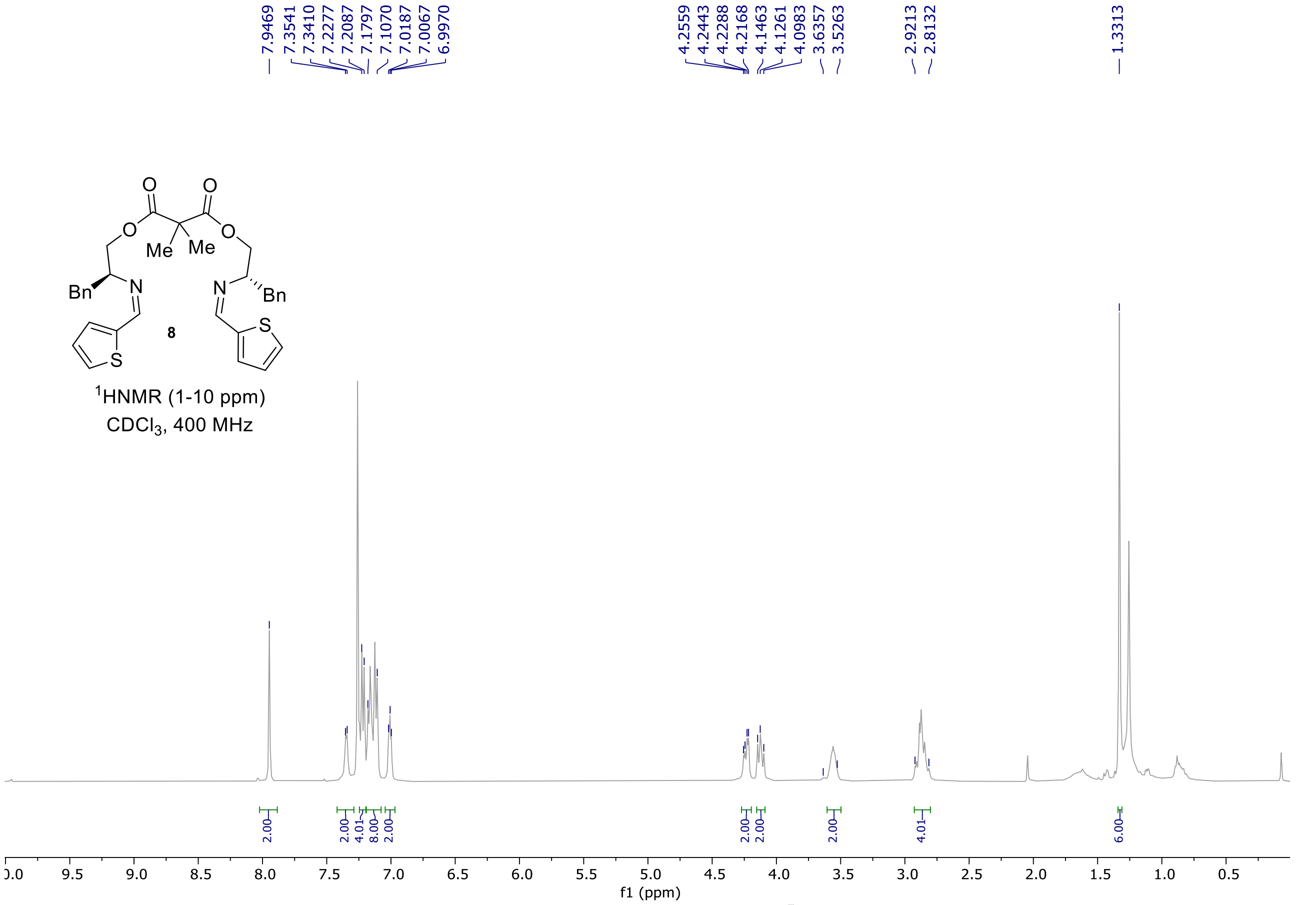

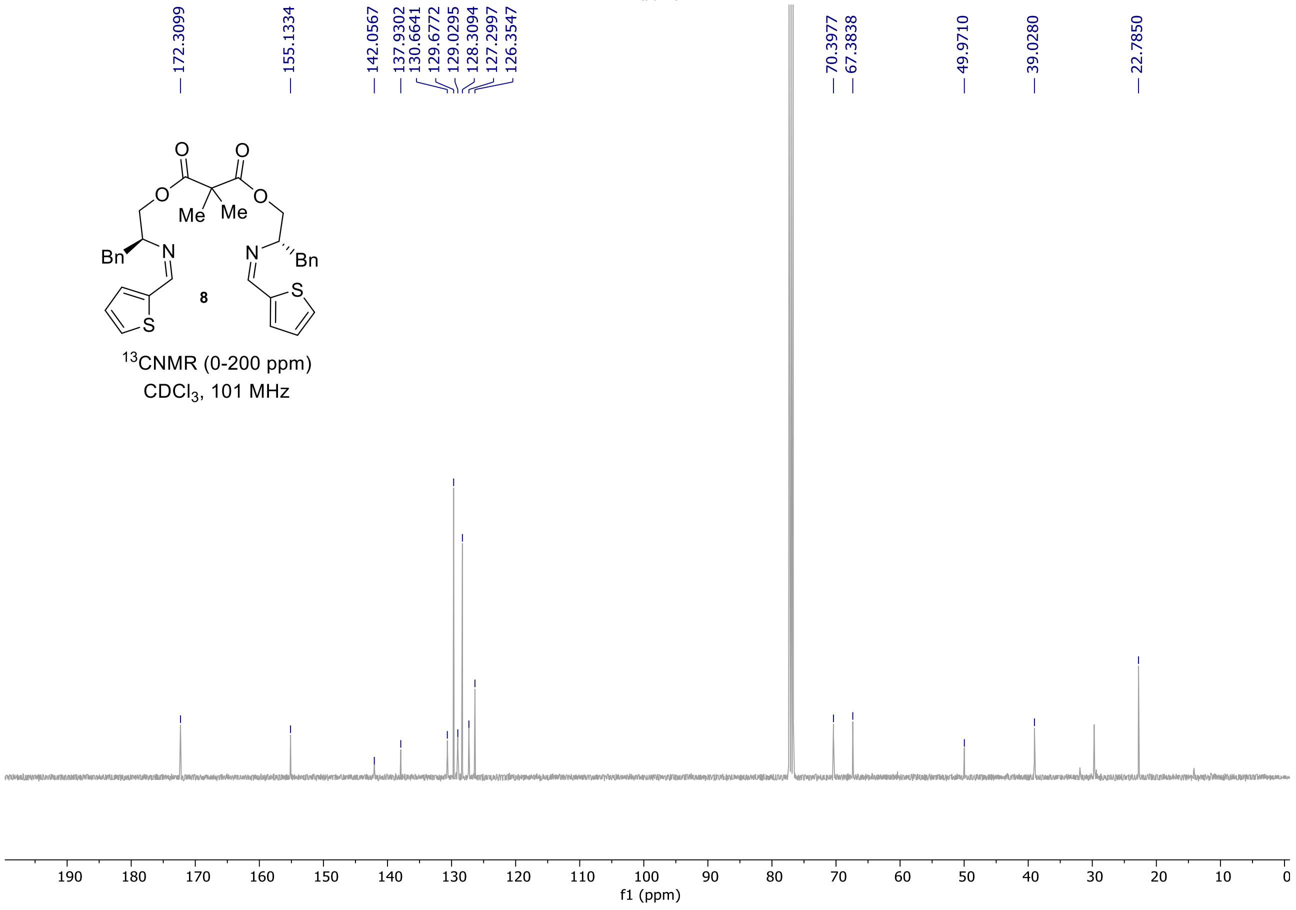

Bis((S)-2-((4-nitrobenzylidene)amino)-2-phenylethyl) 2,2-dimethylmalonate (9)

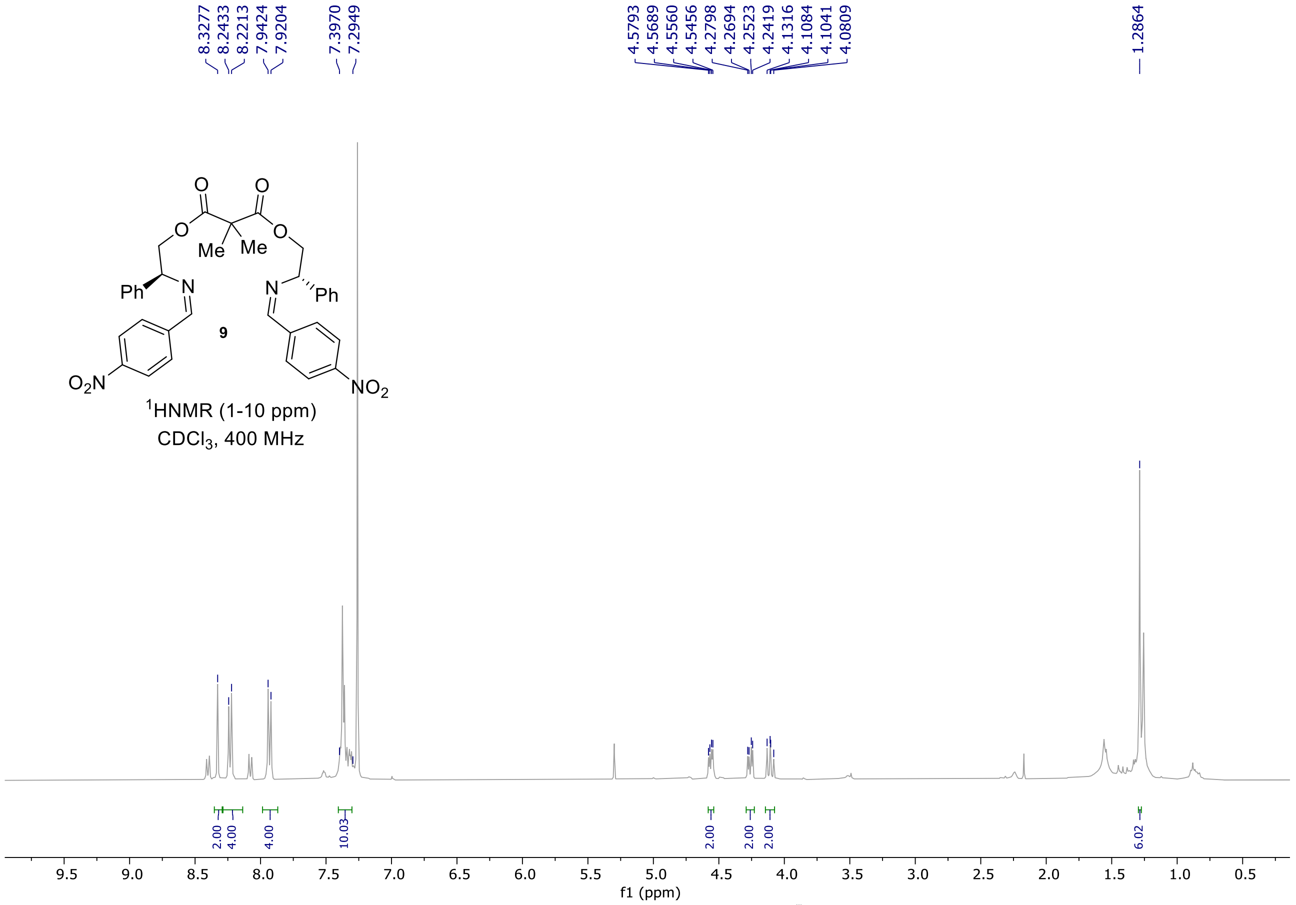

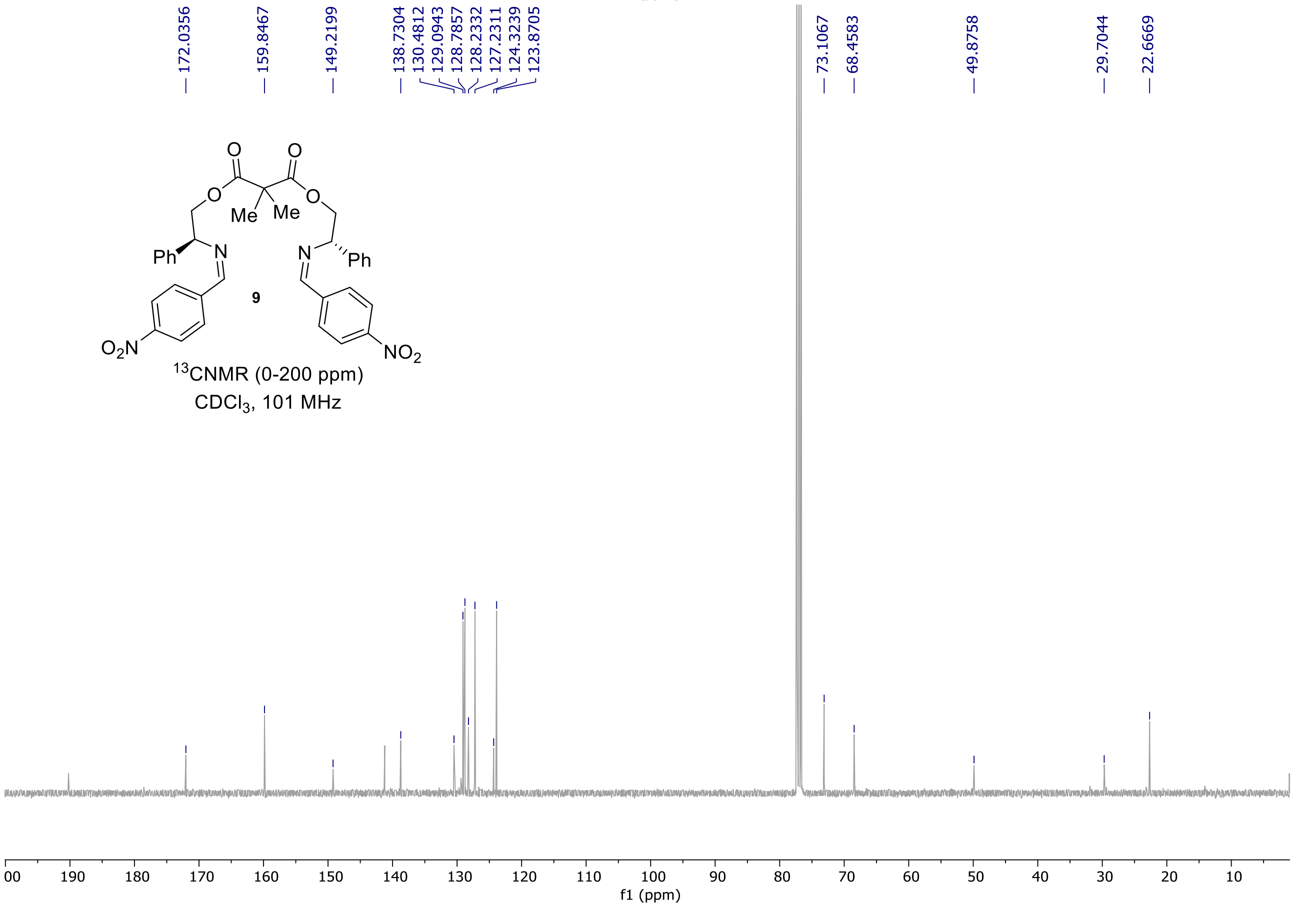

Bis((*S*)-2-((2-nitrobenzylidene)amino)-2-phenylethyl) 2,2-dimethylmalonate (10)

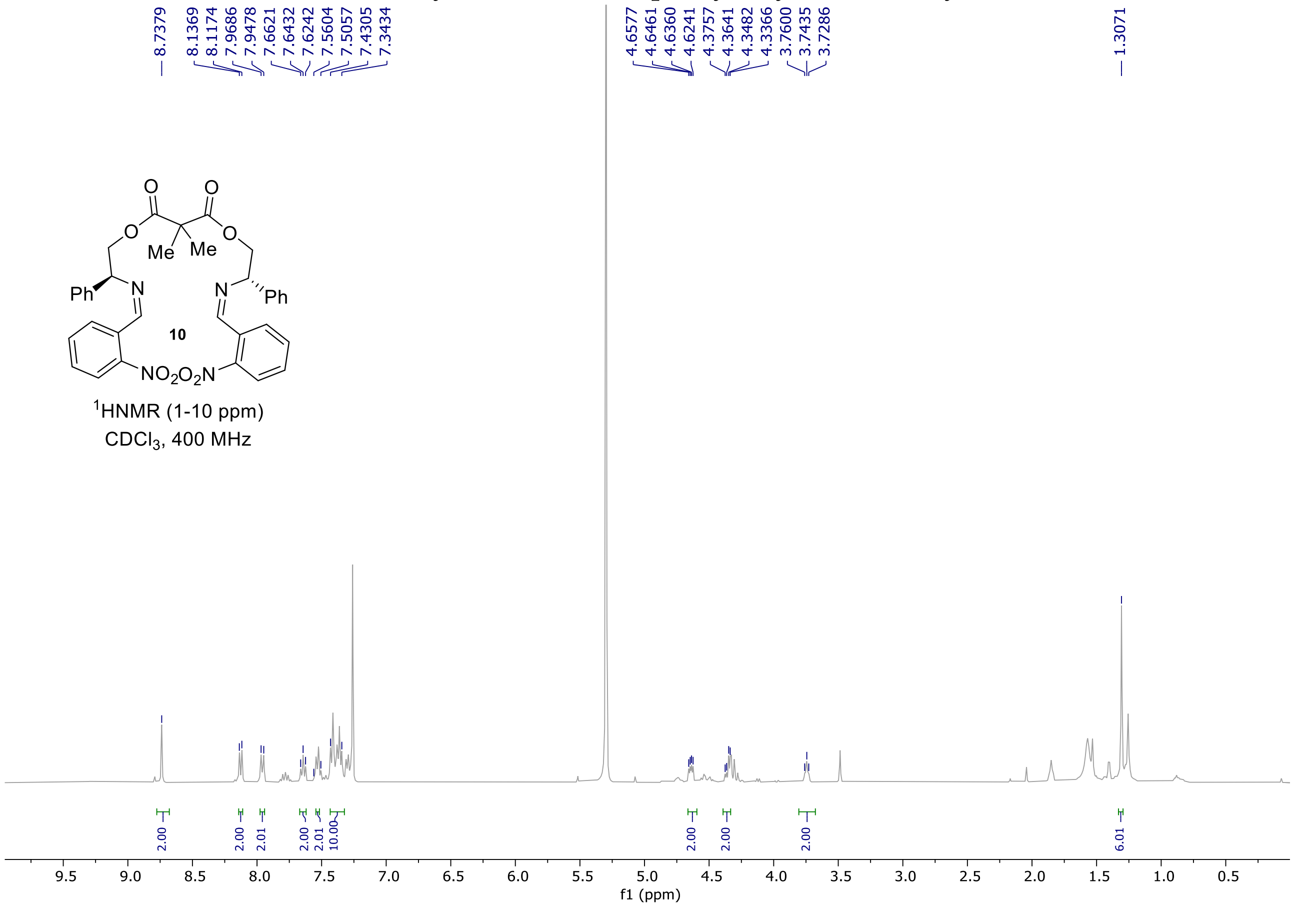

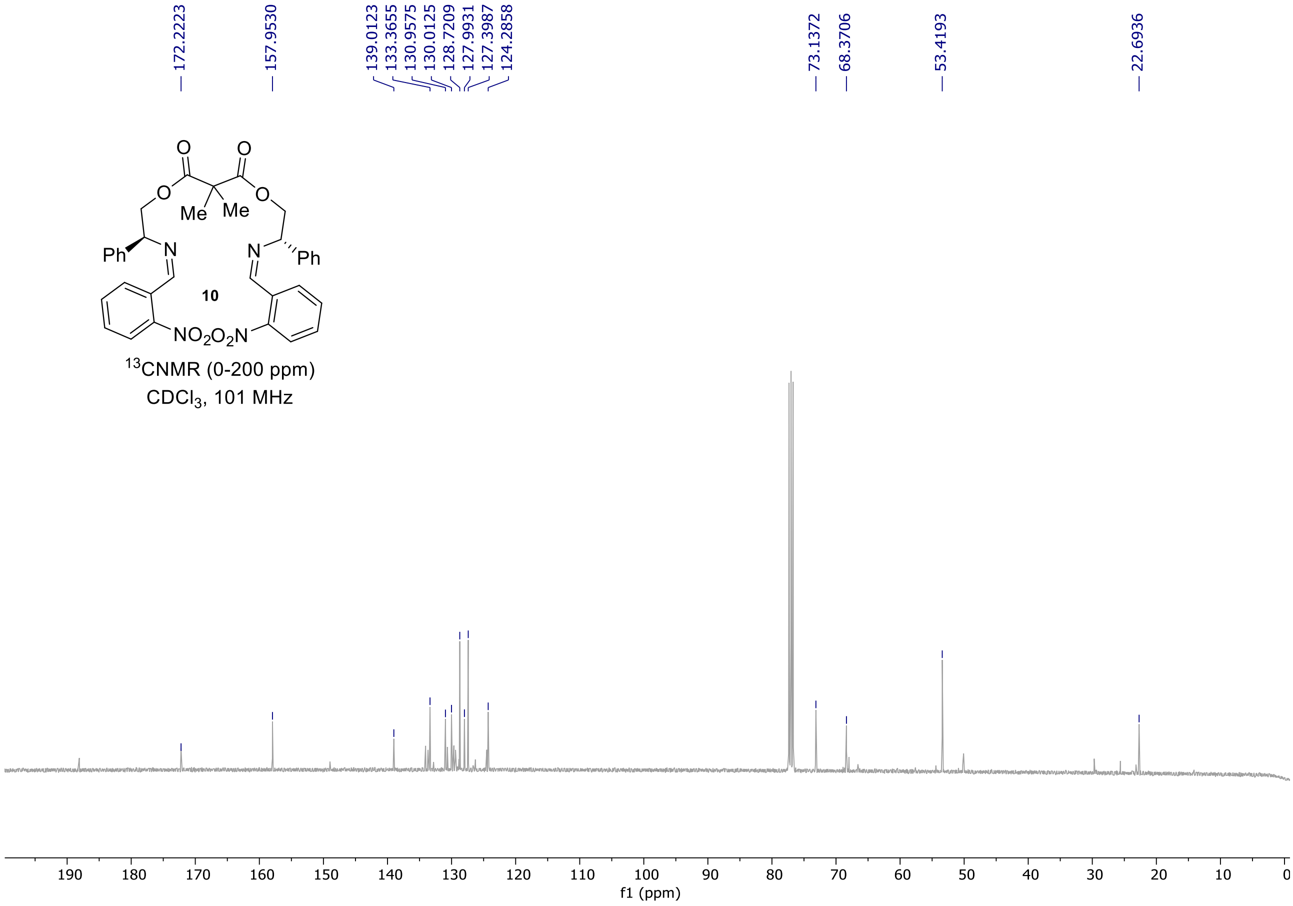

Bis((*S*)-3,3-dimethyl-2-(thiophen-2-ylmethylene)amino)*iso*-propyl) 2,2-dimethylmalonate (11)

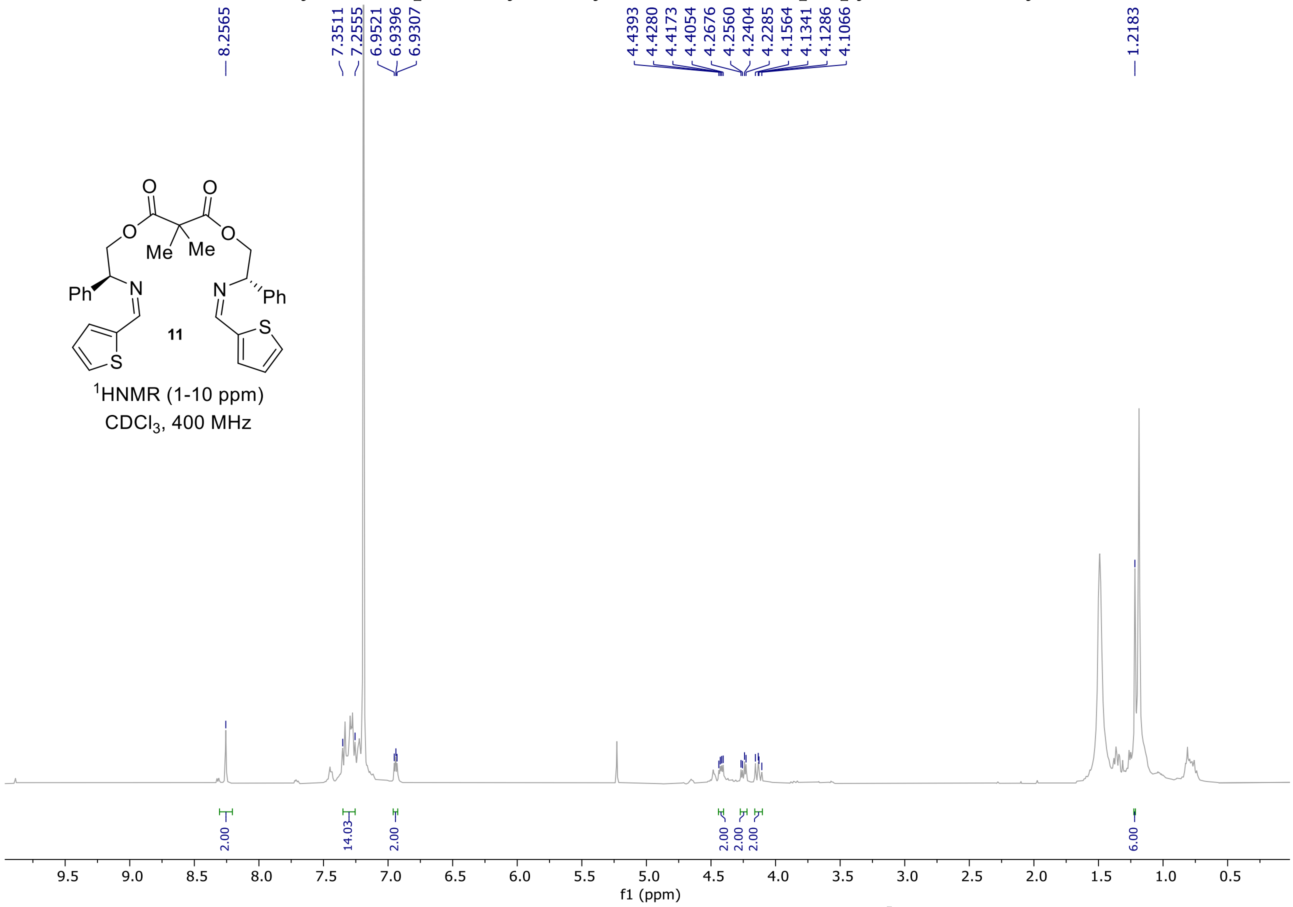

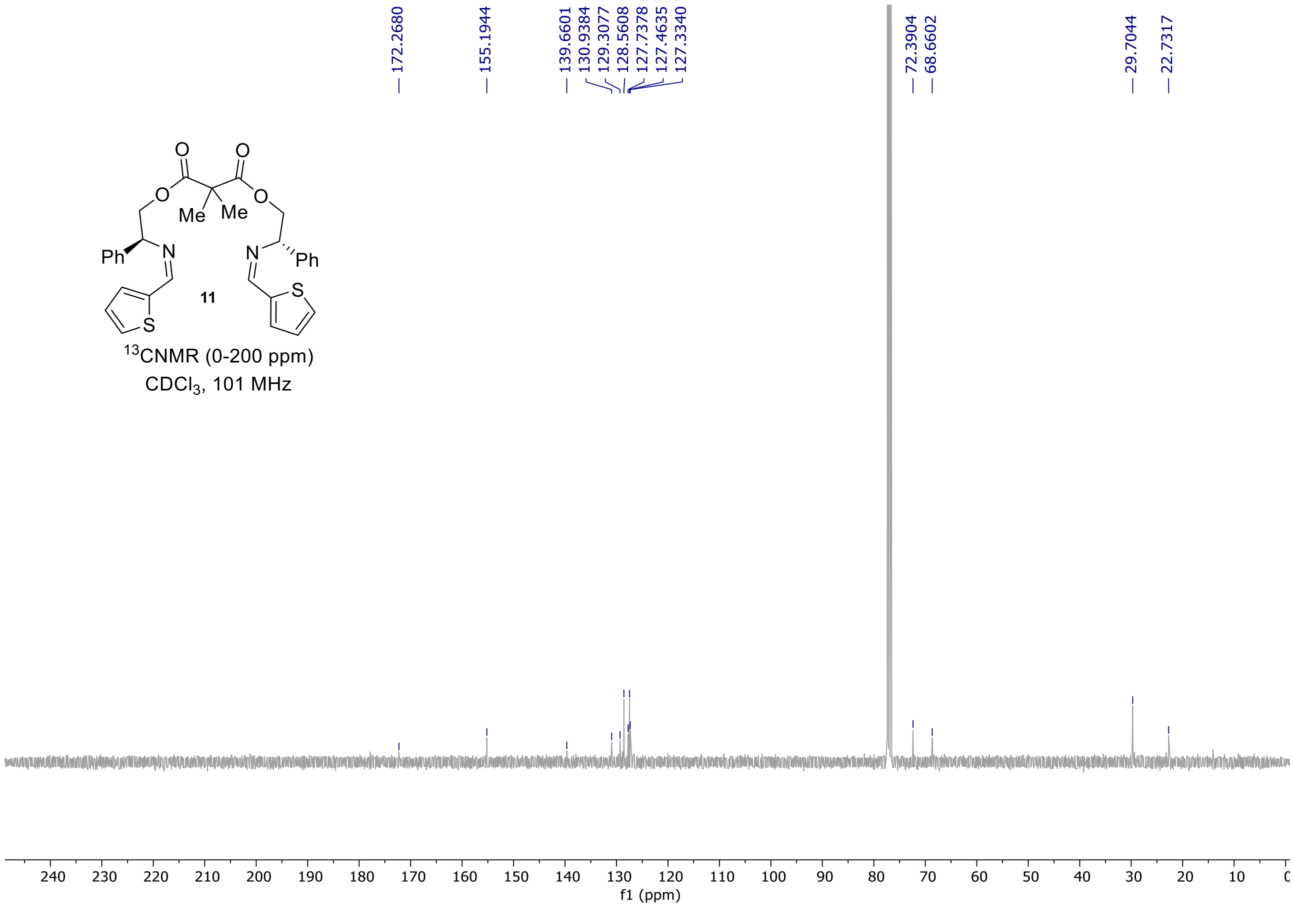

Bis((*S*)-3,3-dimethyl-2-((2-nitrobenzylidene)amino)butyl) pyridine-2,6-dicarboxylate (12)

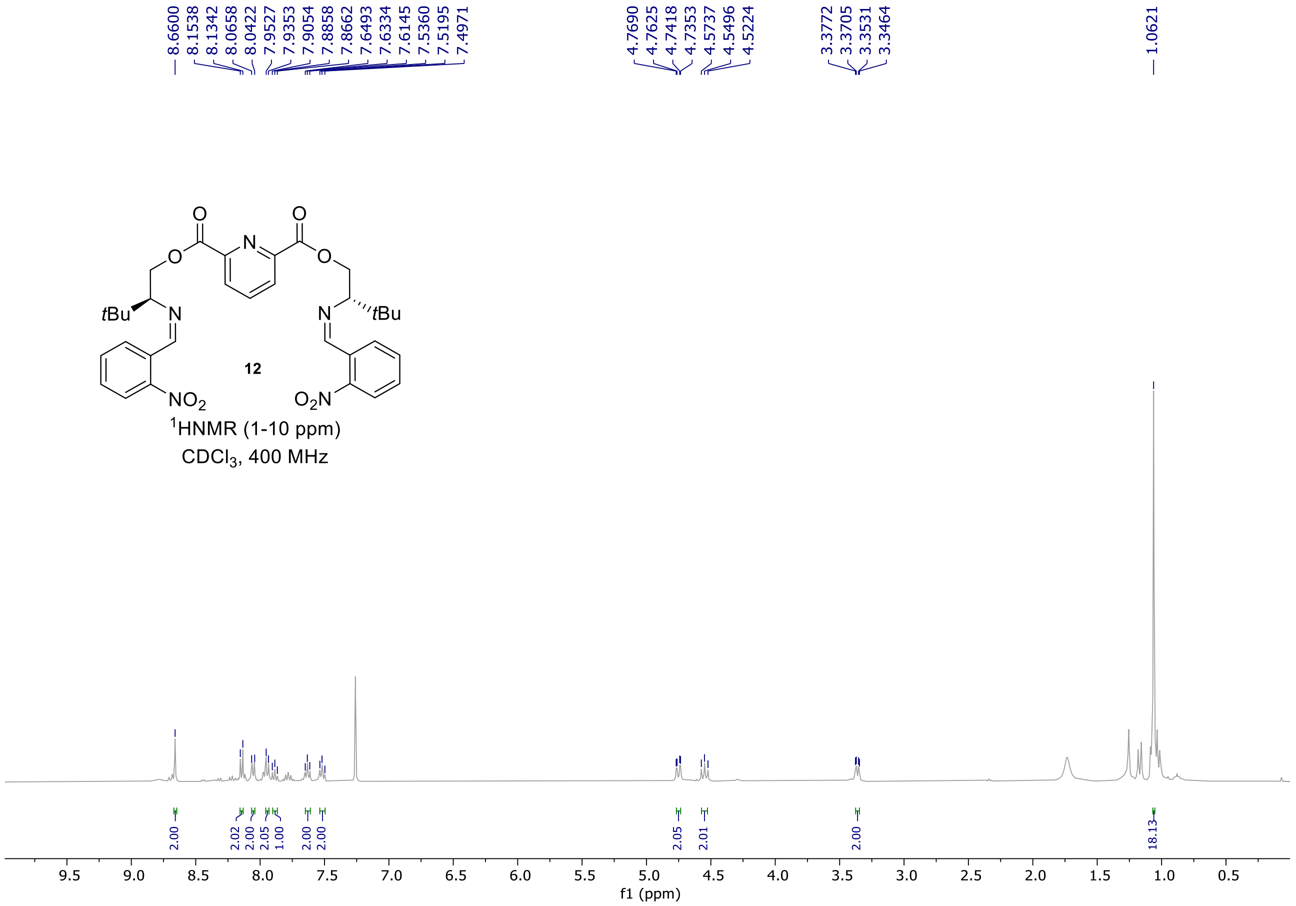

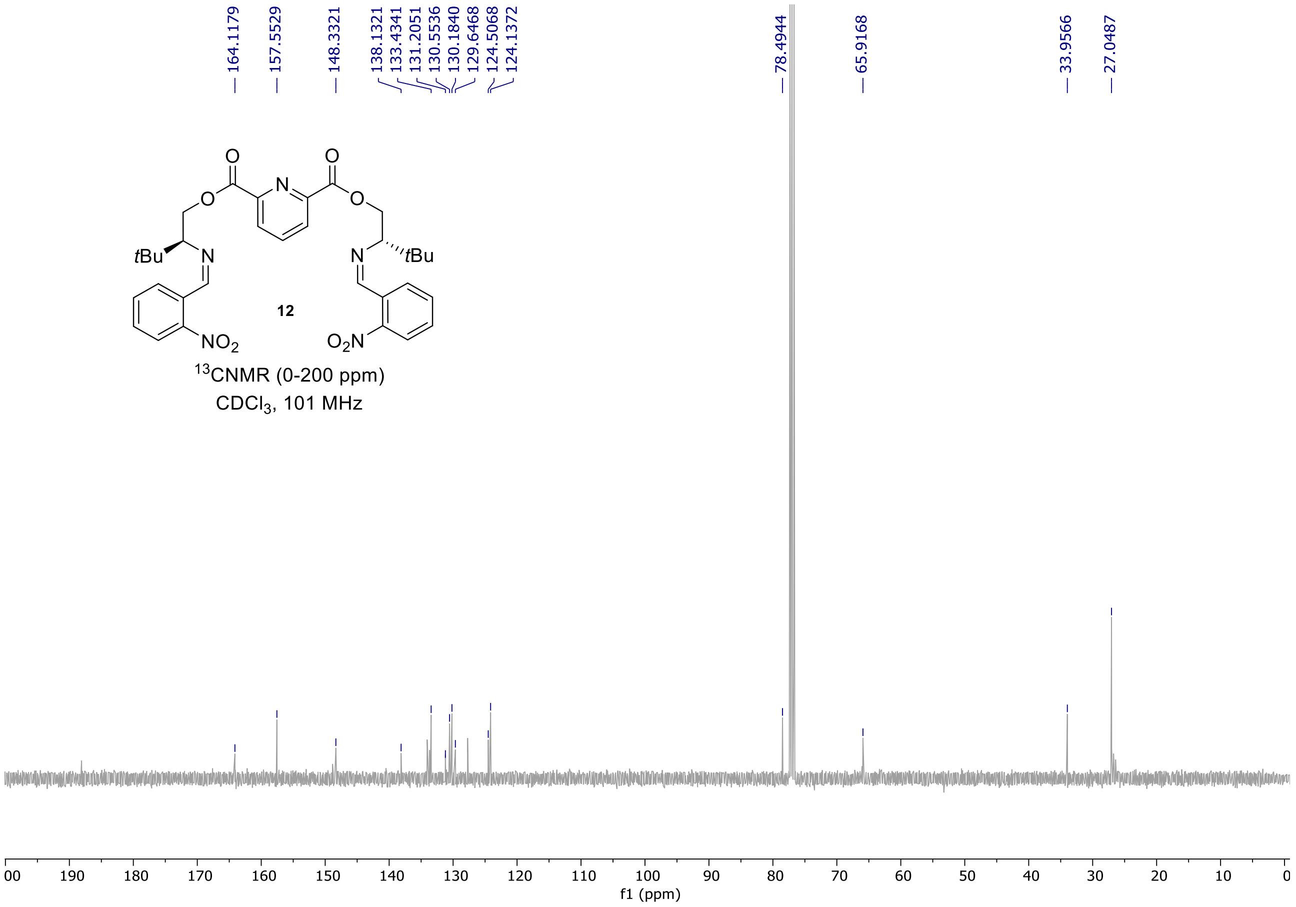

Bis(2-((thiophen-2-ylmethylene)amino)ethyl) isophthalate (13)

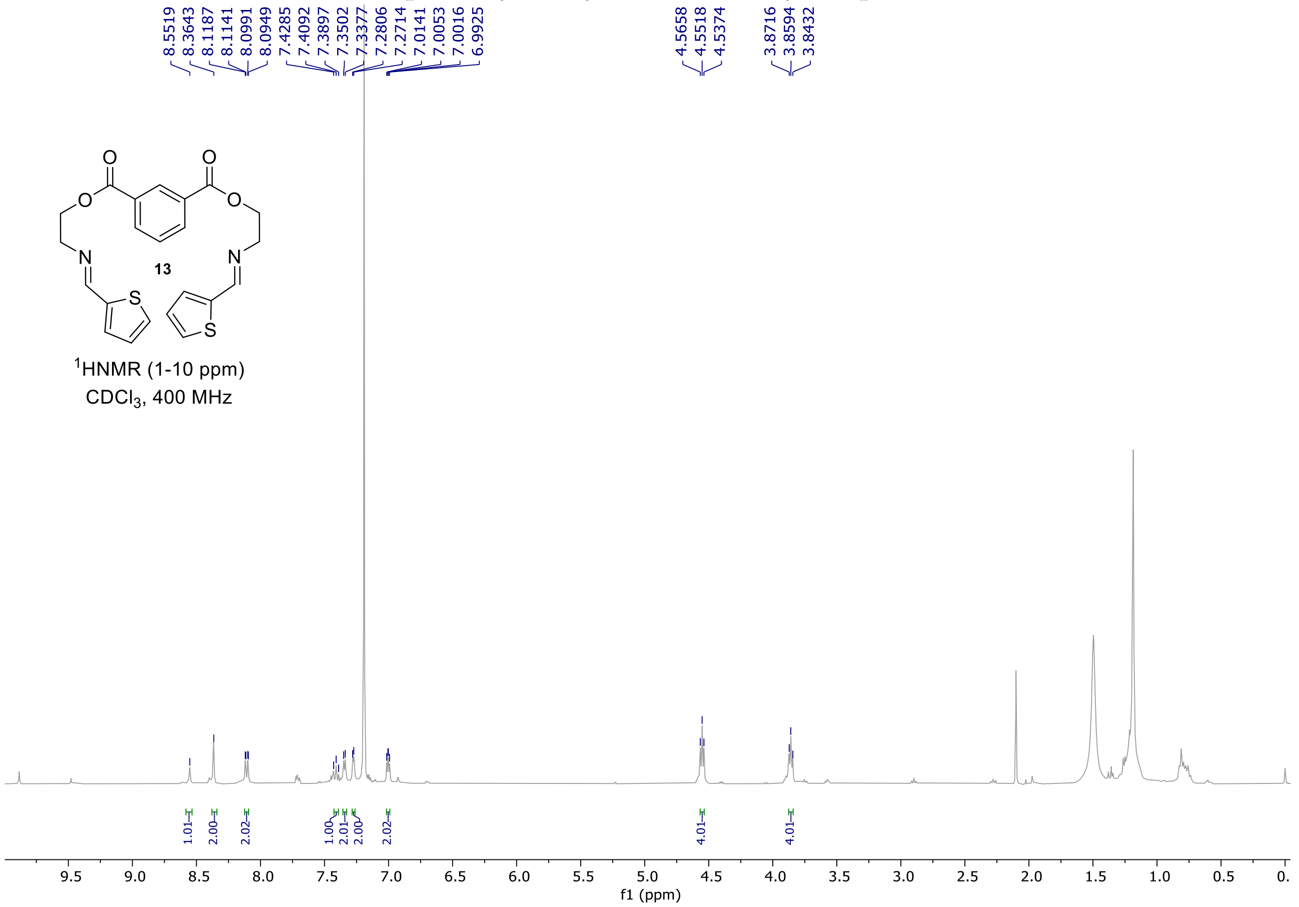

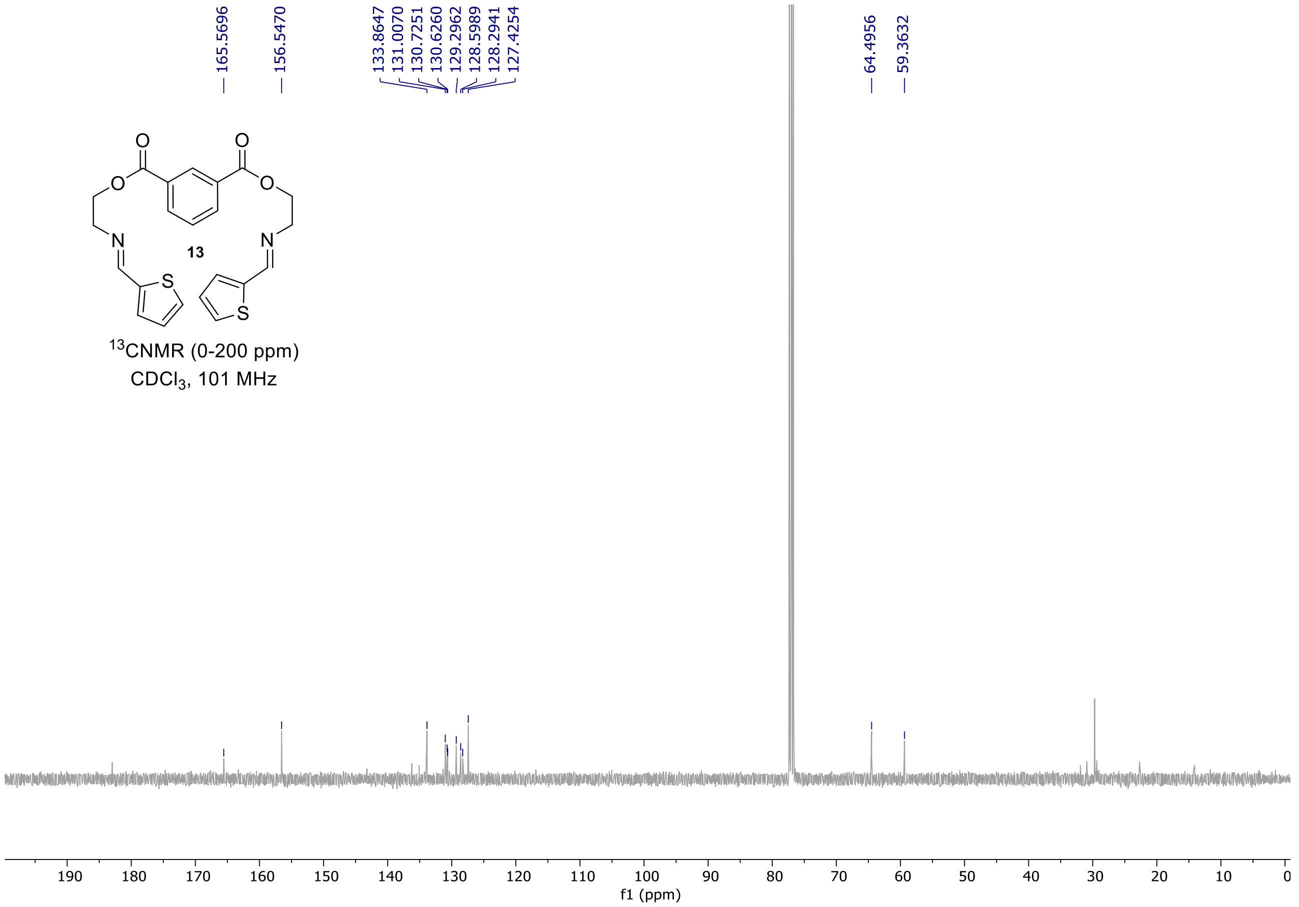

(*S*)-3-methyl-2-((thiophen-2-ylmethylene)amino)butyl benzoate (15)

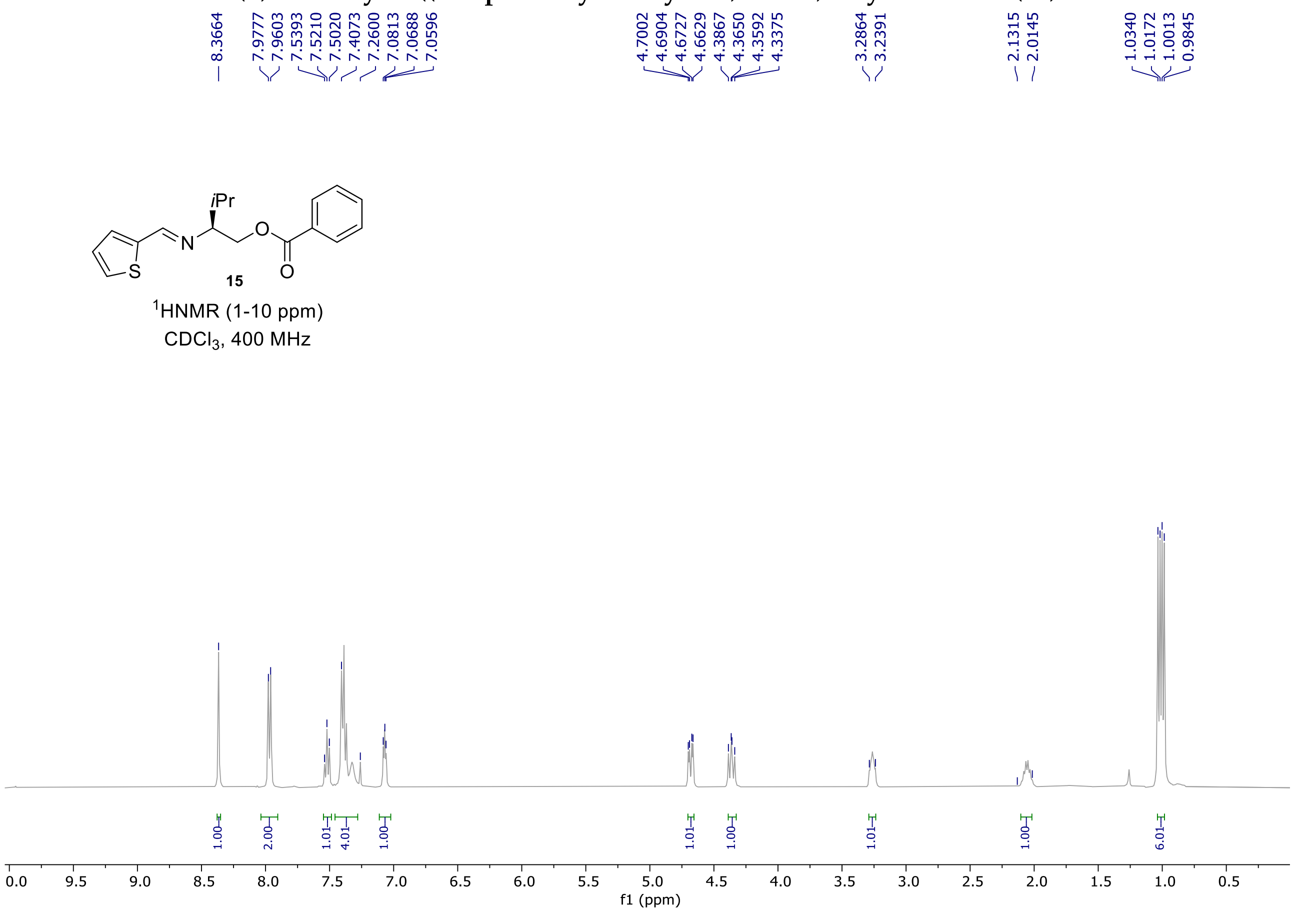

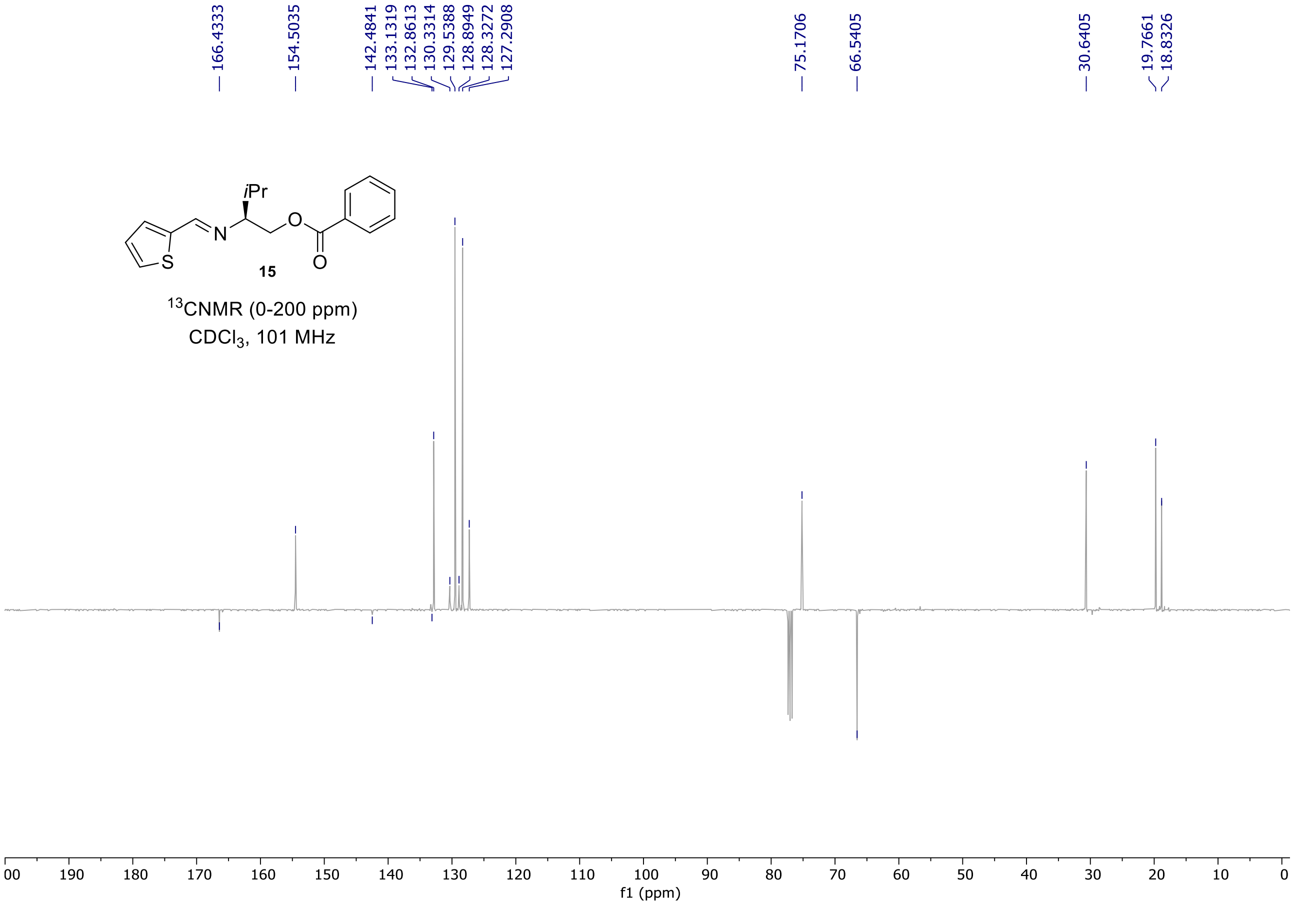

Supplement: Supplementary file 1 [file molecules-29-05756-s001.zip › molecules-3302168-supplementary.pdf]
